# Robust and efficient annotation of cell states through gene signature scoring

**DOI:** 10.1101/gr.280926.125

**Published:** 2026-03

**Authors:** Laure Ciernik, Agnieszka Kraft, Florian Barkmann, Josephine Yates, Valentina Boeva

**Affiliations:** 1 ETH Zurich, Department of Computer Science, Institute for Machine Learning, 8092 Zurich, Switzerland;; 2 Machine Learning Group, Technische Universität Berlin, 10587 Berlin, Germany;; 3 Hector Fellow Academy, 76131 Karlsruhe, Germany;; 4 SIB Swiss Institute of Bioinformatics, 1015 Lausanne, Switzerland;; 5 University Hospital Zurich, Department of Thoracic Surgery, 8092 Zurich, Switzerland;; 6 ETH AI Center, ETH Zürich, 8092 Zurich, Switzerland;; 7 Institut Cochin, Inserm U1016, CNRS UMR 8104, Université Paris Cité, 75014 Paris, France

## Abstract

Gene signature scoring is integral to single-cell RNA sequencing (scRNA-seq) data analysis, particularly for unsupervised cellular state annotation based on maximum signature score values. However, this application requires robust and comparable score distributions across diverse signatures and experimental conditions. Our systematic evaluation of established scoring methodologies—Seurat, SCANPY, UCell, and JASMINE—across nine healthy and cancer scRNA-seq data sets demonstrates their insufficiency in fulfilling this requirement. To address this limitation, we present Adjusted Neighborhood Scoring (ANS), a deterministic algorithm with enhanced control gene selection that significantly improves score stability and cross-signature comparability, achieving cell-state annotation accuracy comparable to supervised methods. We demonstrate the practical utility of ANS by developing and validating a gene signature to differentiate cancer-associated fibroblasts from malignant cells undergoing epithelial-to-mesenchymal transition. Overall, ANS provides a robust and reliable gene signature scoring framework, significantly improving the accuracy of score-based annotation of cell types and states in single-cell studies.

High-throughput single-cell RNA sequencing (scRNA-seq) is a powerful technology to profile the transcriptome at the cellular level, allowing for quantifying cell types and states, analyzing inter- and intrasample heterogeneity, discovering cell differentiation trajectories, and constructing gene regulatory networks (Zhao et al. 2022). Interpretation of such data can be challenging owing to high dimensionality, batch effects, dropout, and transcriptional noise (Lopez et al. 2018); therefore, scRNA-seq data analysis methods must address the inherent variability and noise in these data. This is especially important when evaluating cell states and programs through gene signature scoring.

Gene signature scoring in scRNA-seq data measures the activity of biological processes represented by predefined gene signatures. Such signatures are sets of genes associated with specific biological pathways, transcriptional states, or cell types and are used as a surrogate representation for a biological phenotype (Nevins and Potti 2007). One key application of signature scoring is cell annotation, as it offers a highly efficient and reliable method for classifying cells into types and states (Neftel et al. 2019). Notably, the quality of gene signatures plays a critical role in this process, as the accuracy of the score depends on how well the selected genes reflect the underlying biological process, regardless of the performance of the scoring method itself.

Numerous techniques for gene signature scoring in bulk RNA-seq and scRNA-seq have been developed lately. Recent studies, however, showed that methods created for bulk RNA-seq, such as ssGSEA (Barbie et al. 2009) and GSVA (Hänzelmann et al. 2013), are not fit for scRNA-seq data because they are more susceptible to dropouts and suffer from imbalanced expressions of genes in cancer cells versus nonmalignant cells in tumor samples (Noureen et al. 2022). To overcome the limitations of applying these methods to scRNA-seq data, Gibbs et al. (2023) proposed a data preprocessing step, which smoothes the gene expression matrix before gene signature scoring with bulk-based methods. Although such smoothing reduces noise, it is important to note the trade-off between removing variance and introducing bias in single-cell studies (Huang et al. 2018; Li and Li 2018). Given these limitations, researchers were advised to use single-cell-specific methods, such as signature scoring methods of SCANPY (Wolf et al. 2018), Seurat (Satija et al. 2015), UCell (Andreatta and Carmona 2021), and Jointly Assessing Signature Mean and Inferring Enrichment (JASMINE) (Noureen et al. 2022).

The scoring methods implemented in the scRNA-seq analysis packages SCANPY (Wolf et al. 2018) and Seurat (Satija et al. 2015) are based on a procedure described by Tirosh et al. (2016), which computes scores by averaging the difference in expression of the signature and control genes. The control genes are selected by binning genes based on their mean expression across cells; therefore, the scores may be biased by the number of bins and the behavior of the mean expression curve. This often leads to wide-range intervals in the bins at the distribution tails, in consequence introducing substantial bias in control gene selection.

Two other popular scoring approaches, UCell (Andreatta and Carmona 2021), which is an extension of AUCell (Aibar et al. 2017), and JASMINE (Noureen et al. 2022), use rank statistics to increase the stability of results in case of strong technical variation and batch effects. UCell is solely based on Mann–Whitney *U* statistics (Andreatta and Carmona 2021) and is expected to be robust against technical variation as it only uses per-cell gene rank information. JASMINE computes scores by averaging the mean of signature gene ranks and an enrichment value, which corresponds to an odds ratios or likelihood (Noureen et al. 2022). These rank-based scoring methods, however, ignore absolute expression levels, potentially masking meaningful quantitative differences between cells, particularly for highly expressed genes.

Although Noureen et al. (2022) investigated the robustness of various signature scoring methods for bulk and single-cell RNA-seq, focusing on sensitivity and specificity across in silico data sets, the study did not address score range comparability, which is necessary for accurate score-based cell labeling. In contrast, Wang and Thakar (2024) provided a broad comparison of several methods, including UCell, AUCell, JASMINE, and Seurat scoring, analyzing their performance under different factors, such as cell count, gene set size, noise, condition-specific genes, and zero imputation. However, their study did not include cancer data sets or evaluate these methods for score-based cell labeling. Therefore, a complementary benchmark is needed to interrogate the robustness of gene signature scoring methods under the variation in sample cell composition and batch effects, ensuring their reliability and utility for score-based annotation of cell types and states in downstream analysis.

In this work, we extend previously published benchmarks of scRNA-seq scoring methods in three specific aspects: First, we explicitly address the issue of score range comparability, which influences score-based cell labeling; second, we include cancer data sets, in which high cellular and transcriptional heterogeneity introduce additional confounding factors absent in healthy or in silico data; and third, we evaluate the reliability of scores for cell-type and cell-state labeling. We benchmark and analyze the stability of gene signature scores provided by the most popular cell scoring methods: SCANPY, Seurat, UCell, and JASMINE. Using nine human healthy and cancer scRNA-seq data sets, we find that accurate unsupervised cell-state annotation using gene signature scores is not possible based on scores calculated by the benchmarked methods, primarily owing to biased score ranges returned by the methods. To overcome this limitation, we present an improvement of Tirosh's scoring method, Adjusted Neighborhood Scoring (ANS), in which control genes are selected for each signature gene separately to match the gene's expression level across cells. Using several benchmarks, we show that ANS is more robust to batch effects, providing overall the best accuracy in score-based state labeling.

## Results

### ANS method

We propose an improvement to the widely used gene signature scoring method originally introduced by Tirosh et al. (2016), which had been implemented in Seurat (Satija et al. 2015) and, with minor modifications, in SCANPY (Wolf et al. 2018). In brief, the Tirosh method computes gene signature scores for each cell by subtracting a control expression from the signature genes’ expressions. The major drawback of this approach comes from the control gene selection, which uses fixed binning of genes according to their average gene expression across cells (default: 25 bins) (Supplemental Fig. S1). As verified by our benchmarking, such selection may bias the estimates of expression gain, specifically for highly expressed genes, which are frequently used in gene signatures. Our proposed method, ANS, solves this issue by creating control genes for each signature gene separately, by selecting genes in an average-expression neighborhood closest to the signature genes’ average expression (Methods).

### Design of the benchmark

**Figure 1.**
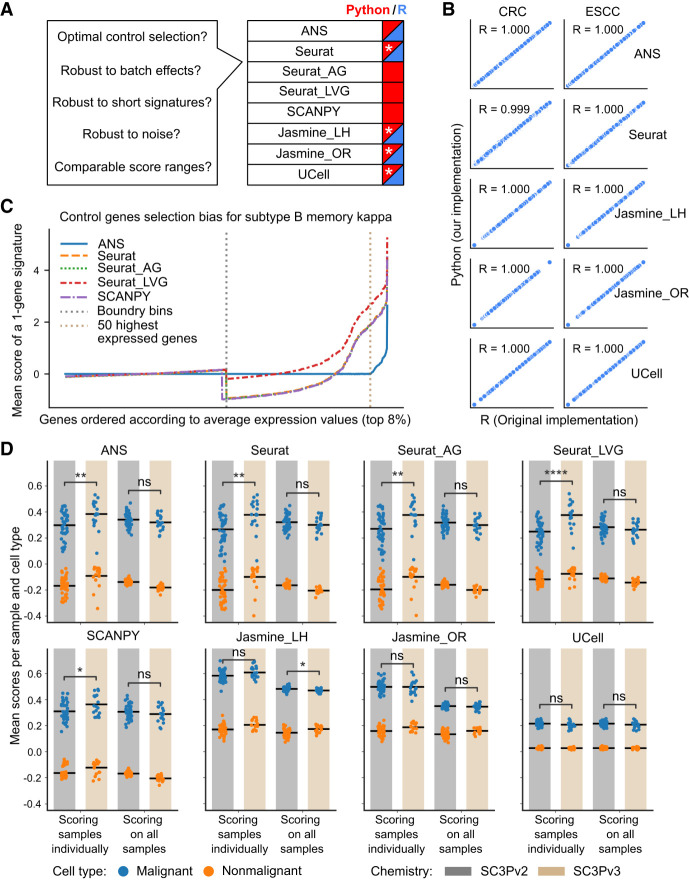
Performance and robustness analysis of gene signature scoring methods. (*A*) Benchmark questions for the gene signature scoring methods and availability of the methods in Python (blue) and R (red). Reimplementations of the original R packages in Python are indicated with a white asterisk. (*B*) The scores obtained from R and Python implementations compared for two cancer data sets (CRC, ESCC). Each subplot shows the Pearson correlation coefficient (R) between implementations. Discrepancies in Seurat scores between R (“AddModuleScore” of Seurat) and Python are attributed to randomization in the scoring method. (*C*) Control gene selection bias of the scoring methods for the top 8% of highly expressed genes in B memory kappa cells within the PBMC data set. The *x*-axis shows genes sorted by their average expression levels; the *y*-axis shows the mean score across all cells for a single-gene signature. Vertical dashed lines indicate the expression bin boundary and the top 50 highly expressed genes. The bias of a scoring method is indicated by how far the mean score of a gene deviates from zero. (*D*) The influence of data set composition and batch effect on scoring CRC cells using a 100-gene signature associated with malignant cells. Each dot represents the mean score for all cells within a sample, grouped by cell type (malignant in blue or nonmalignant in orange), sequencing chemistry type (SC3Pv2 in gray or SC3Pv3 in beige), and scoring mode (scoring all the samples together or individually). The black horizontal bar represents the mean value of all dots within each group. *P*-value annotation: (ns) *P* > 0.05, (*) 0.01 < *P* ≤ 0.05, (**) 1 × 10^−3^ < *p* ≤ 0.01, (***) 1 × 10^−4^ < *p* ≤ 1 × 10^−3^, (****) *p* ≤ 1 × 10^−4^.

In our work, we compared eight gene signature scoring methods (Table 1), including ANS, the original Tirosh method (as implemented in Seurat [Satija et al. 2015] and SCANPY [Wolf et al. 2018]), its variants using all genes (Seurat_AG) or least variable genes (Seurat_LVG) as controls, JASMINE (Jasmine_LH and Jasmine_OR settings) (Noureen et al. 2022), and UCell (Andreatta and Carmona 2021). Like Seurat and SCANPY, Seurat_AG and Seurat_LVG first compute the average expression of the genes, sort them in increasing order, and divide them into 25 equally sized expression bins. Although Seurat_AG selects all genes from an expression bin as control genes, Seurat_LVG chooses genes with the smallest dispersion. For a unified, Python-based benchmark, all methods developed in R (Seurat, JASMINE, UCell) were reimplemented in Python (Fig. 1A). We confirmed the consistency between the R and Python versions of ANS (Methods) (Fig. 1B; Supplemental Table S1), ensuring reproducible and reliable benchmarking.

**Table 1.** Qualitative comparison of signature scoring methods based on their properties, influencing factors, and score comparability

Scoring method	Deterministic	Optimal control gene selection	Independent of total cell number and cell-type proportions	Robust to batch effect when scoring all samples together	Robust when scoring for small signatures	Robust to unrelated genes in the signature	Provides comparable scores for multiple cell-type signatures
Adjusted Neighborhood Scoring (ANS)	✓	✓	✗	✓	✓	✓	✓
SCANPY (Wolf et al. 2018): based on work by Tirosh et al. (2016)	✗	✗	✗	✓	✓	✓	✗
Seurat (Satija et al. 2015): based on work by Tirosh et al. (2016)	✗	✗	✗	✓	✓	✓	✗
Seurat_AG	✓	✗	✗	✓	✓	✓	✗
Seurat_LVG	✓	✗	✗	✓	✓	✗	✗
Jasmine_LH (Noureen et al. 2022)	✓	NA	✗	✓	✓	✓	✗
Jasmine_OR (Noureen et al. 2022)	✓	NA	✗	✓	✗	✓	✗
UCell (Andreatta and Carmona 2021)	✓	NA	✓	✓	✓	✓	✗

A check mark indicates a scoring method fulfills property or resists an influencing factor; a cross mark, the opposite. (Seurat_AG) Seurat scoring with all genes as control; (Seurat_LVG) Seurat scoring with least variable genes as control; (Jasmine_LH) JASMINE with likelihood computation; (Jasmine_OR) JASMINE with odds-ratio computation; (NA) a property or an influencing factor that cannot be evaluated for a scoring method. These results are discussed in detail in the Results section.

We benchmarked the methods using varied scRNA-seq data sets, including peripheral blood mononuclear cells (PBMCs) (Hao et al. 2021), colorectal carcinoma (CRC) (Pelka et al. 2021) esophageal squamous cell carcinoma (ESCC) (Zhang et al. 2021a), breast carcinoma (BRCA) (Wu et al. 2021), high-grade serous ovarian cancer (HGSOC) (Vázquez-García et al. 2022), cutaneous squamous cell carcinoma (cSCC) (Ji et al. 2020), and lung adenocarcinoma (LUAD) (Kim et al. 2020), as well as a neuronal differentiation data set (Jerber et al. 2021). Further details on the benchmark design are provided in the Supplemental Methods.

### Control gene selection bias in gene signature scoring

To evaluate the impact of control gene selection, we compared the approaches used in ANS, Seurat, Seurat_AG, and Seurat_LVG across diverse cell subtypes within the PBMC data set. Specifically, we evaluated the behavior of the scores for the top 8% genes (about 900 genes) with the highest average expression values corresponding to the last two bins of the Tirosh-based methods (Methods) (Fig. 1C; Supplemental Fig. S2). On homogeneous data sets, scores of single-gene signatures are expected to be distributed around zero; therefore, a deviation of the score from zero indicates higher control-gene selection bias. Although we observed minimal score biases in the last but one expression bin, the last bin showed large biases in the score values for all published methods owing to the significant variance of the genes’ average expressions (Fig. 1C). Whereas Seurat, Seurat_AG, and SCANPY first underestimated two-thirds of the gene scores and overestimated one-third, Seurat_LVG overestimated almost all scores in the last expression bin (Fig. 1C). Importantly, all methods showed notable score biases for the top 50 expressed genes; these genes get typically excluded from gene signatures scored by ANS owing to the impossibility of constructing a valid control gene set. Overall, ANS induced the smallest bias, confirming the proposed strategy for control gene selection as the optimal one.

### The influence of cell-type proportions, batch effects, signature length, and inclusion of irrelevant genes on gene signature scoring

The mean control values and, thus, the choice of control genes depend on the data set composition of the tissue or sample analyzed. Consequently, when utilizing methods that employ control genes, we anticipated greater score variability when scoring was conducted on a per-sample basis compared with assessing all cells collectively.

To verify this hypothesis, we calculated scores of 100-gene signatures associated with the malignant cell phenotype of CRC and ESCC data sets (Methods). We compared the distributions of the mean scores per sample and cell type under two scenarios: when scoring was conducted on a per-sample basis and when all samples are together (Fig. 1D; Supplemental Fig. S3). We observed a reduction of score variance for all scoring methods and both CRC and ESCC data sets, except for Seurat_LVG and UCell in the ESCC data set, when scoring was performed on cells of all the samples together (Supplemental Fig. S4), leading to more comparable score ranges over all samples. Additionally, we assessed how scoring on all samples together contributed to diminishing the batch effects for the Tirosh-based scoring methods. For this, we evaluated the differences in score distributions across two batches representing different sequencing chemistry, called SC3Pv2 and SC3Pv3 (Fig. 1D; Supplemental Fig. S5; Supplemental Table S2). Batch effects significantly affected score values of all Tirosh-based scoring methods when the scoring procedure was performed on a per-sample basis. In contrast, we observed decreased distribution shifts between chemistry-related batches when scoring on all samples. Therefore, our results suggest that scoring should be performed on all cells in the data set simultaneously for all methods that use control gene sets, including ANS, as this strategy mitigates both biological and technical batch effects while ensuring comparable score ranges across samples.

Next, we assessed scoring methods’ robustness to changes in the signature length using the CRC and ESCC data sets. Methods were compared based on the minimum number of signature genes needed to perfectly classify malignant and nonmalignant cells (Methods) (Supplemental Figs. S3, S6). Most methods, including ANS, achieved perfect classification with only 12–15 marker genes, selected as top differentially expressed genes between malignant and nonmalignant cells (Supplemental Figs. S3, S6). Jasmine_OR required about 11 times more genes for an accurate cell-type annotation in CRC. The small length of these effective signatures might be owing to the relative ease of distinguishing malignant from nonmalignant cells, that is, that the signatures contain genes with high specificity for the two populations.

Further, to test the methods’ robustness to the presence of irrelevant genes in a signature, we progressively replaced genes in a “pure” 100-gene malignant signature with random genes (Methods). All methods, including ANS and except Seurat_LVG, maintained high performance (area under the ROC curve [AUCROC] > 0.9) with up to 85% noise, requiring only about 15 informative genes in both data sets tested (Supplemental Figs. S3, S6; Supplemental Tables S3, S4). In this case, ANS reached on average 0.88 accuracy, showing the first- and third-best performance for CRC and ESCC, respectively, whereas Seurat_LVG consistently underperformed, needing at least twice as many correct genes for comparable results in both data sets.

We also evaluated computational time requirements of the eight methods as a function of both the number of cells and the number of genes in the signature (Supplemental Fig. S7). All methods exhibited linear scalability with data set size. For short signatures (up to 10 genes), Seurat, SCANPY, and ANS were the fastest (<10 sec for 150,000 cells), whereas Seurat and SCANPY remained the quickest for longer signatures (1000 genes). In contrast, UCell and JASMINE required approximately seven times longer than Seurat and SCANPY, whereas ANS was about three times slower in this case. To evaluate the practical impact of the increased computation time of ANS, we assessed the length distribution of 35,134 gene signatures from the MSigDB human collection (Supplemental Fig. S8; Liberzon et al. 2015). The majority (64%) of analyzed signatures had lengths within the range of 100 genes, indicating that in practice ANS offers efficient computation, within seconds to a few minutes depending on the data set size, while providing increased performance.

### Assessing score-range comparability for score-based cell-type and cell-state annotation

Reliable score-based annotation requires scores with high information content and comparable ranges. To assess the information quantity and score comparability produced by ANS and other methods, we analyzed the accuracy of cell-type and cell-state annotation across four cancer data sets (BRCA, HGSOC, LUAD, and sCC) and four PBMC cell subsets, using specific nonoverlapping gene signatures (Methods) (Supplemental Table S5). We accessed the fairness of produced scores by evaluating the accuracy of an unsupervised cell annotation, based on the argmax assignment of cells to states from signature scores, against the ground-truth cell labels. Additionally, we evaluated the information quantity of scores produced by each scoring method using a cross-validated supervised logistic regression predicting cell state based on signature scores (Methods).

**Figure 2.**
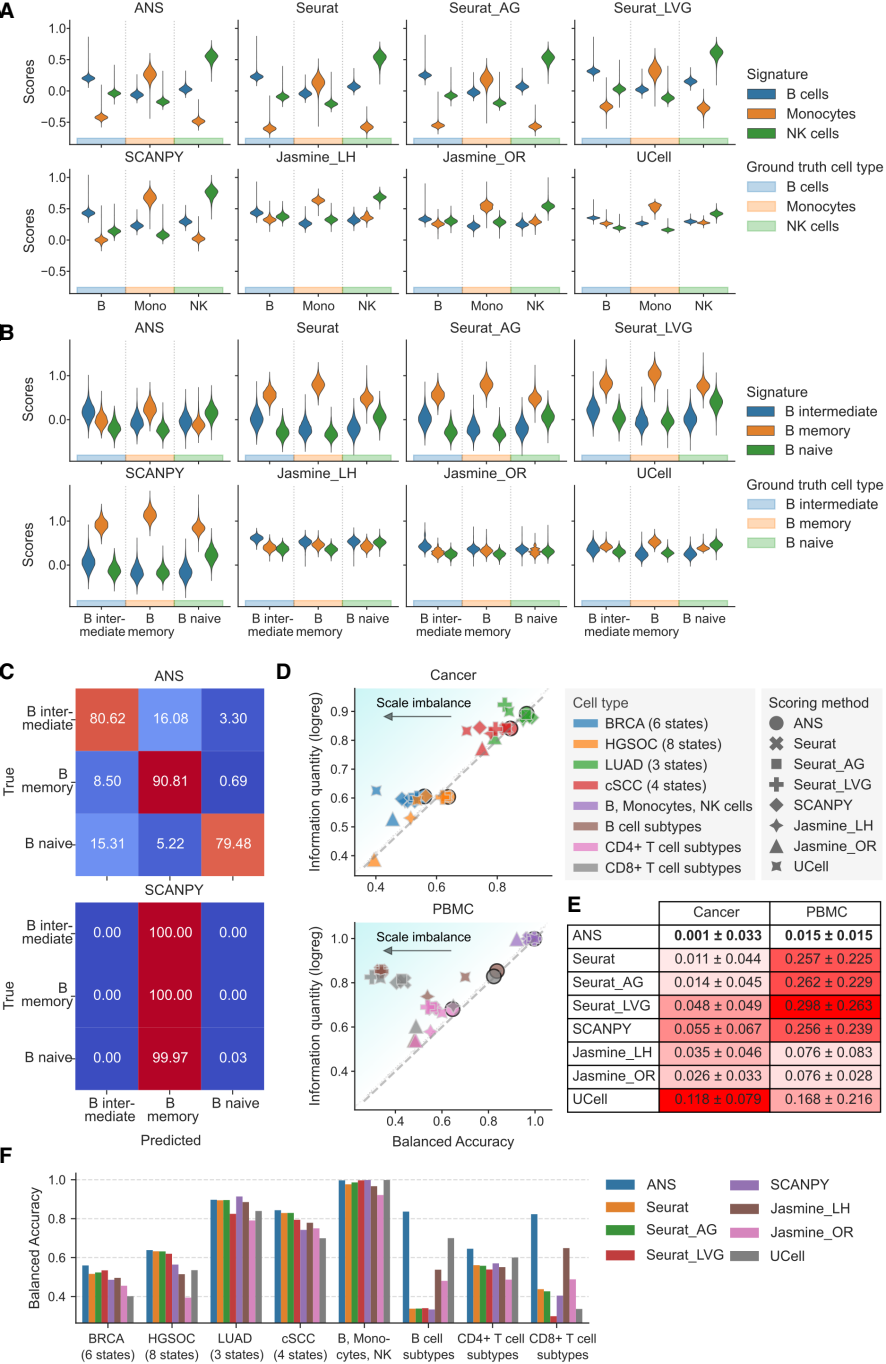
Comparative analysis of scoring methods for cell-type and cell-state annotation. Only nonoverlapping cell-type- or cell-state-specific signatures were used. (*A*) Score distributions for cell-type-specific signatures (B cells, monocytes, and NK cells) separated by true cell-type annotations, calculated for each scoring method. (*B*) Score distributions for B cell subtype signatures separated by true cell subtypes, calculated by each scoring method. (*C*) Row-normalized confusion matrix of B cell subtype annotation based on the highest scores. (*D*) Relationship between hard-labeling performance and score information quantity in cancer and PBMC data sets. Scatterplots show balanced accuracy (*x*-axis) against score information quantity (*y*-axis) for various scoring method–data set combinations. Balanced accuracy quantifies hard-labeling performance; a score information quantity indicates the scores’ effectiveness in subtype classification. The diagonal line indicates perfect metric alignment, with vertical distances from this line representing scale imbalance. (*E*) Quantitative analysis of scale imbalance across scoring methods and tissue types (cancer and PBMC). The mean and standard deviation of scale imbalance for each method are shown. Scale imbalance is the absolute difference between score information quantity and balanced accuracy in direct label assignment. The method with the lowest mean scale imbalance, indicating optimal consistency between information content and labeling accuracy, is highlighted in bold. The intensity of the red background corresponds to the increase in scale imbalance. (*F*) Cell-state and cell-type annotation performance overview for all eight data sets and scoring methods.

We observed that score-based cell annotation was accurate for distinguishing cell types (B cells, monocytes, and NK cells) in the PBMC data set using scores produced by most methods (Fig. 2A). All methods achieved high balanced accuracies ranging from 0.921 (Jasmine_OR) to 0.999 (SCANPY and UCell) and F1-scores from 0.958 (Seurat) to 0.999 (UCell), demonstrating robust performance in distinguishing these distinct cell types, with ANS presenting the second-best balanced accuracy and F1-score (0.997 and 0.996, respectively) (Supplemental Table S6). Indeed, most scoring methods produced comparable score ranges, with the highest scores observed for cells of matched cell type; JASMINE was an exception, showing overlapping ranges for signature scores across B cells (Fig. 2A).

However, when we applied the score-based annotation approach to assign cell states—naive, intermediate, and memory B cells—we observed limitations in using scores produced by all methods except ANS. Indeed, ANS was the only approach to produce informative and comparable score ranges for the evaluated cell states (Fig. 2B). Tirosh-based methods (Seurat, Seurat_AG, Seurat_LVG, and SCANPY) consistently scored highest for the B memory cell signature, regardless of the true cell state, resulting in poor labeling performance (Fig. 2C; Supplemental Fig. S9). UCell showed higher B memory cell scores than B intermediate ones when applied to B intermediate cells. Conversely, JASMINE scored highest for the B intermediate cell signature in B intermediate cells but failed to produce distinguishable score ranges for other cell states.

To comprehensively evaluate observed patterns, we expanded our analysis with four data sets of malignant cells with ground-truth assignments of cells to malignant cell states (BRCA, HGSOC, LUAD, and sCC) and two additional PBMC cell subsets comprising CD4 (CD4^+^) and CD8A (CD8^+^) cell-state annotations. Across these six data sets, ANS maintained high performance, providing unbiased gene signature scores for cell annotation, achieving the highest balanced accuracy in five cases (BRCA: 0.560; HGSOC: 0.638; cSCC: 0.843; CD4^+^ T cells: 0.645; CD8^+^ T cells: 0.823) and performing competitively in LUAD (0.897 vs. SCANPY's 0.914) (Fig. 2F; Supplemental Figs. S9, S10; Supplemental Table S6).

Further, to assess score information content, we compared the unsupervised score-based cell classification accuracy with a supervised cross-validated linear classifier approach. We define the latter's accuracy as the information quantity of the gene signature scores, representing the feasibility of accurate cell annotation (Methods). We define the difference between the information quantity and the unsupervised accuracy as the scale imbalance, which indicates score ranges that vary substantially across cell types (Supplemental Methods). Across the eight data sets (seven for cell-state and one for cell-type annotations), ANS consistently ranked among the best-performing methods, showing the closest alignment of balanced accuracy of the unsupervised score-based annotation with information quantity (Fig. 2D). Notably, ANS demonstrated substantially better performance for sCC, CD8^+^, and B cell subtype classification compared with other scoring methods. Moreover, across both cancer and PBMC data sets, ANS exhibited minimal scale imbalance, showing the smallest standard deviation, suggesting comparable score ranges (Fig. 2E; Supplemental Table S6). Although these results are based on nonoverlapping gene signatures, similar outcomes were observed for signatures that shared certain genes (Supplemental Figs. S11, S12; Supplemental Table S7).

### Assessing score-based annotation of rare and transitioning cell populations

**Figure 3.**
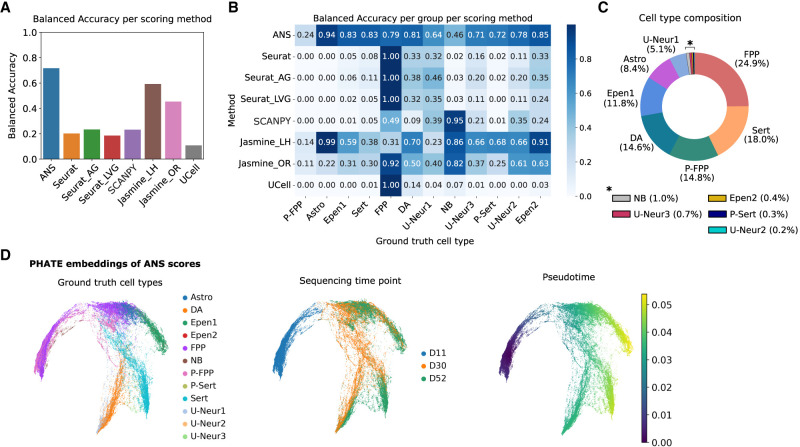
Benchmarking of score-based cell labeling using neuronal differentiation single-cell data set. (*A*) Overall balanced accuracy of score-based labels assigned based on the 12 cell signatures. (*B*) Cell-type-specific balanced accuracy across all benchmarked scoring methods. (*C*) Cell-type proportion distribution across the analyzed data set, including floor plate progenitors (FPP), proliferating floor plate progenitors (P_FPP), neuroblasts (NB), dopaminergic neurons (DA), serotonergic-like neurons (Serts), proliferating serotonergic-like neurons (P_Serts), astrocyte-like (Astro), ependymal-like 1 (Epen1), ependymal-like 2 (Epen2), and three unknown neuron groups 1–3 (U_Neur1, U_Neur2, U_Neur3). (*D*) PHATE embeddings generated based on the ANS scores of the 12 cell types. The plots are colored by ground-truth cell-type annotations, sequencing time points (days), and pseudotime estimated from the embeddings.

To test the performance of scoring methods in a biological context involving state transitions and rare cell populations, we assessed the feasibility of score-based cell labeling using a neuronal differentiation data set from (Jerber et al. 2021). The data set is composed of 215 human induced pluripotent stem cell (iPSC) lines undergoing differentiation toward a midbrain neural fate (Fig. 3; Supplemental Figs. S13–S15). Specifically, single cells were profiled at three maturation stages (days 11, 30, and 52), capturing a continuum of differentiation states from progenitor-like to mature neuronal states, including floor plate progenitors (FPP), proliferating floor plate progenitors (P_FPP), neuroblasts (NB), dopaminergic neurons (DA), serotonergic-like neurons (Serts), proliferating serotonergic-like neurons (P_Serts), astrocyte-like cells (Astro), ependymal-like 1 (Epen1), ependymal-like 2 (Epen2), and three uncharacterized neuronal cell subtypes (U_Neur1–3).

We observed substantial variation in the score ranges across tested cell-state signatures, with most methods, except for ANS and JASMINE, showing consistently high scores for FPP and NB signatures regardless of the underlying cell type (Supplemental Fig. S13). ANS achieved overall the highest balanced accuracy of score-based annotation across all 12 cell types, with other Tirosh-based approaches showing substantially worse performance (Fig. 3A). Of note, although the data set contains several rare cell populations, including NB, P_Serts, Epen1, and U_Neur1–2, each representing only 0.2% to 1.3% of all cells, ANS-based annotation was the best for three out of five of those cell types and second best for the remaining two (Fig. 3B,C). Moreover, ANS-based annotations showed the highest pairwise balanced accuracy for closely related populations (Epen1 and Epen2, FPP and P_FPP, DA and U_Neur1, and Sert and U_Neur3) (Fig. 3B; Supplemental Fig. S14), highlighting its ability to discriminate transcriptionally similar cells.

We further explored ANS-derived scores to verify if they accurately capture continuous cellular transitions. Specifically, we used potential of heat diffusion for affinity-based transition embedding (PHATE) (Moon et al. 2019), a dimensionality reduction method that preserves both local and global structure in the data, to project analyzed cells into a low-dimensional manifold. The resulting ANS score–based embeddings accurately reconstructed the expected differentiation trajectories and were consistent with both the ground-truth cell-type labels and sequencing time points (Fig. 3D). Importantly, ANS score–based embeddings presented clearer lineages compared with embeddings derived from raw gene expression data (Supplemental Fig. S15). Although these results further illustrate the practical utility of ANS for capturing meaningful biological signals, a systematic comparison with other dimensionality reduction approaches warrants further investigation. Nevertheless, ANS scores effectively denoise the signal and provide robust features for trajectory inference of cells in continuous states.

### Assessing the effect of removing highly expressed signature genes for score-based cell annotation

As described in the previous section, all Tirosh-based approaches suffer from bias when control genes are selected for highly expressed signature genes. To mitigate this issue, ANS filters out signature genes that fall within the top *c*/2 expressed genes, where *c* is the size of the control gene set. To directly evaluate the benefit of our control gene selection strategy, we performed additional benchmarking experiments on five PBMC subsets, including both broad cell types (B cells, NK cells, and monocytes) and finer cell states (B cell, CD4^+^ T cell, and CD8^+^ T cell subtypes), under five different signature gene filtering strategies. Specifically, we applied (1) no filtering; (2) removal of the top 50 and (3) top 100 expressed genes, corresponding to ANS-based filtering with control sets equal to 100 or 200 genes, respectively; and (4) removal of genes in the top expression bin when genes were split into 25 bins or (5) into 50 bins. We compared the balanced accuracy of score-based cell assignments using ANS and the four Tirosh-based approaches across all filtering settings.

Although all methods achieved almost perfect accuracy for broad cell-type identification (i.e., B cells, NK cells, and monocytes) across the filtering strategies, we observed considerable differences for cell-state annotation, with ANS consistently achieving the highest balanced accuracy (Supplemental Fig. S16). Tirosh-based methods reached comparable performance only after removing signature genes within the top 100 expressed genes, but their accuracy declined again when filtering by top expression bin, highlighting their sensitivity to the selected filtering threshold.

Notably, our analysis showed that a large proportion of signature genes often falls within the top expression bins (Supplemental Fig. S17; Supplemental Tables S8–S12), suggesting that bin-based filtering may eliminate too many biologically informative genes and thereby reduce the robustness of score-based cell annotation. In contrast, ANS-based annotation reached optimal accuracy when excluding only the 50 most highly expressed genes and maintained high accuracy even without gene filtering, directly demonstrating the benefits of the adaptive control gene selection. Together, these results confirm that our proposed control gene selection strategy enhances score robustness for cell annotation beyond what can be achieved through simple filtering of highly expressed signature genes.

### Devising cancer cell–specific EMT signature: a case study for the ANS application

To showcase the practical utility of ANS, we used it to infer a pancancer malignant cell-specific EMT signature. The difficulty in constructing such a gene signature comes from the high similarity of transcriptional states of malignant EMT-exhibiting cells with malignant non-EMT cells, on the one hand, and with nonmalignant mesenchymal cells, such as cancer-associated fibroblasts (CAFs), on the other hand.

**Figure 4.**
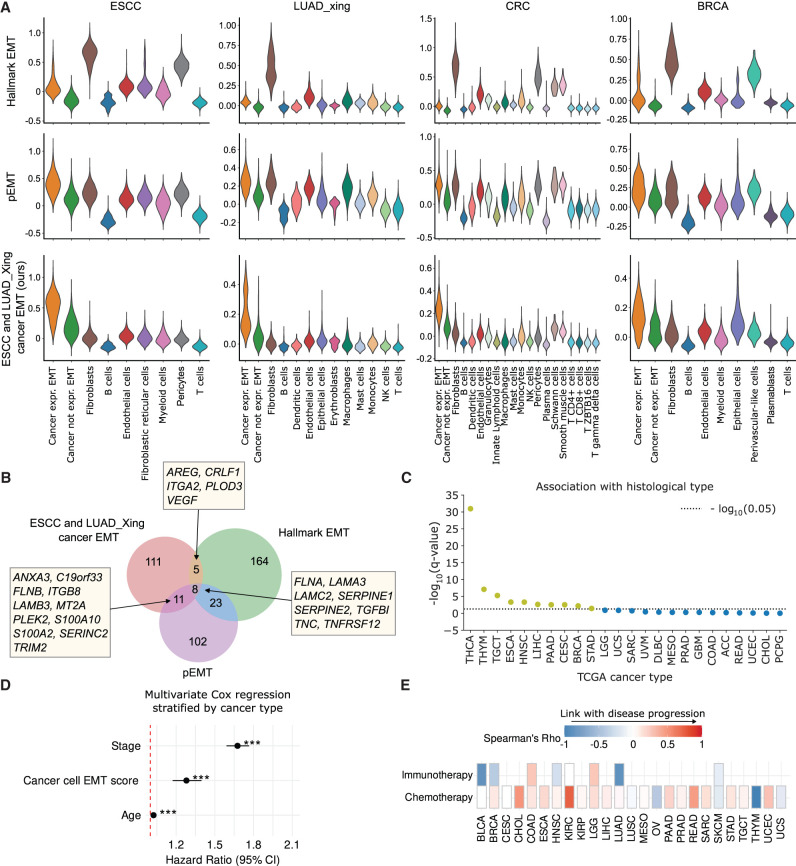
Application of ANS to devise EMT signature specific to malignant cells. (*A*) Score distributions of the Hallmark EMT, the pEMT, and our proposed ESCC-specific and LUAD_Xing-specific cancer EMT gene signatures in different cell types of ESCC, LUAD_Xing, CRC, and BRCA. (*B*) Venn diagram for the overlap in gene lists for the Hallmark EMT, pEMT signatures, and the signature we designed. (*C*) Association of ESCC-specific and LUAD_Xing-specific cancer EMT signature scores and histological subtypes in TCGA (only cancer types with at least one histotype are included). Significant associations (*Q* value < 0.05) are represented by dots *above* the dotted line (yellow). The FDR-adjusted *P*-values from the two-sided Kruskal–Wallis test were −log_10_-transformed and sorted in decreasing value, with a higher *y*-value indicating higher significance. (*D*) Multivariate Cox survival analysis with disease stage, patient age, and cancer cell–specific EMT signature scores calculated in bulk TCGA RNA-seq data. The model was stratified by cancer type. Variable-specific *P*-value significance, (***) *P*-value < 0.001, (**) *P*-value < 0.01, (*) *P*-value < 0.05. (*E*) Association between the cancer cell–specific EMT scores in bulk TCGA tumors and patient treatment response. Association was calculated with Spearman's rank correlation between the scores and treatment response ranked one (complete response) to four (clinical progressive disease; Methods).

Indeed, when we explored the behavior of previously published EMT signatures using the ANS scoring method on single cells from the ESCC (Zhang et al. 2021a), lung adenocarcinoma by Xing et al. (2021) (LUAD_Xing), and CRC (Pelka et al. 2021) and BRCA (Wu et al. 2021) tumors, we observed that the published pancancer EMT signatures, such as the Hallmark EMT (Subramanian et al. 2005; Liberzon et al. 2015), the pEMT signature (Barkley et al. 2022), and five other EMT signatures (Gröger et al. 2012; Tan et al. 2014; Mak et al. 2016; Foroutan et al. 2017; Hollern et al. 2018), resulted in extremely high scores in CAFs in addition to the mesenchymal-like (MES-like) malignant cells (Fig. 4A; Supplemental Fig. S18), whereas the signatures of Foroutan et al. (2017), Gröger et al. (2012), Mak et al. (2016), and Tan et al. (2014) resulted in high scores for pericytes in CRC and ESCC, and the former also scored high in endothelial cells for all data sets (Supplemental Fig. S18).

To devise a cancer cell–specific EMT signature, we scored established EMT signatures using ANS in the ESCC and LUAD_Xing data sets. Briefly, we selected genes that were significantly upregulated in cancer cells with the highest EMT scores (classified as cancer EMT cells) compared with CAFs in the two data sets (Methods). Overall, the signature included many known EMT-related genes, as well as genes previously not included in canonical EMT signatures (Fig. 4B; Supplemental Table S13). By construction, the devised signature scored highly for the MES-like malignant cells in the training data sets (ESCA and LUAD_Xing), and clearly distinguished cells in the validation data sets (CRC and BRCA) (Fig. 4A; Supplemental Table S14). Specifically, the accuracy of our gene signature discriminating MES-like malignant cells versus all remaining cells measured with the area under the precision-recall curve (AUCPRC) on CRC and BRCA data sets was 0.701 and 0.511, respectively; it was slightly outperformed on CRC only by the gene signature proposed by Hollern et al. (2018) (AUCPRC 0.707) (Supplemental Table S14). However, this signature performed suboptimally in our ESCC and LUAD_Xing training data sets (AUCPRC 0.028 vs. 0.592 of our gene signature in ESCC and 0.244 vs. 0.513 in LUAD_Xing).

Calculation of cancer cell–specific EMT phenotypes using bulk RNA-seq data offers a promising alternative to assess their link with clinical characteristics in cases when corresponding single-cell data are not available. To verify the applicability of our devised signature in bulk tumors, we used RNA-seq from The Cancer Genome Atlas (TCGA) data sets and scored both our cancer-cell EMT and hallmark EMT signatures.

Scores of the ANS-derived signature showed higher correlation with tumor purity than the hallmark EMT ones in 29/33 cancer types (Supplemental Fig. S19), indicating higher sensitivity of our signature to capture EMT signals within the malignant compartment than with the nonmalignant one. Next, we compared the score distributions across cancer types (Supplemental Fig. S20). Hematological malignancies, including acute myeloid leukemia (LAML) and diffuse large B cell lymphoma (DLBC), showed the lowest scores, consistent with their nonepithelial origin and the absence of EMT in liquid cancers. In contrast, epithelial cancers, such as head and neck squamous cell carcinoma (HNSC), pancreatic adenocarcinoma (PAAD), and cervical squamous cell carcinoma and endocervical adenocarcinoma (CESC) showed the highest scores, aligning with the well-established involvement of EMT in these tumor types (Lee et al. 2008; Puram et al. 2017; Yang et al. 2023). The Hallmark EMT signature showed much higher scores in sarcomas (SARC) than our cancer cell–specific EMT signature, an observation consistent with the mesenchymal rather than epithelial origin of this tumor type. The elevated Hallmark EMT scores in SARC therefore likely reflect the inclusion of stromal and mesenchymal gene expression in bulk tumor samples, further supporting high specificity of our cancer cell–specific EMT signature. Finally, we observed significantly higher cancer-cell EMT scores in primary melanoma tumors (*P*-value < 0.0001) (Supplemental Fig. S21), an observation consistent with the hypothesis that EMT is necessary to gain motility for invasion, whereas mesenchymal-to-epithelial transition (MET) is necessary for new site establishment (Tsai et al. 2012). In the same data set, hallmark EMT scores showed no significant differences, further highlighting higher specificity of our proposed signature (Supplemental Fig. S21).

Next, we used TCGA bulk RNA-seq data to investigate clinical significance of the proposed cancer cell–specific EMT signature. We found our signature scores significantly associated with the histological subtype in 10/24 cancer data sets for which at least two subtypes were available (Fig. 4C; Supplemental Table S15). The scores were significantly associated with worse patient outcome in multivariate Cox regression analysis stratified by cancer type, with patient age and tumor stage as covariates (*P*-value < 0.05) (Fig. 4D). The association remained significant also in a model including hallmark EMT scores, providing evidence that our devised signature captures additional clinically relevant signal beyond that of the hallmark EMT signature (Supplemental Fig. S22). Finally, we assessed the association between patient treatment response and the calculated cancer cell–specific EMT scores to test the hypothesis that EMT-like cancer cells within tumors lead to higher treatment resistance (Shibue and Weinberg 2017; Brabletz et al. 2018). We found positive association between the scores and worse treatment response of patients treated with chemotherapy in 15 of 25 cancer types, as well as in two of eight cancer types for which patients were treated with immunotherapy (Fig. 4E). Together, these results highlight the biological significance of the proposed signature and further demonstrate potential of ANS in revealing biologically and clinically relevant information.

## Discussion

In this study, we systematically evaluated scRNA-seq gene signature scoring methods for unsupervised cell annotation, an approach widely used in single-cell studies to characterize cellular populations. In the context of cancer research, in which transcriptional programs and diverse cellular states are strongly associated with patient survival and treatment resistance, accurate cell annotation is essential for identifying clinically relevant populations. Our analyses across nine healthy and cancer scRNA-seq data sets, covering diverse gene signatures and experimental conditions, revealed that established scoring methods produce scores with mismatched ranges, making them unreliable for cell annotation based on maximal signature scores.

To address this issue, we developed a robust gene signature scoring method, ANS, which refines the approach introduced by Tirosh et al. (2016) by deterministically selecting optimal control gene sets to normalize expression values for technical effects and to minimize bias in the resulting scores. Our benchmarking highlighted substantial limitations of established scoring methods for cell-state assignment, showing that only ANS maintained high accuracy across diverse contexts, including healthy, cancer, and neuronal differentiation data sets, for this task.

Our analysis showed that the selection of control genes and the resulting score distributions depend on the composition of the input data set. Specifically, when scoring was performed on a per-sample basis, selection of the control genes was biased by sample-specific effects, leading to higher variability in gene signature scores across samples. We demonstrated that this issue can be solved by scoring all cells simultaneously using all the samples in the data set. Importantly, we showed that such an approach can mitigate technical batch effects present in data sets, effectively allowing users to omit the challenging step of data integration. As this aspect has not been previously addressed, our results highlight an important practical consideration for future single-cell scoring analyses.

Moreover, we demonstrated the robustness and stability of ANS against noisy signatures. All benchmarked methods were stable when scoring small and noisy signatures (with up to 85% random genes), with ANS among the top three methods. Moreover, ANS was the top-performing approach for providing high score information quantity and comparable score ranges. Although other Tirosh-based methods showed high information quantity, they suffered from large-scale imbalances, likely owing to biased control gene selection and lack of score normalization.

It is important to emphasize that gene signature scoring is only as reliable as the provided signatures. This is especially important when using signature scores for cell labeling, as an unreliable signature can result in misclassification. Therefore, in our benchmark we derived gene signatures directly from the data sets in which they were evaluated or used the signatures identified in the original studies of the data sets used. Nevertheless, the limited specificity of some of these signatures led to overall poor annotation performance, particularly for BRCA, HGSOC, and CD4^+^ cells (Fig. 2D).

Importantly, although the control set selection bias can be partially mitigated by the removal of signature genes highly expressed in the data set, our analysis showed that even without filtering of the genes, ANS provides the best performance, robust against different filtering strategies, whereas other Tirosh-based methods are more dependent on the filtering thresholds. ANS therefore fulfills this need with a reliable control gene selection strategy, ensuring greater accuracy compared with other tested methods.

Further, we showcased the use of the ANS scoring method to derive a gene signature specific to EMT-like malignant cells. Building a cancer cell–specific signature for EMT could allow scoring bulk RNA-seq tumor data sets to quantify the degree of EMT transformation in human tumors while minimizing the bias induced by the presence of nonmalignant cells of mesenchymal origin in the tumors. Our ESCC- and LUAD_Xing-specific cancer EMT signature outperformed others in distinguishing MES-like malignant cells from CAFs across all data sets. In addition, it included several genes, which so far have not been considered in any of the used pancancer EMT signatures, for instance, *PITX1*, a transcriptional factor, which dysregulation has been associated with tumor progression in LUAD (Song et al. 2018), kidney renal clear cell carcinoma (Zhang et al. 2021b), breast (Wang et al. 2020), epithelial ovarian (Li et al. 2021), prostate cancer (Poos et al. 2022), melanoma (Ohira et al. 2021), ESCC (Otsubo et al. 2017), osteosarcoma (Zhang et al. 2023) and head and neck squamous cell carcinoma (Jin and Qin 2020). Other EMT-related genes exclusively present in our signature included two long-noncoding RNA genes: *BICDL3P* (previous gene symbol *ABHD11-AS1*), suggested as a prognostic biomarker for pancreatic cancer (Qiao et al. 2018), and *BCYRN1*, shown to promote cell migration and invasion in lung and colorectal cancers (Hu and Lu 2015; Gu et al. 2018; Yu and Chen 2019; Song et al. 2022). Additionally, the protein-coding genes *FAM83A*, *ITGA3*, *ITGB4*, *L1CAM*, *MUC16*, and *SAA1* have been reported to promote cancer progression (Chen et al. 2018; Cheriyamundath and Ben-Ze'ev 2020; Lei et al. 2020; Li et al. 2020; Tian et al. 2020; Zhai et al. 2020; Zhang et al. 2020; Zheng et al. 2020; Ji et al. 2021; Schinke et al. 2022). Lastly, our analysis has revealed that the identified cancer cell–specific EMT signature corresponds to histological subtypes in several TCGA data sets, predicts worse patient survival, and is associated with treatment resistance. Beyond identifying clinically relevant EMT signatures, we showed that ANS can accurately assign transcriptionally similar and transitioning cells, such as those undergoing neuronal differentiation. ANS scores can serve as an informative low-dimensional representation of cellular states, which can be further used for the reconstruction of cellular trajectories.

We acknowledge the limitations inherent in our study. First, because of the exclusive assignment of each cell to a specific cell type or state and the lack of intermediate labels for cells transitioning between states, such as from naive B cells to memory B cells, our benchmarking of cell annotation was based on the assumption of exclusive gene signature activation within individual cells and the associated hard labeling of cells. To consider cells transitioning between states, a user could use a baseline score of 0 as the threshold for gene signature enrichment in a cell. An alternative approach for such scenarios could involve associating gene signature scores with cell trajectories. Although our analysis in the neuronal differentiation data set supports the feasibility of this approach, further experiments are necessary for comprehensive validation. Nevertheless, our primary objective in this work was to assess the scoring specifically for cell annotation; therefore, we focused on evaluating hard-labeled cells as the most straightforward approach.

Second, we note that ANS may be less accurate for detection of very similar cell types compared with more distinct cell types, as in the case of P_FPP, FPP, and NB cells in the neuronal differentiation data set. Nevertheless, our benchmark included data sets with varied composition, including rare cell types (proportion <0.1%) and sparsity reaching 90% (Supplemental Figs. S23–S25), with our results demonstrating superior performance of ANS in these data sets, providing evidence for its high robustness even in challenging scenarios.

Third, given that the marker genes of cellular phenotypes are often among the top highly expressed genes, selection of appropriate control genes for such signature genes might be impossible. ANS solves this problem by removing the number of the top expressed genes equal to half the size of the user-defined control set. In extreme cases in which either signature is very short or the selected control size is very big, all the signature genes may be excluded. In such cases, we recommend adjusting the size of either the signature or the control sets.

Finally, in our analysis to identify cancer cell–specific EMT signature, we initially relied on the selection of the cancer cells expressing high scores of previously published EMT-related gene sets. Our applied selection procedure of EMT-expressing cancer cells might have biased the downstream analysis. Extending the analysis with additional data sets in which cancer cells with mesenchymal-like properties have been identified would likely increase robustness of our proposed signature.

Nevertheless, our work presents a systematic evaluation of single-cell scoring methods, revealing the limitations of existing approaches for unsupervised cell annotation and introducing ANS as a novel method that outperforms current techniques in this task. Given the widespread use of score-based cell-type and cell-state labeling in single-cell studies, ensuring the accuracy and reliability of such annotations is essential for downstream analyses and ANS directly addresses this need.

## Methods

### Data sets

We used nine different scRNA-seq data sets, comprising seven cancers, one neural differentiation, and one PBMC data set (Hao et al. 2021). For control gene selection and robustness experiments, we utilized a ESCC (54 samples) (Zhang et al. 2021a) and a CRC (60 samples) (Pelka et al. 2021) data set. To assess signature score range comparability, we considered malignant cells from four cancer data sets: BRCA (20 samples) (Wu et al. 2021), HGSOC (134 samples) (Vázquez-García et al. 2022), cSCC (seven samples) (Ji et al. 2020), and LUAD (nine samples) (Kim et al. 2020), as well as the PBMC data set (consisting of 24 samples collected at three different time points from eight patients). The preprocessed data sets for CRC, ESCC, LUAD_Xing, BRCA, sCC, HGSOC, LUAD, and PBMC and the used signatures can be downloaded at https://drive.google.com/drive/folders/10L2gqapJbyOn_MbrZRHQG--n0Xj7wIyg. The raw transcript count matrices can be obtained from the NCBI Gene Expression Omnibus (GEO; https://www.ncbi.nlm.nih.gov/geo/) under accession number GSE178341 for CRC, GSE176078 for BRCA, and GSE160269 for ESCC. The raw transcripts for LUAD_Xing can be found under the accession number HRA000154 at http://bigd.big.ac.cn/gsa-human. The raw transcripts for the remaining data sets can be found at GEO: sCC (GSE144240, GSE144236), HGSOC (GSE180661), and LUAD (GSE131907). The raw PBMC data set can be downloaded from https://atlas.fredhutch.org/nygc/multimodal-pbmc/. For score-based annotation benchmark, we used a publicly available preprocessed neural differentiation data set (Jerber et al. 2021), which can be downloaded from Zenodo (https://zenodo.org/records/4333872). The case study included the ESCC and CRC data sets, along with another lung adenocarcinoma data set (LUAD_Xing, 19 samples including 12 from subsolid nodules and seven from primary LUAD) (Xing et al. 2021) and the seven basal-like samples from BRCA. Overall cell-type proportion distribution and data set sparsity are presented in Supplemental Figures S23–S25. Details on the preprocessing of the data sets can be found in the Supplemental Methods (Supplemental Figs. S26–S28).

### Gene signature selection for malignant cells in benchmark

To select gene signatures for malignant cells in CRC and ESCC, we bulkified the data sets per sample using the method get_pseudobulk from the Python package decoupleR (Badia-I-Mompel et al. 2022) with the parameters mode=sum, min_cells=10, and min_counts=1000. As described in the “pseudobulk functional analysis” tutorial of decoupleR (version 1.4.0) (Badia-I-Mompel et al. 2022), we further filtered genes by expression using the default parameters. We then applied the PyDESeq2 (version 0.3.3) (Muzellec et al. 2023) workflow to identify genes with significant differential expression (log_2_ fold change (log_2_FC) > 2 and adjusted *P*-value < 0.01) between the malignant and nonmalignant cells.

### Tirosh-based gene signature scoring and ANS

Methods implemented in the scRNA-seq analysis packages SCANPY (in Python) (Wolf et al. 2018) and Seurat (in R) (Satija et al. 2015) were built upon the procedure first described by Tirosh et al. (2016). Here, we recall this procedure in a formal manner. Let $X{\in R}_{\geq 0}^{n\times p}$ be the preprocessed expression matrix for *n* cells and *p* genes, generally representing log-transformed normalized read counts. Let *S* = {*s*_1_, · · · , *s*_*m*_} be a given gene signature, that is, a set of *m* genes. Let $\bar{g}={\left({\bar{g}}_{i}\right)}_{i=1:p}\in R_{\geq 0}^{p}$ be the average expression vector for each gene *i* over all cells ordered by increasing average expression (i.e., ${\bar{g}}_{i}\leq {\bar{g}}_{i+1}$ for any *i*).

In Tirosh-based gene signature scoring methods, $\bar{g}$ is split into 25 equally sized bins, called expression bins. For each signature gene *s*, we sample *c* (control size) genes from *s*’s expression bin. Let *C*_*s*_ be the set of control genes for the signature gene *s*. The score for a cell *j* is then computed as follows:

$$\mathrm{scor}e_{j}=\frac{1}{m}\sum_{s\in S}\left(X_{\;j,s}-\frac{1}{c}\sum_{k\in C_{s}}X_{\;j,k}\right).$$

In this work, we kept the main idea of the scoring approach proposed by Tirosh et al. but suggested and validated three alternatives to select control genes.

When implementing the first two alternatives, we preserved the gene binning approach. In the “all genes as control genes” alternative (Seurat_AG), we selected all nonsignature genes from an expression bin as control genes. Note that Seurat_AG was not using the control genes size parameter *c*. In the least variable control genes alternative (Seurat_LVG), we first computed the least variable genes for each expression bin. The least variable genes were calculated with the SCANPY highly_variable_genes method (flavor “seurat”) and selected by taking the *c* genes with the smallest dispersion.

In the third alternative, called Adjusted Neighborhood Scoring (ANS), the control genes for a signature gene are selected based on the average expression neighborhood. Let $\tilde{g}$ be equal to $\bar{g}$ but exclude the signature genes *S*. Let $C={\left\{C_{k}\right\}}_{k=1}^{(\;p-m)-c+1}=\{\{{\tilde{g}}_{k},\cdots ,{\tilde{g}}_{k+c}\}|1\leq k\leq (\;p-m-c+1)\}$ be the set of windows of size *c* (control size) of $\tilde{g}$. For each signature gene *s*, we computed the control set as follows:

$$C_{s}=\arg\underset{C_{k}\in C}{\min}\left|{\bar{g}}_{s}-\frac{1}{c}\sum_{{\bar{g}}_{l}\in C_{k}}{\bar{g}}_{l}\right|.$$

In other words, for each signature gene, we selected *c* control genes around this gene whose average mean expression closely matched the mean expression of the signature gene. Of note, ANS excluded signature genes within the $\frac{c}{2}$ genes with the highest average expression to avoid invalid control gene selection.

### Evaluation of scoring methods for score-based cell-type and cell-state annotation

We performed unsupervised cell-type/-state annotation using gene signature scoring in all experiments. For a data set with *n* signatures associated with *n* cell types/states, we assigned cell identity based on the highest scoring signature (argmax). Performance was assessed using scikit-learn (version 1.4.1.) (Pedregosa et al. 2011) metrics: AUCROC for balanced data sets and AUCPRC for unbalanced data sets, along with balanced accuracy and weighted F1-score.

### Scoring methods in R and Python

We implemented the R methods UCell (https://github.com/carmonalab/UCell) and JASMINE (https://github.com/NNoureen/JASMINE), as well as the original method proposed by Tirosh et al. (2016), in Python. The R implementation of the Tirosh et al. method is implemented in the scRNA-seq package Seurat (AddModuleScore) (Hao et al. 2021). We provided the ANS scoring method in both Python and R platforms. To demonstrate cross-platform consistency (Fig. 1B), we used the preprocessed cancer data sets: CRC and ESCC. For each data set, we selected a 100-gene signature for malignant cells based on the genes with the lowest adjusted *P*-value and the highest log_2_FC (cf. the section “Gene signature selection for malignant cells in benchmark” in the Methods) and scored these signatures on the respective data sets on both platforms. We computed the Pearson correlation coefficient between the scores from each platform for each data set using the scipy.stats.pearsonr function from the SciPy package (version 1.12.0) (Virtanen et al. 2020). The implementations of the scoring methods can be found in our software package and the analysis for reproducibility in the project repository in the folder “construction scoring methods” (cf. section “Code availability”).

### Benchmark experiments

#### Control gene selection bias on Tirosh-based gene signature scoring

We compared the strategies for the control gene selection of the methods ANS, Seurat, Seurat_AG, and Seurat_LVG across the four preprocessed single-cell-type PBMC subdata sets: B memory kappa, CD8+ T memory cell 2 (TM2), CD14 Mono, and NK 3 (“Data preprocessing” in Supplemental Methods). For each data set, we scored each gene in the top 8% of the highest expressed genes and thus converted the expression matrix to a score matrix. We visualized the mean scores and standard variation for each gene in increasing average expression order with the lineplot function of the plotting package seaborn (Fig. 1C; Supplemental Fig. S3; Waskom 2021). In addition, we used five subsets of PBMC data sets composed of (1) B cells, NK cells, and monocytes; (2) B intermediate, B memory, and B naive; (3) B intermediate kappa, B memory kappa, B naive kappa, B intermediate lambda, B memory lambda, and B naive lambda; (4) CD4^+^ CTL, CD4^+^ naive, CD4^+^ Treg, CD4^+^ proliferating, CD4^+^ TCM, and CD4^+^ TEM; and (5) CD8^+^ naive, CD8^+^ proliferating, CD8^+^ TCM, and CD8^+^ TEM cells. These data sets were used to evaluate the effect of filtering out highly expressed genes from the signatures before scoring. For construction of the signatures, we used signature genes associated with the original data set (https://atlas.fredhutch.org/nygc/multimodal-pbmc/). For the filtering strategies, we used (1) no filtering, (2) removal of marker genes from top 50 or (3) top 100 expressed genes, and (4) removal of marker genes from the top expression bin when genes are divided into 25 or (5) 50 bins. The ranking of genes was established by calculating mean expression across all cells within respective data sets and selecting the genes based on the set thresholds. Filtered signatures were then used for scoring using all Tirosh-based methods.

#### The influence of the cell-type proportions and batch effects on gene signature scoring

The CRC data set contains samples sequenced with two different sequencing chemistries. We used the different sequencing chemistries as surrogates for batch effects. Using a 100-gene signature for malignant cells with the smallest adjusted *P*-values and the highest log_2_FCs, we first scored each sample individually, and then, all samples were scored together. For each scoring method, we averaged the cell scores for each sample, cell type (malignant or nonmalignant), scoring mode (scoring samples individually or all together), and sequencing chemistry (SC3Pv2 or SC3Pv3). To evaluate the impact of batch effects, we used a two-sided Mann–Whitney *U* test to compare the sample-specific score averages of malignant cells between the two sequencing chemistries for each scoring method and scoring mode. We used the Python package statannotations (https://github.com/trevismd/statannotations) (version 0.4.4.) to run the Mann–Whitney *U* test and add statistical annotations in Figure 1D. We performed identical experiments on the preprocessed ESCC data set, excluding the batch effect component because of using uniform sequencing chemistries for all samples in this data set (Supplemental Fig. S4).

#### The influence of signature length on gene signature scoring

The next experiment aimed to assess the robustness of scoring methods to small signatures. Specifically, we sought to determine the minimum number of genes necessary to achieve perfect discrimination between malignant and nonmalignant genes using scores, thereby enabling binary classification. For each scoring method, we began by selecting a base signature for the malignant phenotype. Subsequently, we removed genes for which a valid control set could not be constructed, specifically those belonging to the *c/2* smallest or highest expressed genes, with *c* representing the size of the control set. The remaining genes were then sorted based on ascending adjusted *P*-value and descending log_2_FC. We iterated over the signature to ascertain the number of genes required, progressively expanding the range of elements considered from the initial subset containing only the first element until the final iteration encompassing the entire list. We computed AUCROC for each signature length, using both the scores and malignancy annotations. We ceased computing as soon as an AUCROC value of one was achieved. We conducted the analysis for the preprocessed CRC and ESCC data sets (Supplemental Figs. S3, S6).

#### Robustness to noise in gene expression signature

To compare the robustness of the gene signature scoring methods to noise, we utilized a 100-gene base signature distinguishing malignant and nonmalignant cells, which achieved an AUCROC of one for all scoring methods and iteratively replaced genes by noise. We selected noise genes based on their adjusted *P*-value > 0.01 and their |log_2_FC| ≤ 0.5 during DGEX, indicating that these genes lacked statistical significance in distinguishing malignant and nonmalignant cells. During each simulation run, we iteratively and randomly substituted the signature genes with randomly chosen genes, starting from the pure signature and progressing until the entire signature consisted of random genes. We conducted 20 simulation runs for each gene signature scoring method to ensure comprehensive results. The experiment was performed on preprocessed CRC and ESCC data sets (Supplemental Figs. S3, S6). Mean AUCROC values for each method and noise level across 20 simulation runs are presented in Supplemental Tables S3 and S4.

#### Comparability of signature score ranges for cell-type and cell-state annotation

To assess the information quantity and ranges of scores when scoring for multiple signatures, we considered unsupervised, score-based cell-type and cell-state annotation of four cancer data sets (BRCA, sCC, HGSOC, LUAD), a neuronal differentiation data set, and five PBMC data set subsets. We used cancer cell–state signatures published with the data sets. For the PBMC data sets, we constructed cell-type/-state-specific signatures using differential gene expression data accompanying the scRNA-seq data set (https://atlas.fredhutch.org/nygc/multimodal-pbmc/). The PBMC data set contains cell annotations at three levels of granularity: level one for broad cell types, level two for intermediate subtypes, and level three for the fine-grained subtypes. For each selected cell type in our PBMC subset data sets, we built signatures by identifying all corresponding level-3 subtypes and selecting differentially expressed genes (DEGs) with log_2_FC > 0.05 and adjusted *P*-value < 0.01. We created the final signature by combining DEGs from these level-3 subtypes through set union operations. When comparing overlapping versus nonoverlapping signatures, we additionally created a version in which genes appearing in multiple cell-type signatures were removed, as these shared genes could bias scoring across cells.

For each data set, we calculated scores for every cell using all signatures and scoring methods, each signature being associated with a distinct cell type/state (for score distributions, see Supplemental Figs. S10, S13). We assigned each cell to the state associated with that cell's highest-scoring signature (argmax), enabling unsupervised annotation.

To evaluate the information content within the scores, we employed scikit-learn's (v1.2.0) cross-validated supervised logistic regression (LogisticRegressionCV with C = none and stratified k-fold cross-validation with k = 10, random_state = 42) to assess how well the scores could predict the annotated cell states/types. For each scoring method, the mean cross-validation performance represented its supervised annotation performance, namely, its information content. We used balanced accuracy and weighted F1-score metrics to evaluate both supervised and unsupervised approaches. We defined scale imbalance as the difference between the cross-validated supervised classification performance and unsupervised score-based annotation performance. Results for nonoverlapping and overlapping signatures are presented in Supplemental Tables S6 and S7, respectively.

### Case study: selection of cancer EMT cells and cancer-specific EMT signature establishment

For the entire case study, we used ANS for evaluation with control sets of size *c* = 100. The generation of the cancer-specific EMT signature consisted of three main steps. In the first step, we classified the malignant cells as EMT-expressing or as nonexpressing. Based on this classification, we explored the gene expression differences between EMT-expressing cancer cells and non-EMT cancer cells, as well as between EMT cancer cells and CAFs in the ESCC and LUAD_Xing data sets, which led to the establishment of a cancer EMT-specific signature. In the last step, we validated the established cancer EMT-specific signature on independent validation data sets including BRCA and CRC. Further details on the analysis can be found in the Supplemental Methods.

#### Evaluation of clinical relevance of cancer-specific EMT signature

To analyze the link between de novo EMT signature and clinical characteristics of samples in the TCGA data sets, we downloaded pancancer normalized RNA-seq by expectation maximization (RSEM) gene expression data from the GDC portal (https://portal.gdc.cancer.gov/). Tumor purity estimates were downloaded from the supplemental data of the TCGA PanCanAtlas (https://gdc.cancer.gov/about-data/publications/pancanatlas). Primary and metastatic samples were identified based on the TCGA sample barcode information from https://gdc.cancer.gov/resources-tcga-users/tcga-code-tables/sample-type-codes. Clinical information on the histological subtypes was retrieved from Liu et al. (2018). The EMT signatures were scored in each corresponding cancer data set as the sum of the standardized log_2_(x + 1)-transformed gene expression of signature genes. Association between the scores and tumor purity was calculated using Spearman's rank correlation in R Statistical Software (v4.1.2) (R Core Team 2021). The association between scores across primary and metastatic tumors was evaluated using Wilcoxon's test implemented in R Statistical Software (v4.1.2) (R Core Team 2021), excluding paired samples for fair comparison. The association between EMT scores and histological subtypes was evaluated using the two-sided Kruskal–Wallis test with FDR correction in cancer types for which this information was available. The analysis was performed using R Statistical Software (v4.1.2) (R Core Team 2021).

To verify the association of the calculated scores and patient survival, we used multivariate Cox regression analysis with the calculated EMT scores, disease stage, and patient age, stratified by cancer type, in cancer types for which disease stage and patient age information was available. The analysis was repeated using the multivariate model with addition of hallmark EMT-based scores calculated with as described above. Survival analysis was performed using the survival R package (https://cran.r-project.org/package=survival).

Treatment response information was obtained using the TCGAbiolinks R package (https://rdrr.io/bioc/TCGAbiolinks). The response information for chemotherapy and immunotherapy was encoded as follows: one, complete response; two, partial response; three, stable disease; and, four, clinical progressive disease. The values were then associated with the EMT-scores using Spearman's rank correlation (using R) for each cancer type individually.

### Score-based cell-type labeling in neuronal differentiation data set

The preprocessed subsampled data set was downloaded as h5ad object from Zenodo (https://zenodo.org/records/4333872). To shorten the computation time, the following analysis was done on 40% of the downloaded data set. Cell-type-specific marker genes were identified with SCANPY's function rank_genes_groups, using a Wilcoxon's test to select 100 marker genes (adjusted *P*-value < 0.05, log_2_FC > 0) for each cell type. The signature lists were used for scoring with all of the benchmarked methods, followed by cell-type labeling based on the maximal score for each cell. Balanced accuracy was calculated using assigned labels and the ground-truth cell-type annotations. ANS scores were then used to generate PHATE embeddings with default parameters. Similarly, we used log_2_(x + 1) gene expression values to generate PHATE embeddings based on gene expression. Pseudotime was calculated using cdist function from SciPy (scipy.spatial.distance) package, with one of the cells of type “FPP” as the root and the Euclidean distance, for both score-based and gene expression-based embeddings. To evaluate similarity of respective cell types, we aggregated gene expression per cell type and computed pairwise distances between the profiles using pdist function from SciPy (scipy.spatial.distance) package, with (1 − *R*) as metric, where *R* is Pearson correlation coefficient. Then, average linkage was used to generate the dendrogram of cell-type similarities.

### Statistical information

The statistical tools, methods, and threshold for each analysis are explicitly described with the results or detailed in the figure legends or the Methods.

### Code availability

The Python package published in the GitHub repository (https://github.com/BoevaLab/ANS_signature_scoring) includes the implementations of all considered scoring methods. The repository also contains the R implementation of ANS. The experiments have been conducted in Python, and the code for reproducibility of the experiments and visualization can be found in the GitHub repository (https://github.com/BoevaLab/ANS_supplementary_information). In addition, the ANS packages and the code used for experiments and visualization are available at Zenodo (https://doi.org/10.5281/zenodo.17488720 and https://doi.org/10.5281/zenodo.17488855) and as Supplemental Code. Note that all package versions and environment information are included in the repository (construction_scoring_methods/session_info.txt for R, and environment.yml for Python). The method's tutorial is available at https://boevalab.github.io/ANS_signature_scoring/.

## Supplemental Material

Supplement 1

Supplement 2

Supplement 3

Supplement 4
