# Supplementary figures and images for: Robust and efficient annotation of cell states through gene signature scoring
Source: Genome Res. 2026 Mar;36(3):630–44. doi: 10.1101/gr.280926.125 (PMC12951948; doi:10.1101/gr.280926.125)

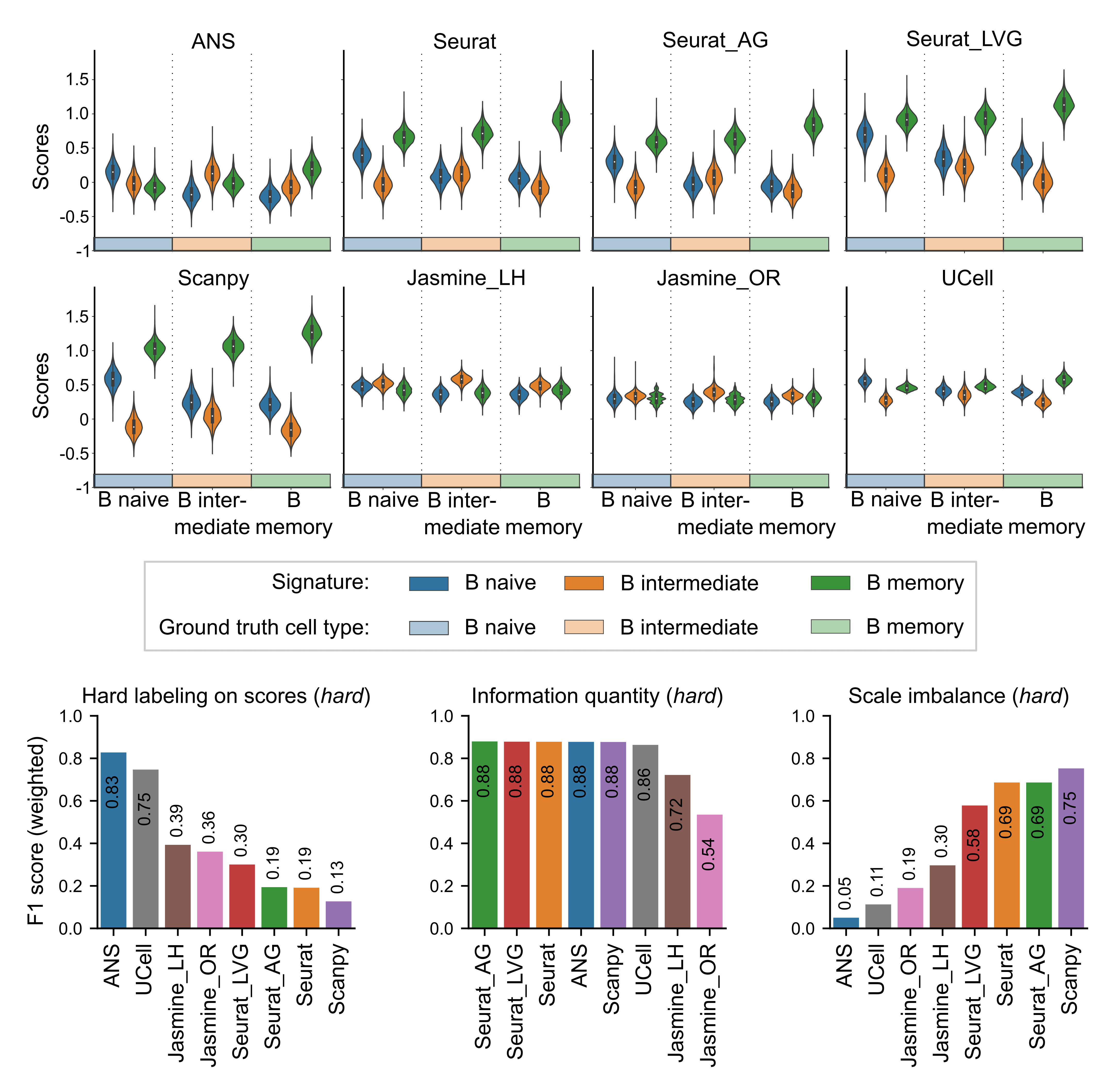

Supplement: Supplement 1 [file Supplemental_Code.zip › ANS_code_main_and_supplementary/ANS_signature_scoring_package/docs/img/website_figure.png]
