# Supplementary material for: Robust and efficient annotation of cell states through gene signature scoring
Source: Genome Res. 2026 Mar;36(3):630–44. doi: 10.1101/gr.280926.125 (PMC12951948; doi:10.1101/gr.280926.125)
Supplement: Supplement 2 [file Supplemental_Materials.pdf]

Supplemental Material

ANS: Adjusted Neighborhood Scoring to improve gene signature-based cell annotation in single-cell RNA-seq data

Laure Ciernik[1,2,3]*, Agnieszka Kraft[1,4,5,†*], Florian Barkmann[1], Josephine Yates[1,4,7‡], and Valentina Boeva[1,4,6,7]#

[1]ETH Zurich, Department of Computer Science, Institute for Machine Learning, 8092 Zurich, Switzerland;
[2]Machine Learning Group, Technische Universität Berlin, 10587 Berlin, Germany;
[3]Hector Fellow Academy, 76131 Karlsruhe, Germany;
[4]Swiss Institute of Bioinformatics (SIB), 8092 Zurich, Switzerland;
[5]University Hospital Zurich, Department of Thoracic Surgery, 8092 Zurich;
[6]INSERM, U1016, Cochin Institute, CNRS UMR8104, Paris Descartes University, 75014 Paris, France;
[7]ETH AI Center, ETH Zürich, 8092 Zurich, Switzerland;
†Current affiliation: Medical University of Vienna, Institute of Artificial Intelligence, Center for Medical Data Science (CEDAS), 1090 Vienna, Austria and CeMM Research Center for Molecular Medicine of the Austrian Academy of Sciences, 1090 Vienna, Austria
‡Current affiliation: Eric and Wendy Schmidt Center, The Broad Institute of MIT and Harvard, 02141 Cambridge, Massachusetts, USA

*Equal contribution
#Correspondence should be addressed to Valentina Boeva, valentina.boeva@inf.ethz.ch

Contents

Supplemental Methods **2**
Design of the benchmark 2
Datasets preprocessing 2
Information quantity and scale imbalance 3
Classification of cancer EMT cells 3
Gene signature refinement with scores of single signature genes 4
Establishment of a cancer-specific EMT signature in ESCC and LUAD_Xing 4
Supplemental Tables **6**
Supplemental Figures **22**
References **57**

Supplemental Methods

Design of the benchmark

We benchmarked the methods in six scenarios (Figure 1A, Table 1). In the first scenario, we showed the implication of control gene selection on gene signature scoring. In the second and third, we investigated the influence of the data composition and batch effects on scoring. In the fourth and fifth scenarios, we analyzed the impact of the variability in the gene signature length on the resulting scores. Finally, in the sixth scenario, we examined the scores produced by different methods when scoring multiple gene signatures and evaluated the performance of cell score-based classifiers to annotate cell types and cell states. For the first scenario, we used a preprocessed scRNA-seq dataset of peripheral blood mononuclear cells (Hao et al. 2021) (PBMC), where cell-type signatures were derived from published differentially expressed genes per cell type. The next four scenarios were carried out on preprocessed scRNA-seq datasets of colorectal carcinoma (CRC) (Pelka et al. 2021) and esophageal squamous cell carcinoma (ESCC) (Zhang et al. 2021), using signatures based on differentially expressed genes between malignant and non-malignant cells. In the sixth scenario we used scRNA-seq datasets of PBMC (Hao et al. 2021) and four cancer types: breast carcinoma (BRCA) (Wu et al. 2021), high-grade serous ovarian cancer (HGSOC) (Vázquez-García et al. 2022), cutaneous squamous cell carcinoma (cSCC) (Ji et al. 2020), and lung adenocarcinoma (LUAD) (Kim et al. 2020), where cell state signatures were based on cancer programs reported in the original studies. In addition, we included a benchmark of score-based cell type annotation using publicly available preprocessed scRNA-seq dataset of neuronal differentiation (Jerber et al. 2021).

Datasets preprocessing

Cancer datasets and the PBMC dataset were preprocessed differently. For the cancer datasets, except HGSOC, we used the preprocessing module of CanSig (Barkmann et al. 2025) with the default parameters as the first preprocessing step (https://github.com/BoevaLab/CanSig-benchmark and https://boevalab.github.io/CanSig-benchmark/). In brief, we applied standard quality control metrics and removed potential misannotated cells using CNV inference; the preprocessing steps are extensively described in the original paper. Additionally, we filtered genes based on a minimum expression in 1% of cells, and normalized cell reads using a shift logarithm $\log_2(x+1)$ with the mean total raw count per cell as the target sum, as described and implemented by Ahlmann-Eltze and Huber (Ahlmann-Eltze and Huber 2023). For HGSOC, due to its size, we directly applied the 1% gene filtering and log-normalization on the cancer cells from the GEO data (GSE180661), skipping the CanSig preprocessing step. We demonstrated that these preprocessing choices are robust, as replacing the CanSig preprocessing pipeline with alternative approaches (e.g., Curated Cancer Cell Atlas preprocessing, https://www.weizmann.ac.il/sites/3CA/) or omitting it did not significantly affect the relative performance of the scoring methods (Supplemental Fig. S26-S28). Regarding the PBMC dataset, we created several sub-datasets based on cell type and state annotations. The first sub-dataset consisted of B cells, monocytes, and natural killer cells, while the second subdataset contained B cell states (B naive, B intermediate, and B memory cells). The third and fourth sub-datasets consisted of CD4+ T cell states (cytotoxic T lymphocytes (CTL), naive, proliferating, central memory T cells (TCM), effector memory T

cells (TEM), and regulatory T cells (Treg)) and CD8+ T cell states (naive, proliferating, TCM, TEM). All sub-datasets were preprocessed as follows: Initially, we filtered low-quality cells according to the guidelines provided in chapter 6.3 of the book "Single-cell Best Practices" by Heumos et al. (Heumos et al. 2023). A threshold of 5 median absolute deviations (MADs) was selected for filtering covariates, including total counts, number of genes with positive counts in a cell, and the percentage of mitochondrial counts. Similar to the cancer datasets, we filtered genes based on a minimum expression in 1% of cells and normalized the cell reads using the shift logarithm with the mean total raw count per cell as the target sum. For each analysed dataset, all samples were preprocessed together.

Information quantity and scale imbalance

We refer to the cross-validated accuracy of the best linear classifier based on the gene signature scores as information quantity of the gene signatures, *i.e.*, the feasibility of accurate cell annotation based on the scores. For instance, information quantity close to 1 corresponded to the perfect supervised annotation of cell states or types based on gene signature scores. Further, we define the difference between information quantity and the unsupervised score-based annotation as the scale imbalance. Such imbalance occurs when the supervised performance based on the used scores does not fully translate into unsupervised labeling accuracy. For instance, for methods with information content close to 1, the scores contain the information necessary to accurately distinguish cell types, but their ranges vary substantially across cell types, *e.g.*, one cell type may consistently present higher score values compared to others. As a result, assignment based on the maximal score leads to overclassification with the particular cell type. Conversely, scoring methods whose score-based label assignment performance was close to the calculated information quantity, were considered to produce gene signature scores within comparable ranges, indicating a minor score scale imbalance.

Classification of cancer EMT cells

As a first step in the signature establishment, we selected cancer cells expressing EMT, called cancer EMT cells. We computed scores for ESCC with the dataset-specific mesenchymal signature (Zhang et al. 2021). We scored the entire dataset and the subset of malignant cells using ANS. Cancer cells within the 10% highest scoring cells on the entire dataset and in the subset of malignant cells were classified as cancer EMT cells. The 3,860 cancer EMT cells out of 41,399 cancer cells (9.32%) and 178,109 total cells (2.17%), stem from 53 out of 54 samples. For LUAD_Xing and CRC, no dataset-specific mesenchymal signatures were reported. Therefore, we selected the cancer cells expressing EMT as follows. We scored the datasets for seven pan-cancer EMT signatures: Hallmark EMT signature (Liberzon et al. 2015), pEMT gene module (Barkley et al. 2022), and five other EMT signatures (Gröger et al. 2012; Tan et al. 2014; Mak et al. 2016; Foroutan et al. 2017; Hollern et al. 2018). The pan-cancer EMT signatures were found on EMTome (Vasaikar et al. 2021). For each pan-cancer signature, we ranked the cancer cells by ANS scores and computed for each cell the median rank. Finally, 10% of cancer cells with the smallest median signature ranks were classified as cancer EMT cells. We selected the median to avoid outlier ranks of the pan-cancer signature. For BRCA, we used the state labels provided by the original paper (Wu et al. 2021) and excluded cells falling in the “grey zone” of the EMT program expression ("GM3" in the original paper). We considered only samples assigned to the “Basal” subtype and defined the ”grey

zone" to be cells not labeled with "GM3", but having a signature score for GM3 above 0.15 (scoring was applied on all samples similar in the original paper). In the LUAD_Xing dataset, 822 out of 8,205 cancer (10.02%) and 55,659 total (1.48%) cells were classified as EMT. All 19 samples contained cancer EMT cells. In the CRC dataset, 4,328 out of 43,252 cancer (10.01%) and 140,090 total (3.09%) cells were classified as cancer EMT cells distributed over all 60 samples. In the BRCA dataset, 2031 out of 5115 cancer (39,71%) and 27458 total (7,40%) cells were classified as cancer EMT cells distributed over all 7 samples. Datasets composition is shown in Supplemental Fig. S23.

Gene signature refinement with scores of single signature genes

Gene signature refinement aims to remove genes from a signature to achieve a higher discriminating power of the signature scores. We mainly used gene signature refinement in the case study, however, it could be applied to any signature. During the refinement of a signature associated with a subset of cells, gene signature scoring is applied to each signature gene individually. A signature gene remained in the signature only if the scores for the desired subset of cells were significantly higher than those for another subset of interest. To test for distributional shifts, we used the one-sided Mann-Whitney U (SciPy, version 1.9.3) with Benjamini-Hochberg FDR correction for p-values (statsmodels, version 0.14.1)) with alpha 1×10^{-5}. The process requires availability of cell annotations for the desired subsets of cells.

Establishment of a cancer-specific EMT signature in ESCC and LUAD_Xing

The signature establishment consisted of finding differentially expressed genes between cancer cells expressing EMT and CAFs as well as cancer cells not expressing EMT in ESCC and LUAD_Xing. The cancer EMT cell classification for all datasets is provided above. We first started by finding a cancer-specific signature for ESCC, then found one for LUAD_Xing. Finally, we merged the ESCC and LUAD_Xing-specific cancer EMT signature and refined it again on ESCC (see previous section). For differential gene expression (DGEX) analysis between two cell groups of interest, we used two-sided Wilcoxon rank-sum (Mann-Whitney U) tests (Soneson and Robinson 2018) with tie correction and Benjamini-Hochberg correction implemented in SCANPY's rank_genes_groups.

Cancer-specific EMT signature for ESCC: First, we created two subsets of the ESCC dataset: cancer EMT cells and CAFs and all cancer cells. Let A be the set of DGEX genes ($\log_2$FC >= 2 and an adjusted p-value < 0.001) between cancer EMT cells and CAFs. Let B be the set of DGEX genes ($\log_2$FC >= 1.5 and an adjusted p-value < 0.001) between cancer EMT cells and cancer cells not expressing EMT. We intersected sets A and B, called gene set C, and removed all genes from C that are DGEX ($\log_2$FC >= 2 and an adjusted p-value < 0.001) in T and Myeloid cells versus all other cells (*i.e.*, removing T- and Myeloid cell-specific genes). The reduced gene set C, called gene set D, scored high for many cancer cells and thus required more refinement to distinguish the cancer EMT cells from the other cancer cells. Let the set X be all cancer cells that scored above *0.2* for the gene set D. We selected all DGEX genes ($\log_2$FC >= 1.5 and an adjusted p-value < 0.001), called gene set E, in X between cancer EMT cells and cancer cells not expressing EMT. We scored each gene in $E - D$ (n=451) independently and kept genes with significantly higher scores for cancer EMT cells than for CAFs, cancer cells not expressing EMT, and the rest of the cells (process of signature refinement, described above). We merged the gene sets D and E, resulting in our ESCC-

specific cancer EMT signature. The detailed procedure can be found in the Jupyter Notebook find_cancer_emt_signature_ESCC.ipynb in the GitHub repository https://github.com/BoevaLab/ANS_supplementary_information.

Cancer-specific EMT signature for LUAD_Xing: For the LUAD_Xing-specific cancer EMT signature, we followed a procedure similar to that for ESCC. First we created two subsets of the LUAD_Xing dataset: cancer EMT cells and CAFs and all cancer cells. Let A be the set of DGEX genes ($\log_2$FC >= 2 and an adjusted p-value < 0.001) between cancer EMT cells and CAFs. Let B be the set of DGEX genes ($\log_2$FC >= 1 and an adjusted p-value < 0.001) between cancer EMT cells and cancer cells not expressing EMT. We unioned gene sets A and B, called gene set C. For each cell type X not equal to cancer EMT cells, we selected DGEX genes ($\log_2$FC >= 1 and an adjusted p-value < 0.001) of X versus cancer EMT cells and removed them from gene set C. The resulting gene set corresponded to our LUAD_Xing-specific cancer EMT signature. The detailed procedure can be found in the Jupyter Notebook find_cancer_emt_signature_LUAD.ipynb in the GitHub repository https://github.com/BoevaLab/ANS_supplementary_information.

Joining cancer-specific EMT signatures for ESCC and LUAD_Xing and refinement on ESCC: To obtain a signature that can distinguish between cancer EMT cells and CAFs, and cancer EMT cells and cancer cells that do not express EMT, we unioned the ESCC- and LUAD_Xing-specific cancer EMT signatures. We scored for each gene in the unioned signature in ESCC (n=267) and only kept a gene if it had significantly higher scores for cancer EMT cells than for CAFs, cancer cells not expressing EMT, and the rest of the cells (signature refinement). We excluded the insignificant genes and incorporated the LUAD_Xing-specific cancer EMT signature genes absent in the preprocessed ESCC dataset and the ESCC-specific cancer EMT signature genes absent in the preprocessed LUAD_Xing dataset. Supplemental Table S5 shows the 135 ESCC- and LUAD_Xing-specific cancer EMT signature genes. The detailed procedure of signature union and refinement can be found in the Jupyter Notebook union_ESCC_and_LUAD_specific_EMT_signature_and_refine_on_ESCC.ipynb in the GitHub repository https://github.com/BoevaLab/ANS_supplementary_information.

Supplemental Tables

Supplemental Table S1: Pearson correlation static and p-values of Figure 1b.

Scoring method	Dataset	r	p	df	ci_lower	ci_upper
**ANS**	CRC	1	0	140088	1	1
**ANS**	ESCC	1	0	178107	1	1
**Seurat**	CRC	0.99934427	0	140088	0.99933737	0.9993511
**Seurat**	ESCC	0.99962126	0	178107	0.99961772	0.99962476
**Jasmine_LH**	CRC	1	0	140088	1	1
**Jasmine_LH**	ESCC	1	0	178107	1	1
**Jasmine_OR**	CRC	0.99999987	0	140088	0.99999987	0.99999987
**Jasmine_OR**	ESCC	0.99999943	0	178107	0.99999942	0.99999943
**UCell**	CRC	1	0	140088	1	1
**UCell**	ESCC	1	0	178107	1	1

Supplemental Table S2: Mann-Whitney *U* static and p-values of Figure 1d.

Scoring methods	Scoring mode	MannWhitney*U* p-val	MannWhitney *U* statistic	Short
**ANS**	Scoring all samples together	0.271755472	317	ns
**ANS**	Scoring each sample individually (preprocessed together)	0.003186054	574	**
**Seurat**	Scoring all samples together	0.278712777	318	ns
**Seurat**	Scoring each sample individually (preprocessed together)	0.001695527	586	**
**Seurat_AG**	Scoring all samples together	0.322988454	324	ns
**Seurat_AG**	Scoring each sample individually (preprocessed together)	0.001521351	588	**
**Seurat_LVG**	Scoring all samples together	0.315306953	323	ns
**Seurat_LVG**	Scoring each sample individually (preprocessed together)	3.09E-05	651	****
**SCANPY**	Scoring all samples together	0.44311231	338	ns
**SCANPY**	Scoring each sample individually (preprocessed together)	0.023255079	531	*
**Jasmine_LH**	Scoring all samples together	0.019675908	239	*
**Jasmine_LH**	Scoring each sample individually (preprocessed together)	0.067783459	503	ns
**Jasmine_OR**	Scoring all samples together	0.521885749	346	ns
**Jasmine_OR**	Scoring each sample individually (preprocessed together)	0.981075915	389	ns
**UCell**	Scoring all samples together	0.278712777	318	ns
**UCell**	Scoring each sample individually (preprocessed together)	0.278712777	318	ns

Supplemental Table S3. AUCROC for noisy signatures in CRC data. Mean across 20 experiments is reported for each scoring method. Results corresponding to signatures with 85% of noisy genes are reported in bold.

Purity	ANS mean	Jasmine_LH mean	Jasmine mean	SCANPY mean	Seurat_AG mean	Seurat_LVG mean	Seurat mean	UCell mean
100	1	0.998	0.964	1	1	0.999	1	1
95	1	0.982	0.948	1	1	0.999	1	1
90	1	0.991	0.961	1	1	0.998	1	0.999
85	1	0.995	0.967	1	1	0.998	1	0.999
80	1	0.989	0.96	1	1	0.997	1	0.999
75	1	0.988	0.978	1	1	0.996	1	0.999
70	0.999	0.985	0.98	1	0.999	0.993	0.999	0.998
65	0.999	0.992	0.98	0.999	0.999	0.988	0.999	0.997
60	0.999	0.991	0.985	0.999	0.999	0.988	0.999	0.996
55	0.998	0.989	0.983	0.998	0.999	0.978	0.998	0.995
50	0.998	0.988	0.987	0.997	0.998	0.969	0.997	0.992
45	0.996	0.983	0.986	0.996	0.997	0.947	0.997	0.992
40	0.995	0.981	0.982	0.994	0.993	0.924	0.993	0.988
35	0.991	0.98	0.979	0.991	0.99	0.853	0.99	0.979
30	0.983	0.965	0.975	0.975	0.981	0.763	0.983	0.973
25	0.969	0.947	0.954	0.968	0.966	0.698	0.962	0.944
20	0.952	0.917	0.932	0.936	0.927	0.553	0.937	0.911
**15**	**0.883**	**0.828**	**0.832**	**0.848**	**0.872**	**0.392**	**0.869**	**0.851**

10	0.77	0.745	0.812	0.756	0.744	0.29	0.781	0.746
5	0.675	0.607	0.651	0.601	0.599	0.174	0.622	0.607
0	0.423	0.495	0.502	0.435	0.428	0.135	0.443	0.463

Supplemental Table S4. AUCROC for noisy signatures in ESCC data. Mean across 20 experiments is reported for each scoring method. Results corresponding to signatures with 85% of noisy genes are reported in bold.

Purity	ANS mean	Jasmine_LH mean	Jasmine mean	SCANPY mean	Seurat_AG mean	Seurat_LVG mean	Seurat mean	UCell mean
100	1	0.992	0.981	1	1	0.998	1	1
95	1	0.983	0.973	1	1	0.998	1	1
90	1	0.988	0.979	1	1	0.998	1	1
85	1	0.985	0.98	1	1	0.997	1	1
80	1	0.99	0.982	1	1	0.997	0.999	1
75	1	0.988	0.989	1	0.999	0.996	0.999	1
70	1	0.992	0.99	0.999	0.999	0.995	0.999	0.999
65	0.999	0.991	0.989	0.999	0.999	0.994	0.999	0.999
60	0.999	0.988	0.99	0.999	0.999	0.991	0.999	0.999
55	0.999	0.991	0.988	0.999	0.999	0.988	0.999	0.998
50	0.999	0.992	0.987	0.998	0.999	0.985	0.998	0.997
45	0.997	0.989	0.988	0.997	0.998	0.98	0.996	0.996
40	0.997	0.988	0.987	0.996	0.996	0.97	0.995	0.993
35	0.995	0.972	0.983	0.994	0.995	0.94	0.992	0.986
30	0.984	0.97	0.973	0.989	0.99	0.914	0.988	0.981
25	0.976	0.951	0.968	0.981	0.976	0.868	0.981	0.954
20	0.944	0.929	0.92	0.957	0.945	0.793	0.942	0.937
**15**	**0.88**	**0.801**	**0.849**	**0.899**	**0.906**	**0.682**	**0.862**	**0.873**

10	0.797	0.735	0.736	0.788	0.788	0.476	0.767	0.766
5	0.611	0.543	0.58	0.624	0.614	0.329	0.625	0.589
0	0.355	0.394	0.414	0.374	0.368	0.191	0.35	0.375

Supplemental Table S5: Gene signatures for each dataset used in the comparable score range experiment. **See separate file linked to the article: SupplTableS5.xlsx**

Supplemental Table S6. Cell type annotation performance comparisons (balanced accuracy and f1-score weighted) based on **non-overlapping** signatures' scores and in the information of signatures' scores computed with logistic regression (std over folds). Highest value per row shown in bold.

		Balanced Accuracy							
		**ANS**	**Jasmine_LH**	**Jasmine_OR**	**SCANPY**	**Seurat**	**Seurat_AG**	**Seurat_LVG**	**UCell**
**Hard labeling score**	**BRCA (6 states)**	**0.560**	0.496	0.455	0.486	0.516	0.524	0.534	0.402
	**HGSOC (8 states)**	**0.638**	0.515	0.394	0.564	0.630	0.632	0.620	0.535
	**LUAD (3 states)**	0.897	0.885	0.791	**0.914**	0.894	0.896	0.825	0.839
	**cSCC (4 states)**	**0.843**	0.779	0.750	0.742	0.827	0.829	0.794	0.699
	**B, Mono-cytes, NK**	0.997	0.967	0.921	**0.999**	0.977	0.986	0.997	0.999
	**B cell subtypes**	**0.836**	0.538	0.480	0.333	0.338	0.338	0.340	0.700
	**CD4+ T cell subtypes**	**0.645**	0.551	0.487	0.570	0.561	0.558	0.539	0.600
	**CD8+ T cell subtypes**	**0.823**	0.649	0.488	0.405	0.438	0.427	0.299	0.336
**Information quantity (logistic regression 10-fold CV)**	**BRCA (6 states)**	0.605 (0.063)	0.589 (0.053)	0.527 (0.054)	0.597 (0.062)	0.589 (0.048)	0.6 (0.053)	0.6 (0.053)	**0.625 (0.043)**
	**HGSOC (8 states)**	0.604 (0.066)	0.531 (0.048)	0.387 (0.038)	**0.606 (0.066)**	0.604 (0.067)	0.605 (0.067)	0.602 (0.071)	0.592 (0.059)
	**LUAD (3 states)**	0.892 (0.068)	0.87 (0.097)	0.811 (0.083)	0.878 (0.066)	0.882 (0.067)	0.89 (0.069)	**0.923 (0.038)**	0.899 (0.049)
	**cSCC (4 states)**	0.841 (0.066)	0.824 (0.075)	0.772 (0.074)	**0.844 (0.061)**	0.841 (0.065)	0.843 (0.065)	0.839 (0.066)	0.832 (0.064)
	**B, Mono-cytes, NK**	**0.999 (0.001)**	**0.999 (0.001)**	0.998 (0.001)	**0.999 (0.001)**	**0.999 (0.001)**	**0.999 (0.001)**	**0.999 (0.001)**	**0.999 (0.001)**
	**B cell subtypes**	0.853 (0.022)	0.738 (0.024)	0.54 (0.044)	0.847 (0.023)	**0.853 (0.026)**	0.851 (0.025)	0.851 (0.024)	0.826 (0.024)
	**CD4+ T cell subtypes**	0.681 (0.023)	0.579 (0.041)	0.539 (0.063)	0.685 (0.042)	0.685 (0.041)	**0.691 (0.026)**	0.69 (0.025)	0.663 (0.055)
	**CD8+ T cell subtypes**	**0.829 (0.043)**	0.695 (0.068)	0.603 (0.095)	0.801 (0.052)	0.806 (0.067)	0.817 (0.047)	0.826 (0.05)	0.819 (0.048)

		F1 Score							
		ANS	Jasmine_LH	Jasmine_OR	SCANPY	Seurat	Seurat_AG	Seurat_LVG	UCell
**Hard labeling score**	**BRCA (6 states)**	**0.492**	0.453	0.407	0.426	0.457	0.464	0.472	0.309
	**HGSOC (8 states)**	**0.599**	0.547	0.492	0.513	0.591	0.587	0.555	0.552
	**LUAD (3 states)**	0.861	0.853	0.869	0.880	0.861	0.862	0.796	**0.898**
	**cSCC (4 states)**	0.825	0.800	0.751	0.784	0.828	**0.832**	0.820	0.759
	**B, Mono-cytes, NK**	0.996	0.984	0.962	0.999	0.958	0.975	0.996	**0.999**
	**B cell subtypes**	**0.834**	0.474	0.345	0.104	0.115	0.117	0.125	0.757
	**CD4+ T cell subtypes**	**0.457**	0.216	0.255	0.354	0.362	0.361	0.352	0.448
	**CD8+ T cell subtypes**	**0.825**	0.504	0.248	0.398	0.484	0.460	0.308	0.306
**Information quantity (logistic regression 10-fold CV)**	**BRCA (6 states)**	0.598 (0.067)	0.588 (0.059)	0.528 (0.055)	0.586 (0.063)	0.581 (0.047)	0.592 (0.054)	0.591 (0.057)	**0.618 (0.045)**
	**HGSOC (8 states)**	0.717 (0.027)	0.661 (0.027)	0.575 (0.038)	**0.718 (0.026)**	0.715 (0.029)	0.717 (0.027)	0.714 (0.031)	0.709 (0.024)
	**LUAD (3 states)**	0.907 (0.012)	0.909 (0.018)	0.904 (0.015)	0.899 (0.016)	0.905 (0.012)	0.903 (0.014)	**0.918 (0.013)**	0.916 (0.011)
	**cSCC (4 states)**	0.869 (0.033)	0.857 (0.037)	0.824 (0.037)	0.869 (0.029)	0.869 (0.032)	**0.87 (0.033)**	0.867 (0.034)	0.861 (0.029)
	**B, Mono-cytes, NK**	**0.999 (0.001)**	**0.999 (0.001)**	**0.999 (0.001)**	0.999 (0.0)	**0.999 (0.001)**	0.999 (0.0)	**0.999 (0.001)**	**0.999 (0.001)**
	**B cell subtypes**	**0.894 (0.023)**	0.799 (0.025)	0.618 (0.038)	0.89 (0.023)	0.893 (0.026)	0.892 (0.024)	0.893 (0.023)	0.874 (0.025)
	**CD4+ T cell subtypes**	0.731 (0.015)	0.603 (0.018)	0.562 (0.048)	0.741 (0.016)	0.745 (0.013)	0.746 (0.014)	**0.747 (0.014)**	0.716 (0.012)
	**CD8+ T cell subtypes**	**0.913 (0.015)**	0.819 (0.014)	0.734 (0.036)	0.906 (0.016)	0.909 (0.016)	0.909 (0.016)	0.907 (0.017)	0.896 (0.008)

Supplemental Table S7. Cell type annotation performance comparisons (balanced accuracy and f1-score weighted) based on **overlapping** signatures' scores and in the information of signatures' scores computed with logistic regression (std over folds). Highest value per row shown in bold.

		Balanced Accuracy							
		**ANS**	**Jasmine_LH**	**Jasmine_OR**	**SCANPY**	**Seurat**	**Seurat_AG**	**Seurat_LVG**	**UCell**
**Hard labeling score**	**BRCA (6 states)**	**0.575**	0.522	0.515	0.519	0.533	0.536	0.543	0.411
	**HGSOC (8 states)**	**0.646**	0.570	0.418	0.577	0.637	0.637	0.626	0.553
	**LUAD (3 states)**	0.902	0.874	0.841	**0.912**	0.897	0.902	0.836	0.850
	**cSCC (4 states)**	**0.846**	0.785	0.757	0.742	0.821	0.827	0.792	0.696
	**B, Mono-cytes, NK**	0.998	0.997	0.992	**0.999**	0.988	0.993	0.998	0.998
	**B cell subtypes**	**0.850**	0.667	0.442	0.333	0.412	0.378	0.413	0.679
	**CD4+ T cell subtypes**	**0.669**	0.585	0.581	0.167	0.189	0.198	0.170	0.208
	**CD8+ T cell subtypes**	**0.784**	0.732	0.722	0.447	0.390	0.376	0.438	0.415
**Information quantity (logistic regression 10-fold CV)**	**BRCA (6 states)**	0.612 (0.068)	0.608 (0.044)	0.576 (0.049)	0.602 (0.065)	0.605 (0.06)	0.611 (0.06)	0.606 (0.066)	**0.636 (0.053)**
	**HGSOC (8 states)**	0.612 (0.069)	0.537 (0.045)	0.395 (0.039)	**0.613 (0.068)**	0.611 (0.071)	0.612 (0.071)	0.607 (0.073)	0.6 (0.059)
	**LUAD (3 states)**	0.893 (0.07)	0.856 (0.087)	0.86 (0.062)	0.881 (0.066)	0.882 (0.066)	0.882 (0.066)	**0.914 (0.042)**	0.913 (0.042)
	**cSCC (4 states)**	0.84 (0.066)	0.825 (0.077)	0.779 (0.072)	**0.842 (0.064)**	0.838 (0.064)	**0.842 (0.064)**	0.839 (0.066)	0.834 (0.065)
	**B, Mono-cytes, NK**	**0.999 (0.001)**	**0.999 (0.001)**	0.998 (0.001)	**0.999 (0.001)**	**0.999 (0.001)**	**0.999 (0.001)**	**0.999 (0.001)**	**0.999 (0.001)**
	**B cell subtypes**	0.884 (0.025)	0.726 (0.021)	0.457 (0.045)	0.865 (0.021)	**0.885 (0.025)**	0.883 (0.027)	0.881 (0.027)	0.857 (0.018)
	**CD4+ T cell subtypes**	0.76 (0.027)	0.624 (0.042)	0.57 (0.053)	0.728 (0.041)	0.757 (0.023)	**0.762 (0.026)**	0.75 (0.025)	0.734 (0.036)
	**CD8+ T cell subtypes**	0.864 (0.04)	0.762 (0.065)	0.719 (0.085)	0.847 (0.056)	0.866 (0.042)	0.869 (0.043)	**0.875 (0.039)**	0.854 (0.041)

		F1 Score							
		**ANS**	**Jasmine_LH**	**Jasmine_OR**	**SCANPY**	**Seurat**	**Seurat_AG**	**Seurat_LVG**	**UCell**
**Hard labeling score**	**BRCA (6 states)**	**0.503**	0.470	0.458	0.447	0.473	0.475	0.479	0.326
	**HGSOC (8 states)**	**0.600**	0.555	0.485	0.477	0.589	0.585	0.537	0.511
	**LUAD (3 states)**	0.866	0.847	0.872	0.881	0.864	0.868	0.809	**0.902**
	**cSCC (4 states)**	0.827	0.806	0.763	0.783	0.829	**0.832**	0.819	0.753
	**B, Mono-cytes, NK**	0.997	0.998	0.996	**0.999**	0.977	0.987	0.998	0.999
	**B cell subtypes**	**0.869**	0.667	0.362	0.104	0.331	0.243	0.334	0.758
	**CD4+ T cell subtypes**	**0.451**	0.319	0.340	0.008	0.020	0.053	0.009	0.157
	**CD8+ T cell subtypes**	**0.851**	0.753	0.756	0.455	0.389	0.371	0.453	0.417
**Informationquantity (logistic regression 10-fold CV)**	**BRCA (6 states)**	0.606 (0.067)	0.605 (0.045)	0.571 (0.045)	0.594 (0.066)	0.599 (0.057)	0.605 (0.058)	0.6 (0.066)	**0.629 (0.052)**
	**HGSOC (8 states)**	0.726 (0.029)	0.673 (0.027)	0.585 (0.038)	**0.727 (0.027)**	0.725 (0.03)	0.726 (0.029)	0.721 (0.031)	0.719 (0.024)
	**LUAD (3 states)**	0.909 (0.015)	0.912 (0.016)	0.908 (0.015)	0.903 (0.012)	0.904 (0.012)	0.904 (0.014)	0.917 (0.013)	**0.918 (0.014)**
	**cSCC (4 states)**	0.867 (0.033)	0.858 (0.038)	0.83 (0.035)	0.868 (0.03)	0.867 (0.034)	**0.87 (0.032)**	0.866 (0.034)	0.864 (0.031)
	**B, Mono-cytes, NK**	**1.0 (0.0)**	**1.0 (0.0)**	0.999 (0.001)	**1.0 (0.0)**	**1.0 (0.0)**	**1.0 (0.0)**	0.999 (0.001)	0.999 (0.0)
	**B cell subtypes**	0.916 (0.025)	0.794 (0.018)	0.563 (0.048)	0.903 (0.021)	**0.918 (0.024)**	0.916 (0.025)	0.915 (0.026)	0.899 (0.021)
	**CD4+ T cell subtypes**	0.808 (0.019)	0.672 (0.016)	0.61 (0.051)	0.782 (0.021)	0.806 (0.017)	**0.81 (0.016)**	0.803 (0.018)	0.793 (0.013)
	**CD8+ T cell subtypes**	**0.935 (0.014)**	0.871 (0.016)	0.846 (0.027)	0.934 (0.012)	0.931 (0.013)	0.934 (0.012)	0.934 (0.01)	0.93 (0.01)

Supplemental Table S8. The number of top-expressed genes across all cells in the PBMC dataset, together with the number of signature genes within each subset. Percentages indicate the proportion of genes in each subset relative to all genes (left column) and the proportion of marker genes within each subset relative to the total number of marker genes for the corresponding cell type.

**Top expressed genes across all cells**	**B cell marker genes within the subset**	**Monocyte marker within the subset**	**NK cell marker genes within the subset**
The last bin of 25 bins: 486 (4%)	96 (16.4%)	200 (39.1%)	77 (21.3%)
The last bin of 50 bins: 243 (2%)	44 (7.5%)	90 (17.6%)	33 (9.1%)
Control/2 with 100 control genes: 50 (0.4%)	8 (1.4%)	5 (1.0%)	3 (0.8%)
Control/2 with 200 control genes: 100 (0.8%)	17 (2.9%)	11 (2.2%)	6 (1.7%)

Supplemental Table S9. The number of top-expressed genes across all cells in the PBMC B cell dataset with three subtypes, together with the number of signature genes within each subset. Percentages indicate the proportion of genes in each subset relative to all genes (left column) and the proportion of marker genes within each subset relative to the total number of marker genes for the corresponding cell type.

**Top expressed genes across all cells**	**B intermediate cell marker genes removed**	**B memory cell marker genes removed**	**B naive cell marker genes removed**
The last bin of 25 bins: 451 (4%)	73 (57%)	82 (65.6%)	89 (71.8%)
The last bin of 50 bins: 226 (2%)	49 (38.3%)	62 (49.6%)	66 (53.2%)
Control/2 with 100 control genes: 50 (0.4%)	15 (11.7%)	25 (20%)	21 (16.9%)
Control/2 with 200 control genes: 100 (0.8%)	25 (19.5%)	44 (35.2%)	38 (30.6%)

Supplemental Table S10. The number of top-expressed genes across all cells in the PBMC B cell dataset with six subtypes, together with the number of signature genes within each subset. Percentages indicate the proportion of genes in each subset relative to all genes (left column) and the proportion of marker genes within each subset relative to the total number of marker genes for the corresponding cell type.

**Top expressed genes across all cells**	**B intermediate kappa cell marker genes within the subset**	**B memory kappa cell marker genes within the subset**	**B naive kappa cell marker genes within the subset**	**B intermediate lambda cell marker genes within the subset**	**B memory lambda cell marker genes within the subset**	**B naive lambda cell marker genes within the subset**
The last bin of 25 bins: 451 (4%)	70 (59.8%)	73 (64.6%)	81 (69.8%)	57 (53.3%)	79 (64.8%)	78 (73.6%)
The last bin of 50 bins: 226 (2%)	48 (41%)	54 (47.7%)	60 (51.7%)	37 (34.6%)	61 (50%)	57 (53.8%)
Control/2 with 100 control genes: 50 (0.4%)	15 (12.8%)	22 (19.5%)	17 (14.7%)	12 (11.2%)	25 (20.5%)	17 (16%)
Control/2 with 200 control genes: 100 (0.8%)	25 (21.4%)	37 (32.7%)	33 (28.4%)	16 (15%)	43 (35.2%)	32 (30.2%)

Supplemental Table S11. The number of top-expressed genes across all cells in the PBMC CD4+ T cell dataset with six subtypes, together with the number of signature genes within each subset. Percentages indicate the proportion of genes in each subset relative to all genes (left column) and the proportion of marker genes within each subset relative to the total number of marker genes for the corresponding cell type.

Top expressed genes across all cells	CD4+ CTL cell marker genes within the subset	CD4+ naive cell marker genes within the subset	Treg cell marker genes within the subset	CD4+ proliferating cell marker genes within the subset	CD4+ TCM cell marker genes within the subset	CD4+ TEM cell marker genes within the subset
The last bin of 25 bins: 436 (4%)	23 (51.1%)	106 (90.6%)	97 (95.1%)	113 (9.6%)	117 (77.5%)	91 (79.8%)
The last bin of 50 bins: 218 (2%)	16 (35.6%)	97 (82.9%)	93 (91.2%)	52 (4.4%)	97 (64.2%)	84 (73.7%)
Control/2 with 100 control genes: 50 (0.46%)	2 (4.4%)	42 (35.9%)	43 (42.2%)	3 (0.3%)	42 (27.8%)	38 (33.3%)
Control/2 with 200 control genes: 100 (0.9%)	4 (8.9%)	78 (66.7%)	77 (75.5%)	8 (0.7%)	69 (45.7%)	61 (53.5%)

Supplemental Table S12. The number of top-expressed genes across all cells in the PBMC CD8+ T cell dataset with four subtypes, together with the number of signature genes within each subset. Percentages indicate the proportion of genes in each subset relative to all genes (left column) and the proportion of marker genes within each subset relative to the total number of marker genes for the corresponding cell type.

**Top expressed genes across all cells**	**CD8+ naive cell marker genes within the subset**	**CD8+ proliferating cell marker genes within the subset**	**CD8+ TCM cell marker genes within the subset**	**CD8+ TEM cell marker genes within the subset**
The last bin of 25 bins: 446 (4%)	119 (86.9%)	103 (20.2%)	97 (93.3%)	59 (52.2%)
The last bin of 50 bins: 223 (2%)	103 (75.2%)	47 (9.2%)	89 (85.6%)	35 (31%)
Control/2 with 100 control genes: 50 (0.45%)	39 (28.5%)	4 (0.8%)	38 (36.5%)	6 (5.3%)
Control/2 with 200 control genes: 100 (0.9%)	79 (575.5%)	6 (1.2%)	70 (67.3%)	10 (8.8%)

Supplemental Table S13. Full ESCC and LUAD cancer EMT signature genes and per gene information: ESCC- and LUAD-specific cancer EMT signature. For each gene, the table indicates if it has been included in any considered pan-cancer EMT signature, in EMTome (Vasaikar et al. 2021), in dbEMT (Zhao et al. 2019), or if we have found literature associating the gene with EMT. **See separate file linked to the article: See separate file linked to the article: SupplTableS13.xlsx**

Supplemental Table S14. AUCPRC values quantifying the performance of signature scores to discriminate malignant EMT cells, CAFs, malignant cells not undergoing EMT, and all other cell types using the LUAD- and ESCC-specific cancer EMT and the pan-cancer EMT signatures for scoring.

	**AUCPRC, cancer MES-like *vs.* CAFs**				**AUCPRC, cancer MES-like *vs.* other cancer cells**				**AUCPRC, cancer MES-like *vs.* rest**			
	**ESCC**	**LUAD**	**CRC**	**BRCA**	**ESCC**	**LUAD**	**CRC**	**BRCA**	**ESCC**	**LUAD**	**CRC**	**BRCA**
**ESCC and LUAD cancer EMT signature (this paper)**	**0.952**	**0.988**	**0.987**	**0.907**	0.595	0.545	0.705	0.702	**0.592**	**0.513**	0.701	**0.511**
**Hallmark EMT signature** (Liberzon et al. 2015)	0.065	0.333	0.442	0.460	**0.684**	**0.596**	0.631	**0.860**	0.038	0.049	0.042	0.172
**pEMT gene module** (Barkley et al. 2022)	0.428	0.523	0.651	0.743	0.520	0.474	0.563	0.708	0.332	0.185	0.277	0.437
**EMT signature Foroutan *et al.*** (Foroutan et al. 2017)	0.065	0.382	0.445	0.459	0.368	0.451	**0.850**	0.825	0.033	0.128	0.190	0.194
**EMT signature Groeger *et al.*** (Gröger et al. 2012)	0.065	0.333	0.442	0.456	0.607	0.413	0.224	0.744	0.024	0.014	0.019	0.115
**EMT signature Hollern *et al.*** (Hollern et al. 2018)	0.070	0.589	0.880	0.472	0.104	0.581	0.795	0.600	0.028	0.245	**0.707**	0.106
**EMT signature *Mak et al.*** (Mak et al. 2016)	0.065	0.334	0.442	0.463	0.418	0.514	0.566	0.794	0.026	0.067	0.085	0.182

	**AUCPRC, cancer MES-like *vs.* CAFs**				**AUCPRC, cancer MES-like *vs.* other cancer cells**				**AUCPRC, cancer MES-like *vs.* rest**			
	**ESCC**	**LUAD**	**CRC**	**BRCA**	**ESCC**	**LUAD**	**CRC**	**BRCA**	**ESCC**	**LUAD**	**CRC**	**BRCA**
**EMT signature Tan *et al.*** (Tan et al. 2014)	0.065	0.411	0.813	0.454	0.137	0.520	0.807	0.610	0.031	0.218	0.688	0.196

Supplemental Table S15. Association of ESCC- and LUAD_Xing-specific cancer EMT signature scores and histological subtypes in TCGA (only cancer types with at least 1 histotype are included).

Cancer type	P-value	P-value adjusted
ACC	0.4982124	0.6042173
BRCA	0.0023264	0.0062037
CESC	0.0009604	0.0028811
CHOL	0.8403236	0.8768594
COAD	0.5035145	0.6042173
DLBC	0.2726163	0.4361862
ESCA	0.0000778	0.0004667
GBM	0.3655240	0.5105443
HNSC	0.0000997	0.0004785
LGG	0.0563420	0.1229281
LIHC	0.0005637	0.0022546
MESO	0.3401511	0.5102267
PAAD	0.0009509	0.0028811
PCPG	0.9400718	0.9400718
PRAD	0.3829082	0.5105443
READ	0.6280988	0.7178272
SARC	0.0937034	0.1729909
STAD	0.0150783	0.0361880
TGCT	0.0000007	0.0000054
THCA	4.6427233E-033	1.1142536E-031
THYM	6.5542819E-09	7.8651383E-08
UCEC	0.7154815	0.7805253
UCS	0.0658779	0.1317558

Cancer type	P-value	P-value adjusted
UVM	0.2092861	0.3587762

Supplemental Figures

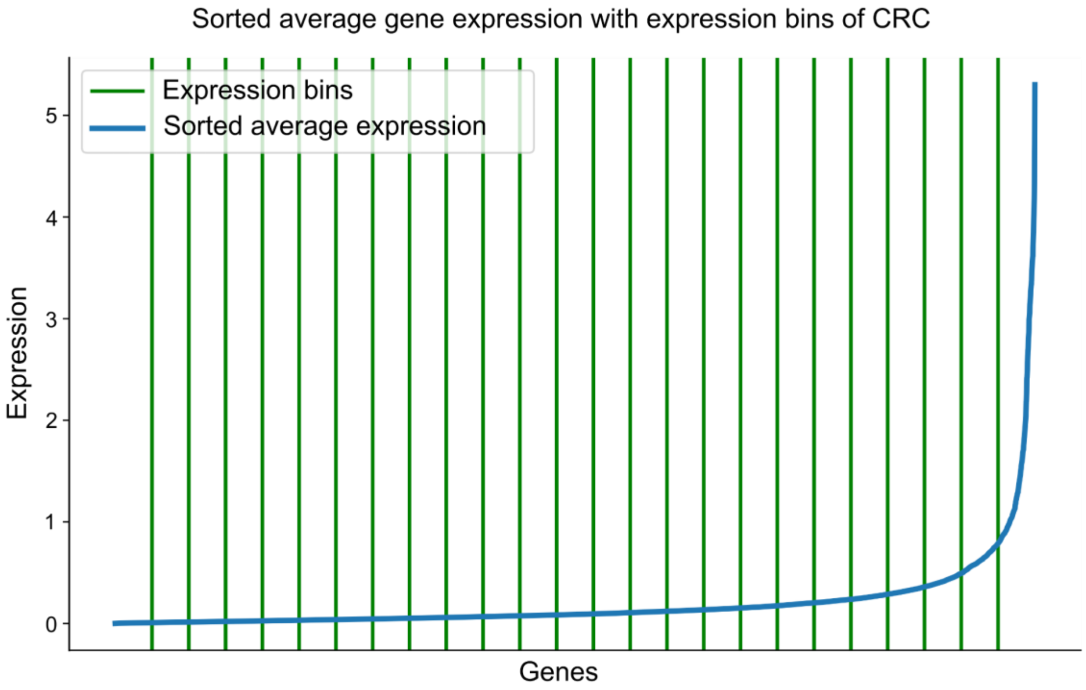

Supplemental Fig. S1: Sorted average gene expression values of CRC with expression bins for Tirosh *et al.* (Tirosh et al. 2016) based gene signature scoring methods.

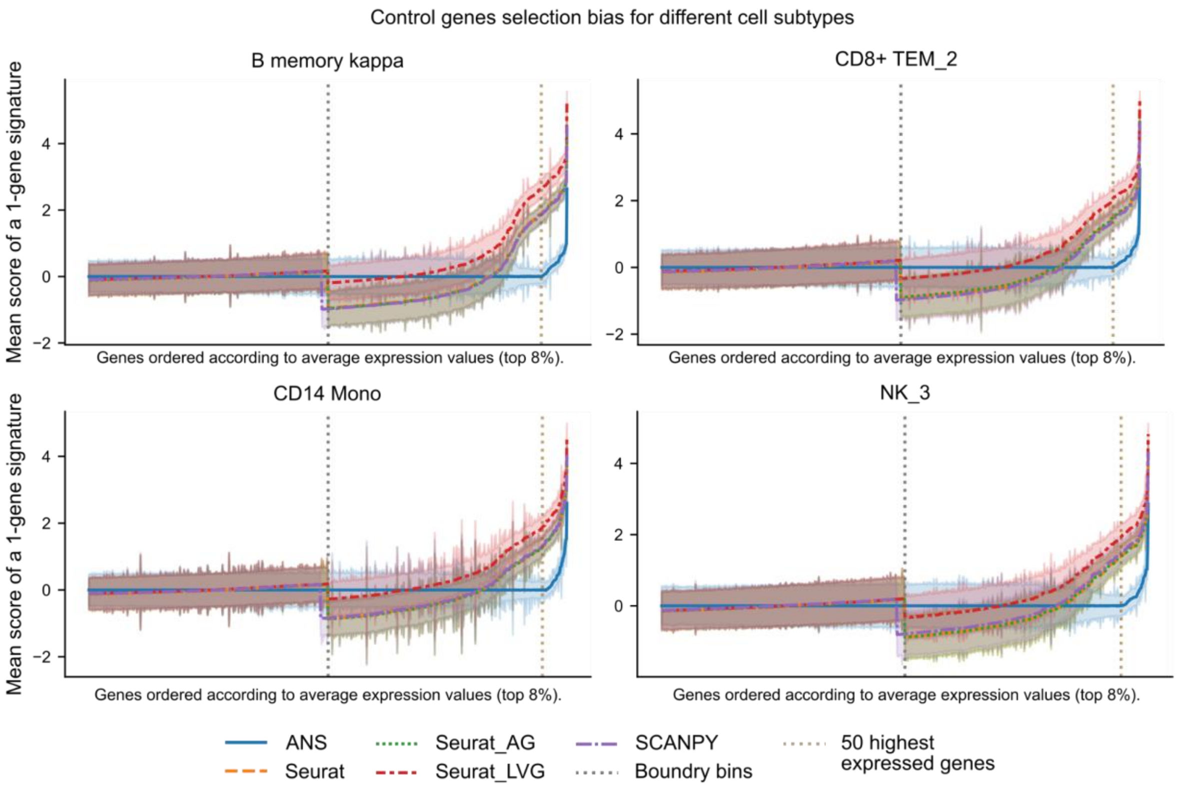

Supplemental Fig. S2: Control genes selection bias in Tirosh-based methods for the top 8% of highly expressed genes in B memory kappa, CD8+ TEM 2, CD14+ Monocytes, and NK 3 cells within the PBMC dataset. The x-axis illustrates genes sorted by their average expression levels, while the y-axis depicts the mean and standard deviation of the scores across all cells for a single-gene signature. Vertical dashed lines indicate the expression bin boundary and the top 50 highly expressed genes. The bias of a scoring method is indicated by how far the mean score of a gene deviates from zero.

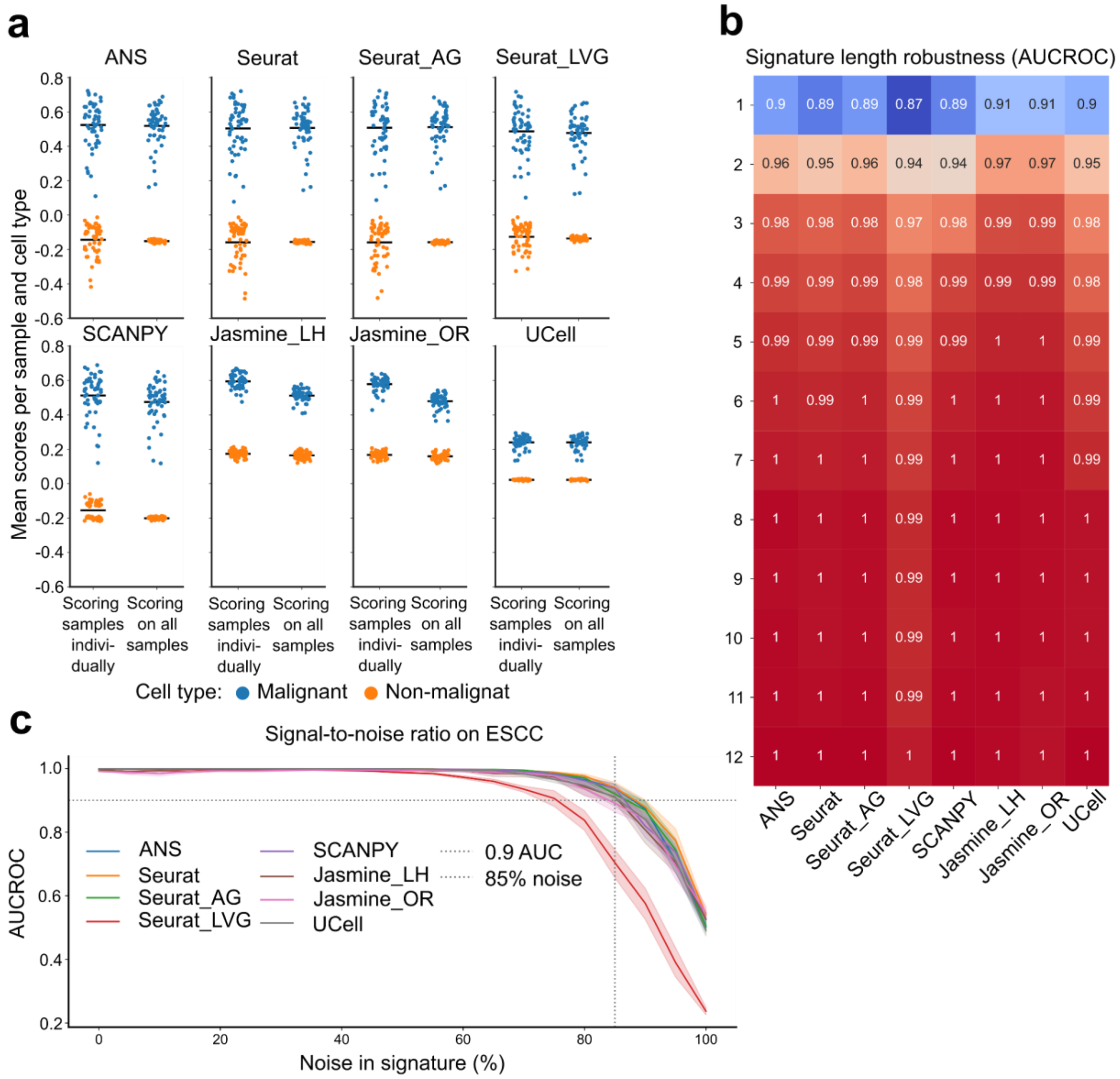

Supplemental Fig. S3: Benchmark on ESCC **a.** The influence of dataset composition on scoring ESCC cells using a 100-gene signature associated with malignant cells. Each dot represents the mean score for all cells within a sample, grouped by malignancy (malignant in blue or non-malignant in orange) and scoring mode (scoring all the samples together or individually). A black horizontal bar represents the mean value of all dots within each group. We observed a decrease in score variance when scoring on all samples. **b.** The minimum required signature length per scoring method for perfect classification (AUCROC of 1) when discriminating between malignant and non-malignant cell scores in ESCC. **c.** The robustness to noise in a signature when discriminating between malignant and non-malignant cells in ESCC. Starting with a 100-gene signature, we performed iterative replacements of genes with random genes that exhibited a $|\log_2 FC| < 0.5$ and an adjusted p-value > 0.1 during differential gene expression (DGEX) analysis between malignant and non-malignant cells. The standard variation was calculated from 20 simulation runs.

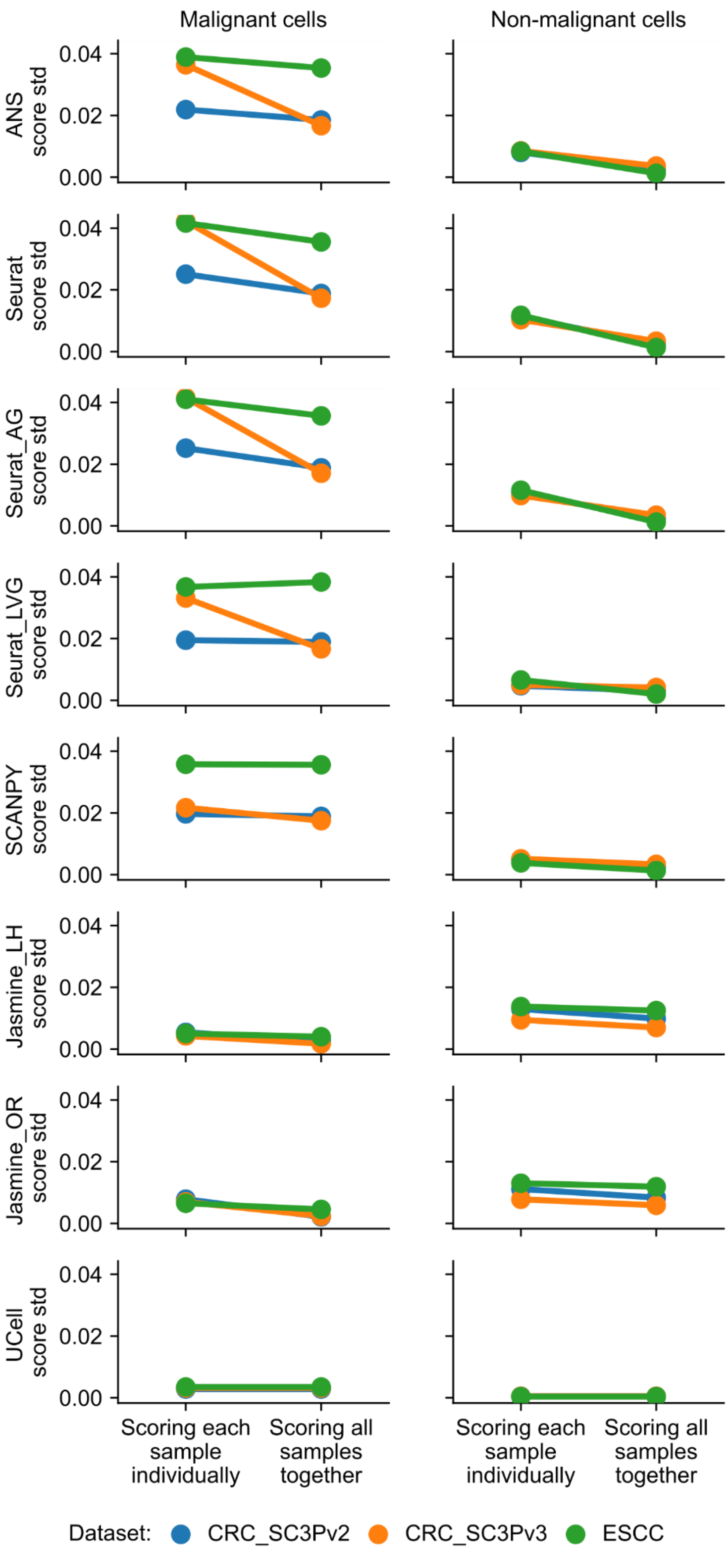

Supplemental Fig. S4: Score variances comparison between scoring modes (scoring sample individually versus scoring the entire dataset) for all scoring methods and two malignancy types on CRC and ESCC.

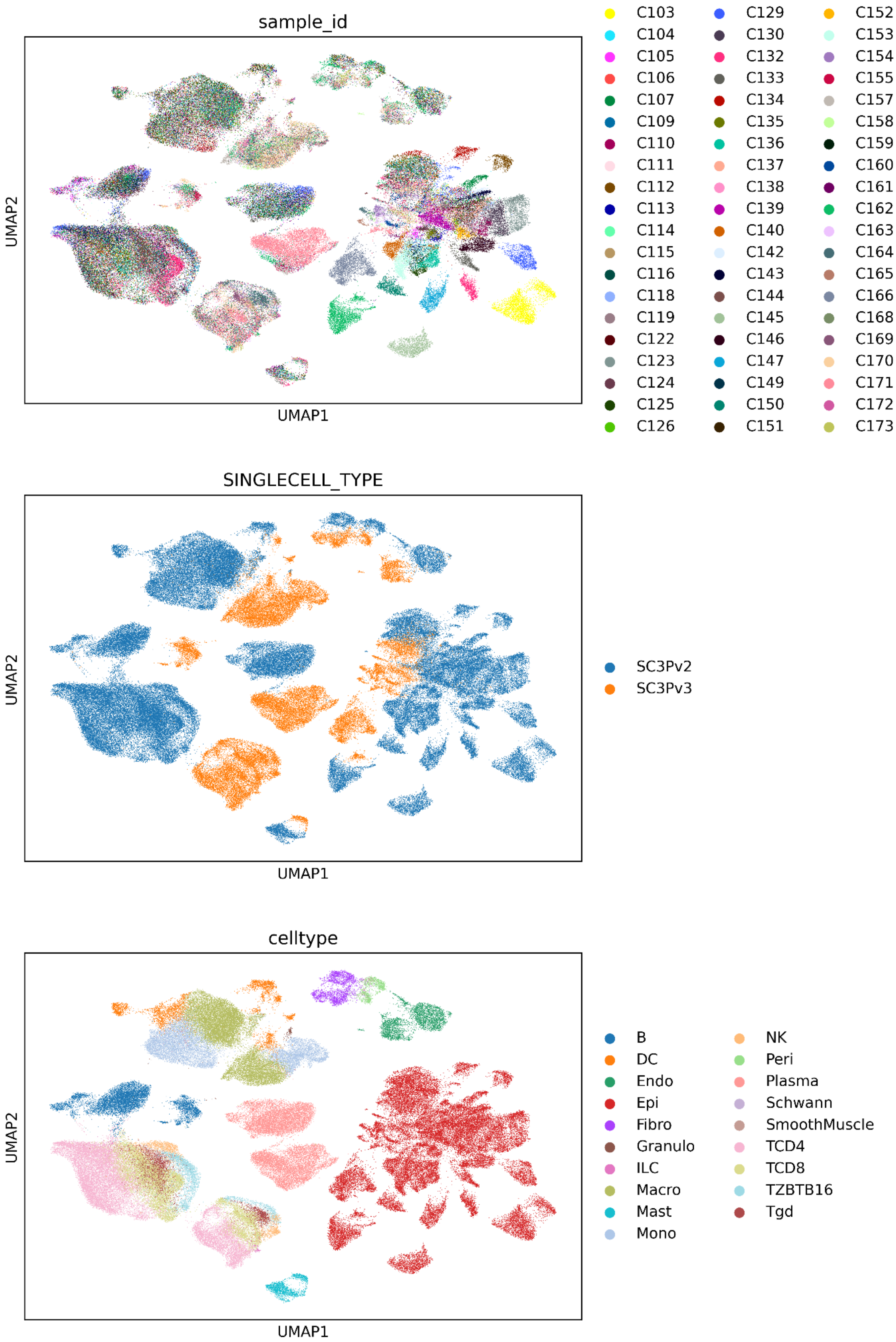

Supplemental Fig. S5: UMAP representation of the cell types in the CRC dataset. Cells in the respective panels are colored by sample id, chemistry type used and cell type. Epi cells on the bottom panel represent the malignant cells.

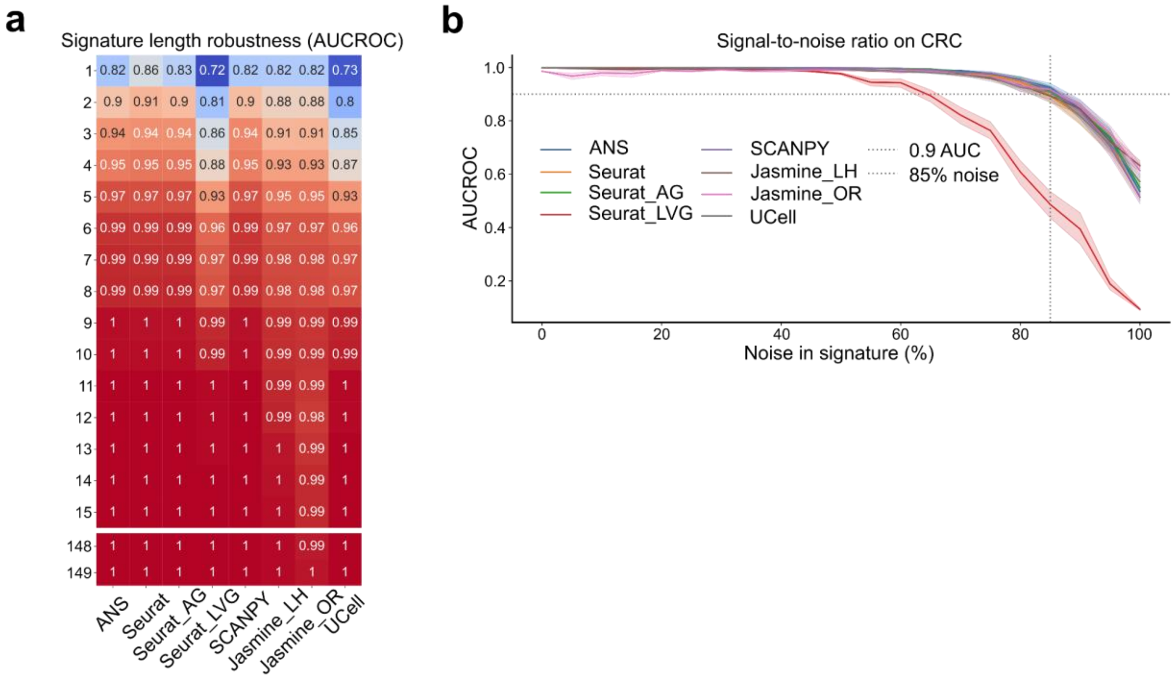

Supplemental Fig. S6: Sensitivity to signature length and signal-to-noise ratio in CRC. **a.** The minimum required signature length per scoring method for perfect classification (AUCROC of 1) when discriminating between malignant and non-malignant cell scores in CRC. **b.** The robustness to noise in a signature when discriminating between malignant and non-malignant cells in CRC. Starting with a 100-gene signature, we performed iterative replacements of genes with random genes that exhibited a $|\log_2 FC| < 0.5$ and an adjusted p-value > 0.1 during differential gene expression (DGEX) analysis between malignant and non-malignant cells. The standard variation was calculated from 20 simulation runs.

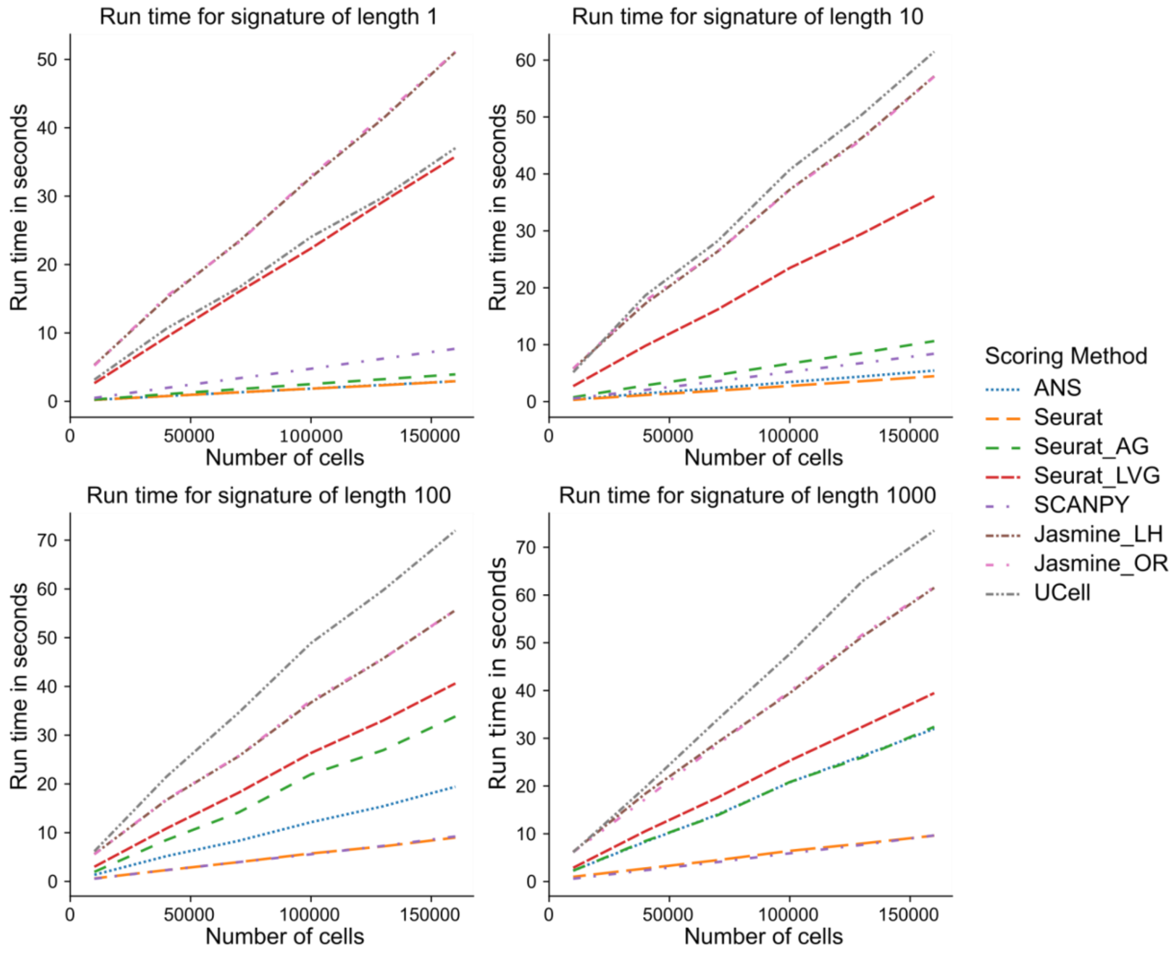

Supplemental Fig. S7: Benchmarking computation times of scoring methods. This figure presents the computation times required for scoring across varying dataset sizes, with four distinct signature lengths (1, 10, 100, 1000).

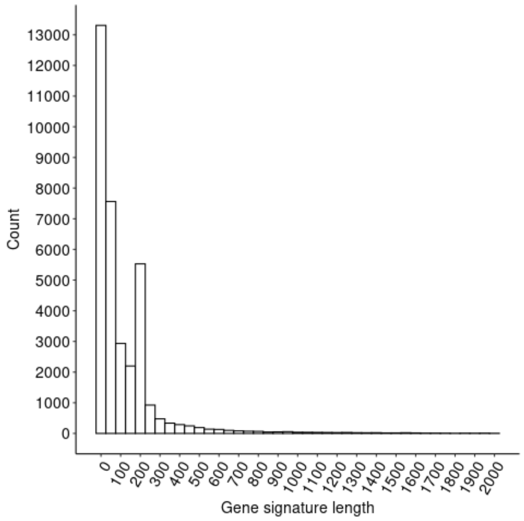

Supplemental Fig. S8: The distribution of gene signature length across 35,134 gene signatures from MsigDB human collection.

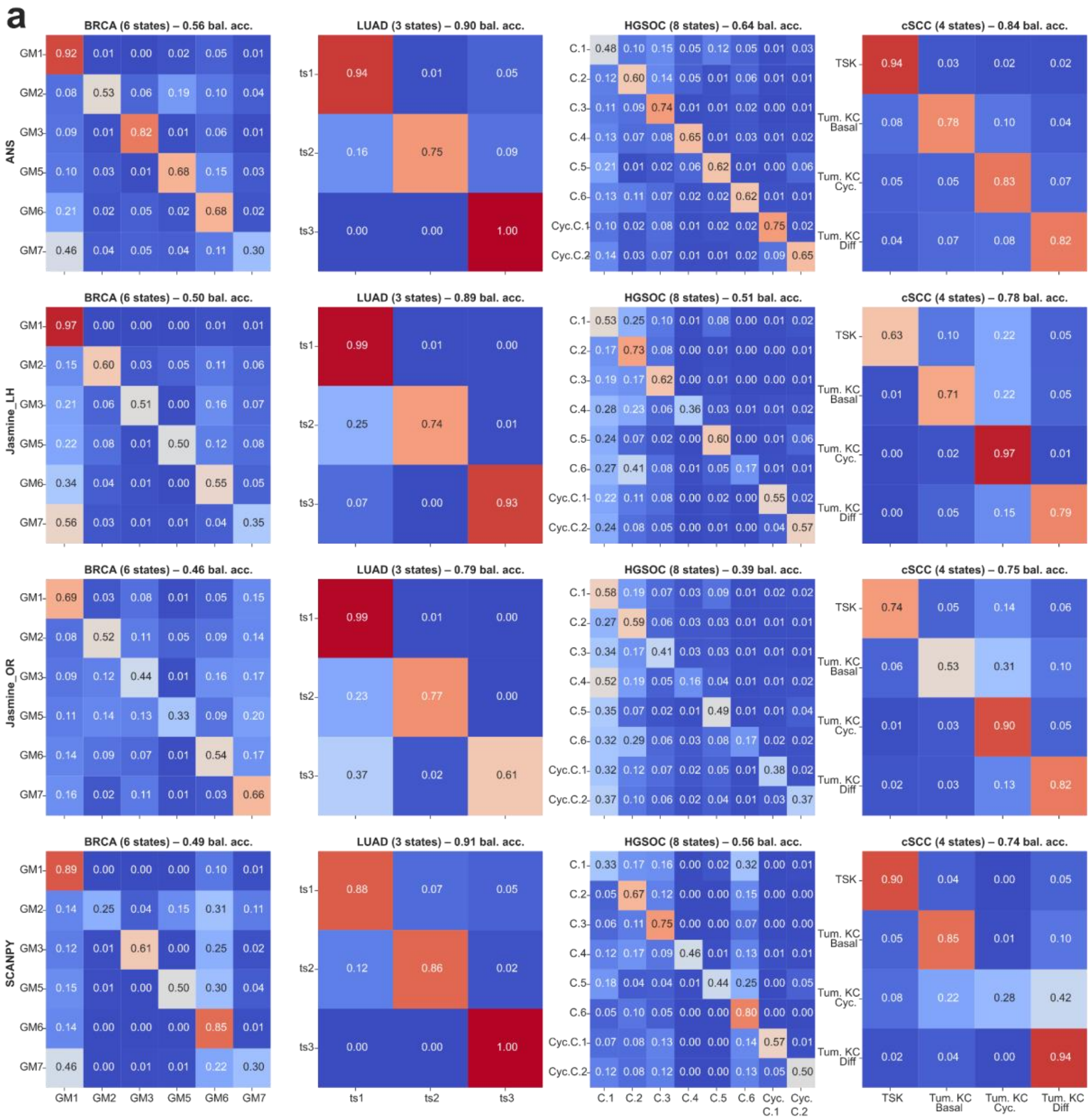
a
BRCA (6 states) – 0.56 bal. acc.
LUAD (3 states) – 0.90 bal. acc.
HGSOC (8 states) – 0.64 bal. acc.
cSCC (4 states) – 0.84 bal. acc.
ANS
BRCA (6 states) – 0.50 bal. acc.
LUAD (3 states) – 0.89 bal. acc.
HGSOC (8 states) – 0.51 bal. acc.
cSCC (4 states) – 0.78 bal. acc.
Jasmine_LH
BRCA (6 states) – 0.46 bal. acc.
LUAD (3 states) – 0.79 bal. acc.
HGSOC (8 states) – 0.39 bal. acc.
cSCC (4 states) – 0.75 bal. acc.
Jasmine_OR
BRCA (6 states) – 0.49 bal. acc.
LUAD (3 states) – 0.91 bal. acc.
HGSOC (8 states) – 0.56 bal. acc.
cSCC (4 states) – 0.74 bal. acc.
SCANPY
GM1 GM2 GM3 GM5 GM6 GM7
ts1 ts2 ts3
C.1 C.2 C.3 C.4 C.5 C.6 Cyc.C.1 Cyc.C.2
TSK Tum. KC Basal Tum. KC Cyc. Tum. KC Diff

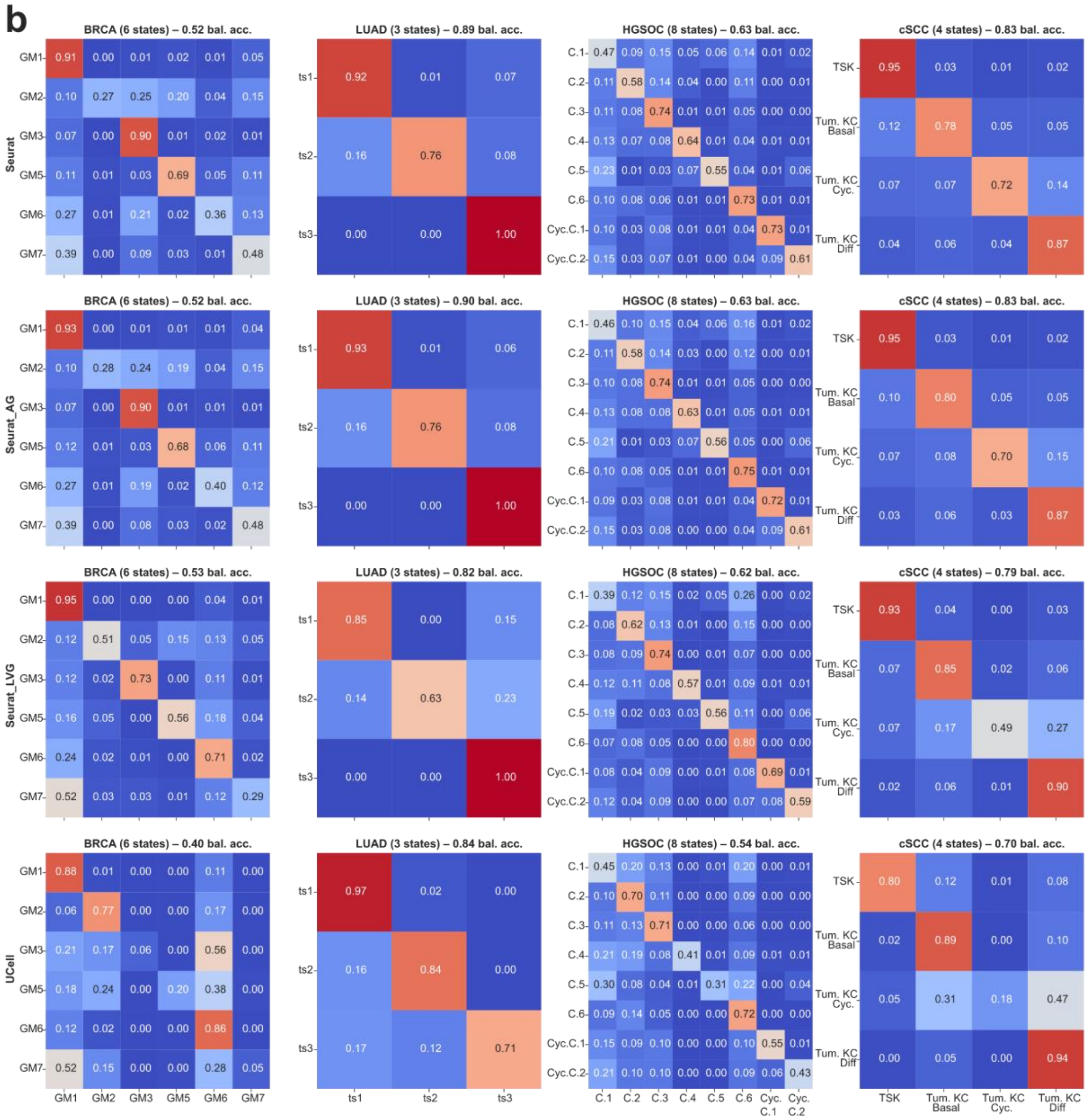
b
BRCA (6 states) – 0.52 bal. acc.
LUAD (3 states) – 0.89 bal. acc.
HGSOC (8 states) – 0.63 bal. acc.
cSCC (4 states) – 0.83 bal. acc.
Seurat
BRCA (6 states) – 0.52 bal. acc.
LUAD (3 states) – 0.90 bal. acc.
HGSOC (8 states) – 0.63 bal. acc.
cSCC (4 states) – 0.83 bal. acc.
Seurat_AG
BRCA (6 states) – 0.53 bal. acc.
LUAD (3 states) – 0.82 bal. acc.
HGSOC (8 states) – 0.62 bal. acc.
cSCC (4 states) – 0.79 bal. acc.
Seurat_LVG
BRCA (6 states) – 0.40 bal. acc.
LUAD (3 states) – 0.84 bal. acc.
HGSOC (8 states) – 0.54 bal. acc.
cSCC (4 states) – 0.70 bal. acc.
UCell
GM1
GM2
GM3
GM5
GM6
GM7
ts1
ts2
ts3
C.1
C.2
C.3
C.4
C.5
C.6
Cyc.C.1
Cyc.C.2
TSK
Tum. KC Basal
Tum. KC Cyc.
Tum. KC Diff

c

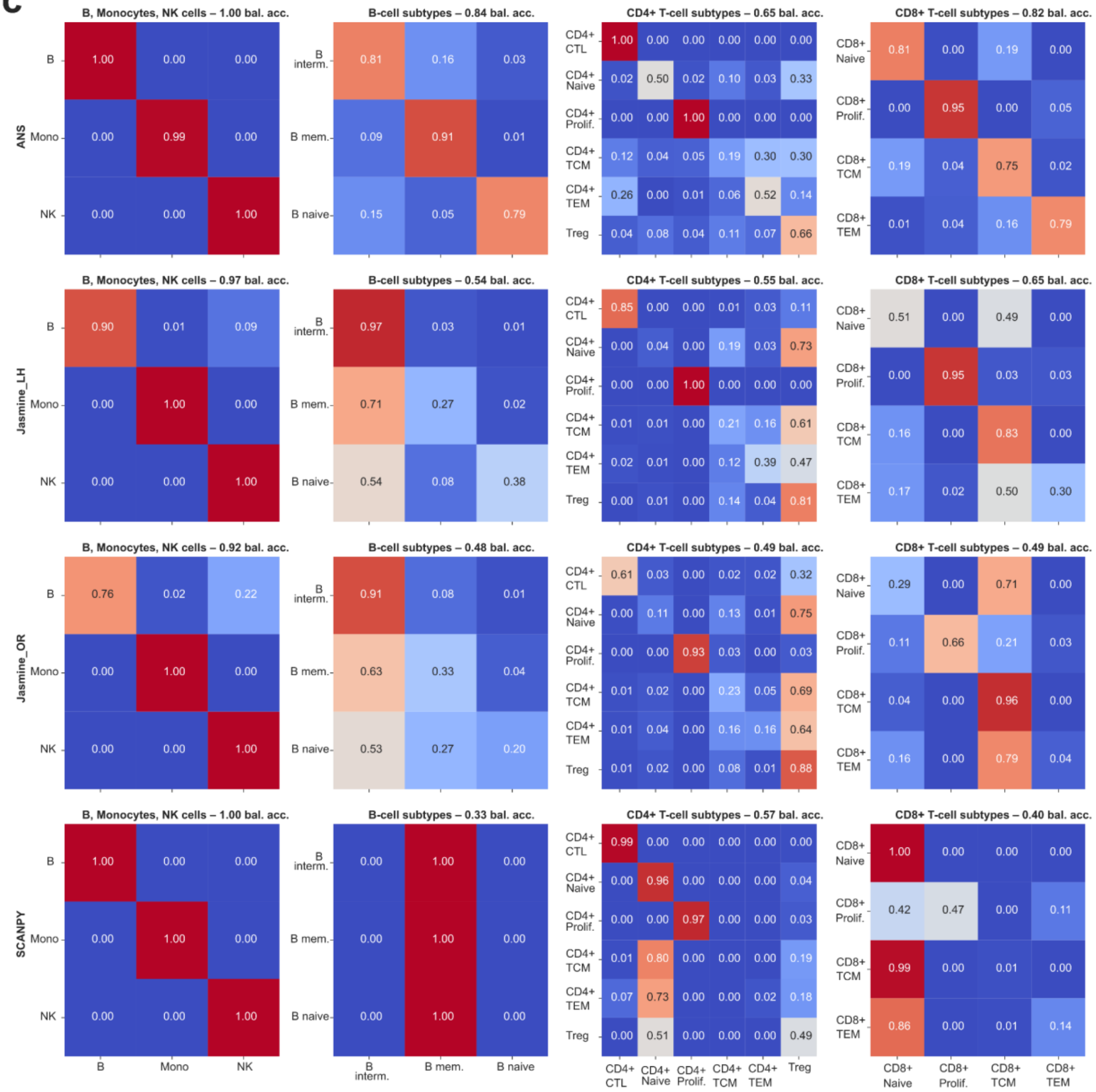
B, Monocytes, NK cells – 1.00 bal. acc.
B-cell subtypes – 0.84 bal. acc.
CD4+ T-cell subtypes – 0.65 bal. acc.
CD8+ T-cell subtypes – 0.82 bal. acc.
B, Monocytes, NK cells – 0.97 bal. acc.
B-cell subtypes – 0.54 bal. acc.
CD4+ T-cell subtypes – 0.55 bal. acc.
CD8+ T-cell subtypes – 0.65 bal. acc.
B, Monocytes, NK cells – 0.92 bal. acc.
B-cell subtypes – 0.48 bal. acc.
CD4+ T-cell subtypes – 0.49 bal. acc.
CD8+ T-cell subtypes – 0.49 bal. acc.
B, Monocytes, NK cells – 1.00 bal. acc.
B-cell subtypes – 0.33 bal. acc.
CD4+ T-cell subtypes – 0.57 bal. acc.
CD8+ T-cell subtypes – 0.40 bal. acc.
ANS
Jasmine_LH
Jasmine_OR
SCANPY
B
Mono
NK
B interm.
B mem.
B naive
CD4+ CTL
CD4+ Naive
CD4+ Prolif.
CD4+ TCM
CD4+ TEM
Treg
CD8+ Naive
CD8+ Prolif.
CD8+ TCM
CD8+ TEM

d

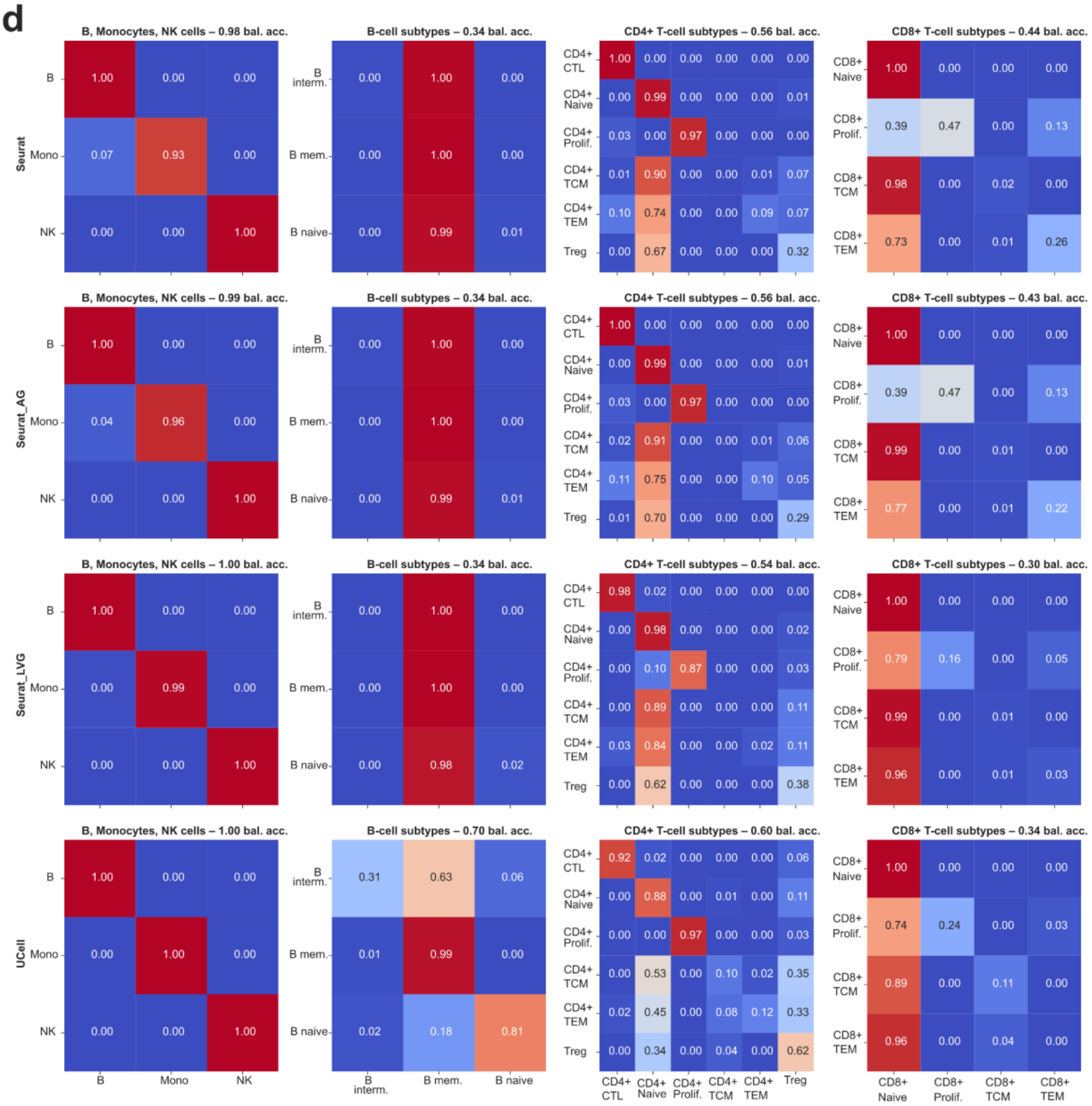

Supplemental Fig. S9: Confusion matrices for cell type/state prediction across multiple methods and datasets. **a**, Comparison of prediction performance between ANS, Jasmine_LH, Jasmine_OR, and SCANPY for cancer datasets (BRCA, LUAD, HGSOC, cSCC). **b**, Results for Seurat, Seurat_AG, Seurat_LVG, and UCell on the same cancer datasets. **c-d**, Performance evaluation across immune cell subsets using the same methods, showing B/Monocytes/NK cells, B cell subtypes, CD4+ T cell subtypes, and CD8+ T cell subtypes. Each matrix shows the relationship between true (y-axis) and predicted (x-axis) cell states, with values summing to one per row. Color scale ranges from blue (0) to red (1), with balanced accuracy (bal. acc.) shown for each matrix. **Non-overlapping** gene signatures were used to score cell states/types in each dataset.

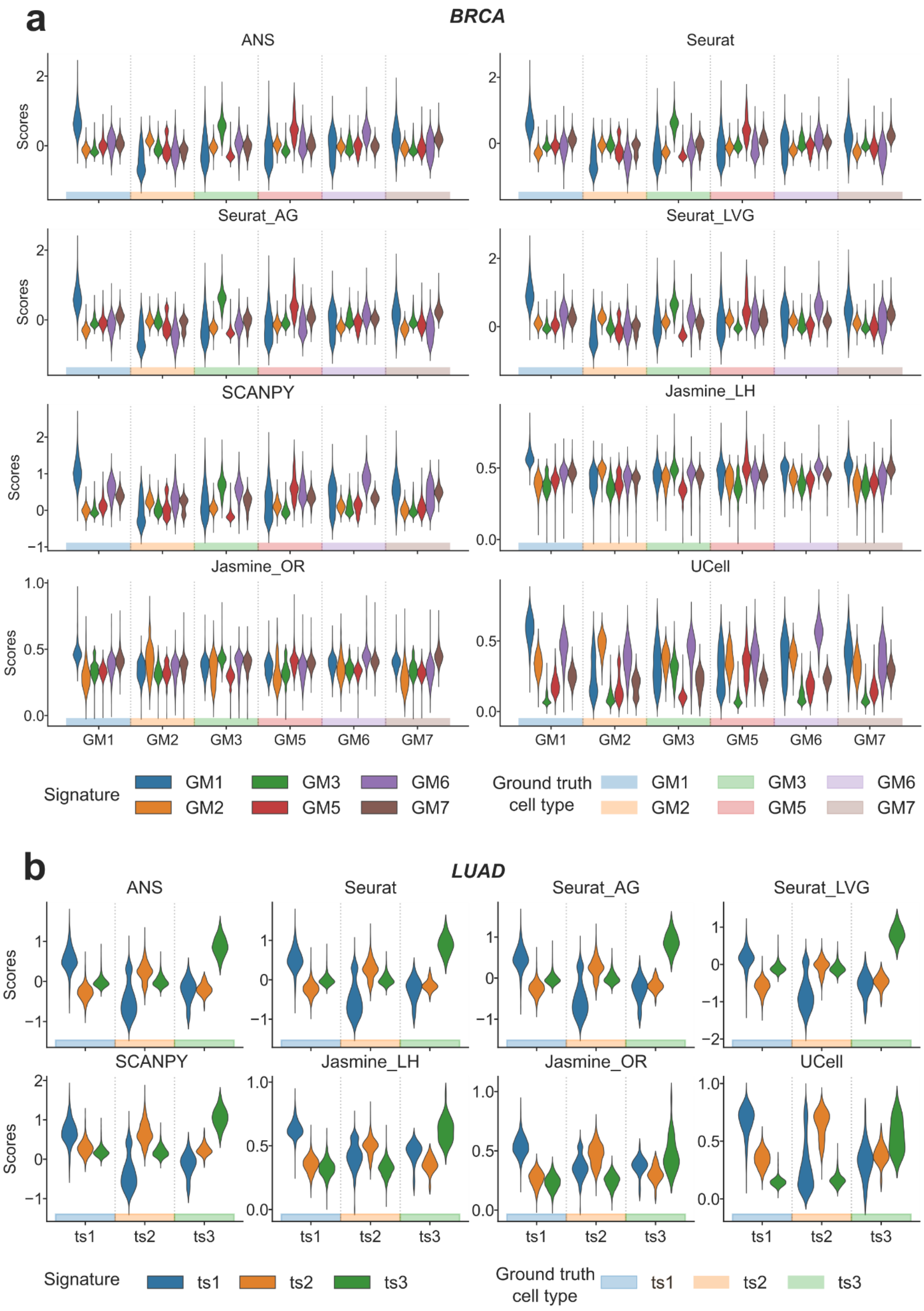
a
BRCA
ANS
Seurat
Seurat_AG
Seurat_LVG
SCANPY
Jasmine_LH
Jasmine_OR
UCell
Scores
GM1
GM2
GM3
GM5
GM6
GM7
Signature
Ground truth cell type
b
LUAD
ANS
Seurat
Seurat_AG
Seurat_LVG
SCANPY
Jasmine_LH
Jasmine_OR
UCell
Scores
ts1
ts2
ts3
Signature
Ground truth cell type

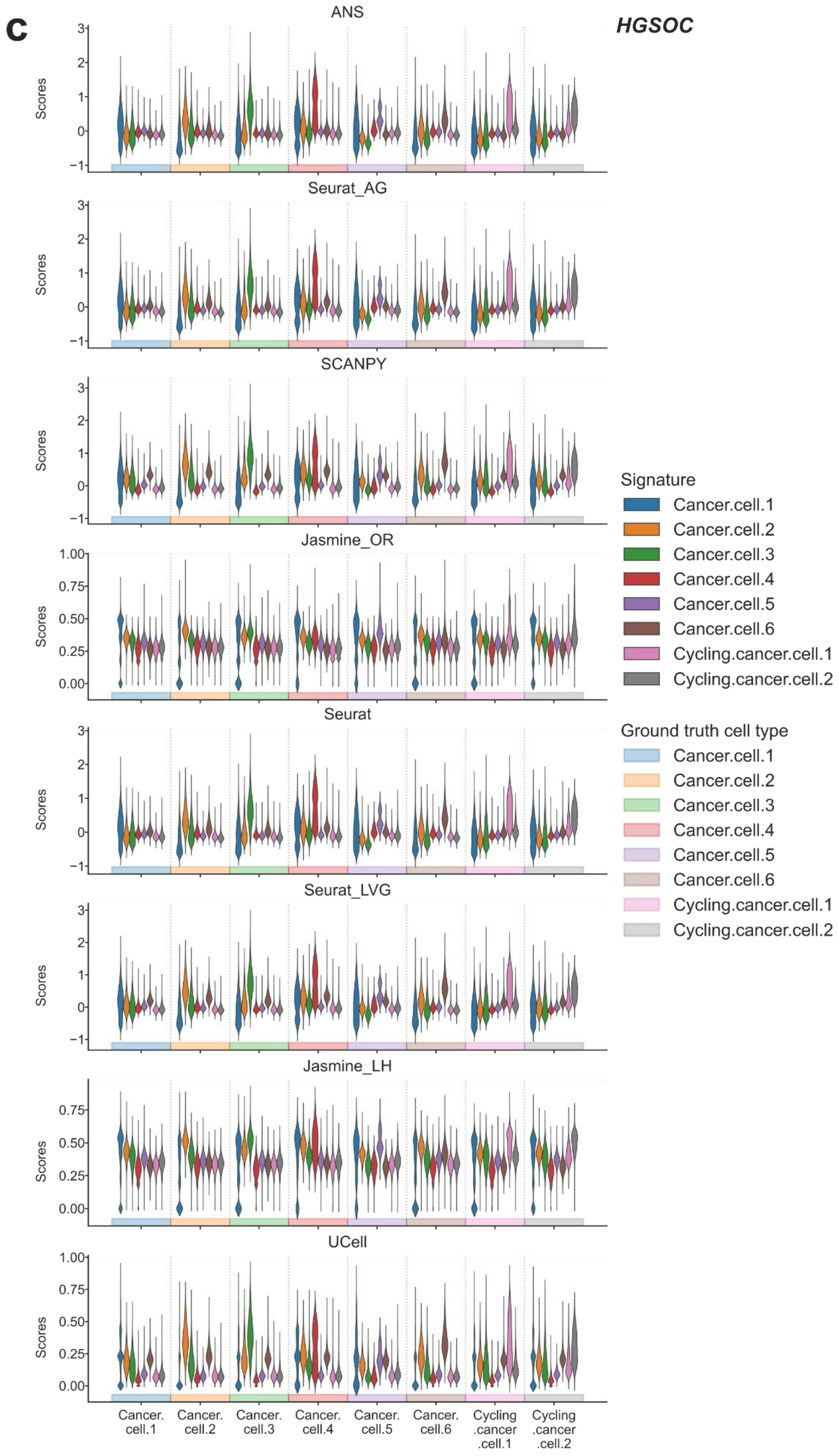
c
HGSOC
ANS
Seurat_AG
SCANPY
Jasmine_OR
Seurat
Seurat_LVG
Jasmine_LH
UCell
Scores
Signature
Cancer.cell.1
Cancer.cell.2
Cancer.cell.3
Cancer.cell.4
Cancer.cell.5
Cancer.cell.6
Cycling.cancer.cell.1
Cycling.cancer.cell.2
Ground truth cell type
Cancer.cell.1
Cancer.cell.2
Cancer.cell.3
Cancer.cell.4
Cancer.cell.5
Cancer.cell.6
Cycling.cancer.cell.1
Cycling.cancer.cell.2
Cancer.cell.1
Cancer.cell.2
Cancer.cell.3
Cancer.cell.4
Cancer.cell.5
Cancer.cell.6
Cycling.cancer.cell.1
Cycling.cancer.cell.2

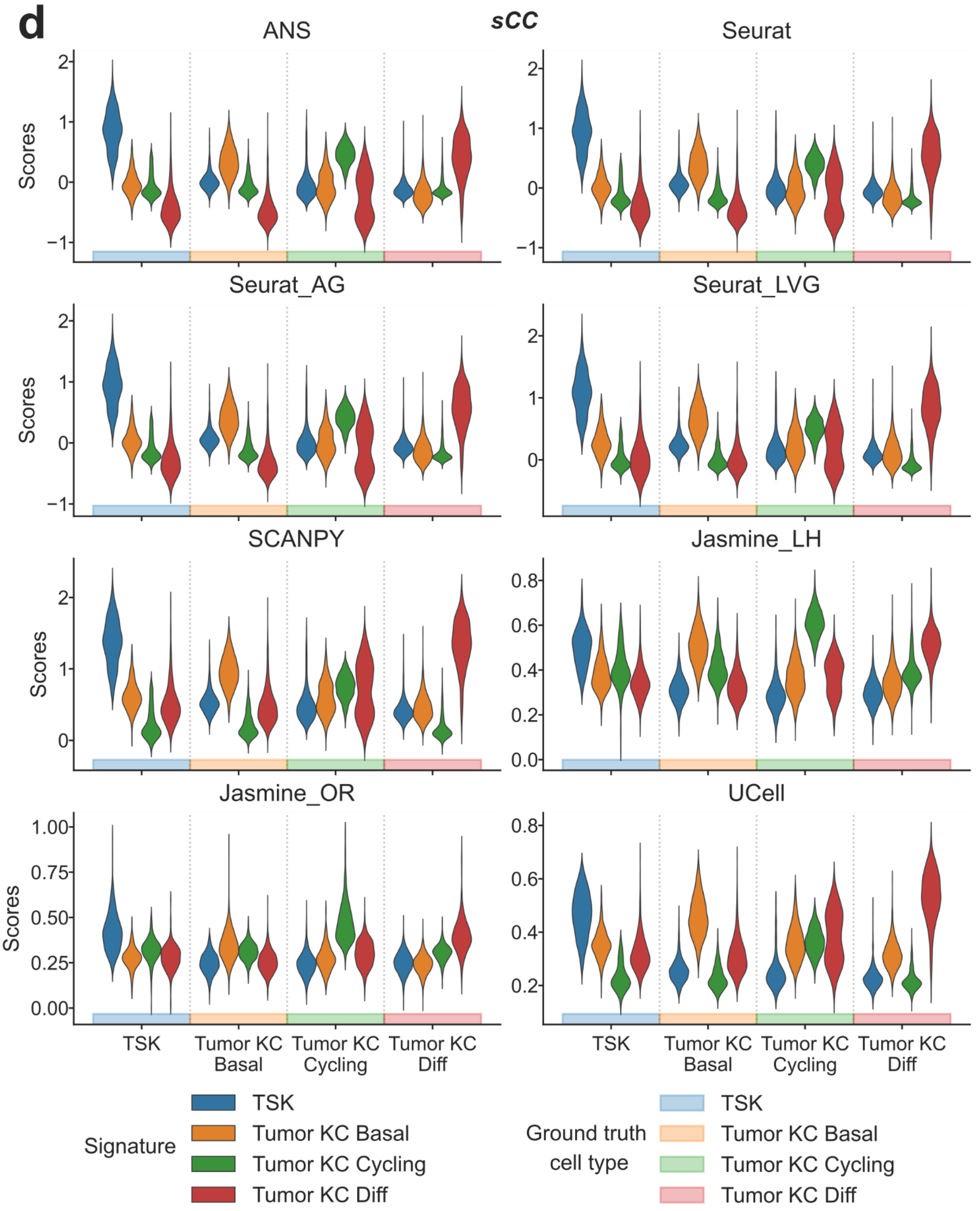
d
sCC
ANS
Seurat
Seurat_AG
Seurat_LVG
SCANPY
Jasmine_LH
Jasmine_OR
UCell
Scores
TSK
Tumor KC Basal
Tumor KC Cycling
Tumor KC Diff
Signature
Ground truth cell type

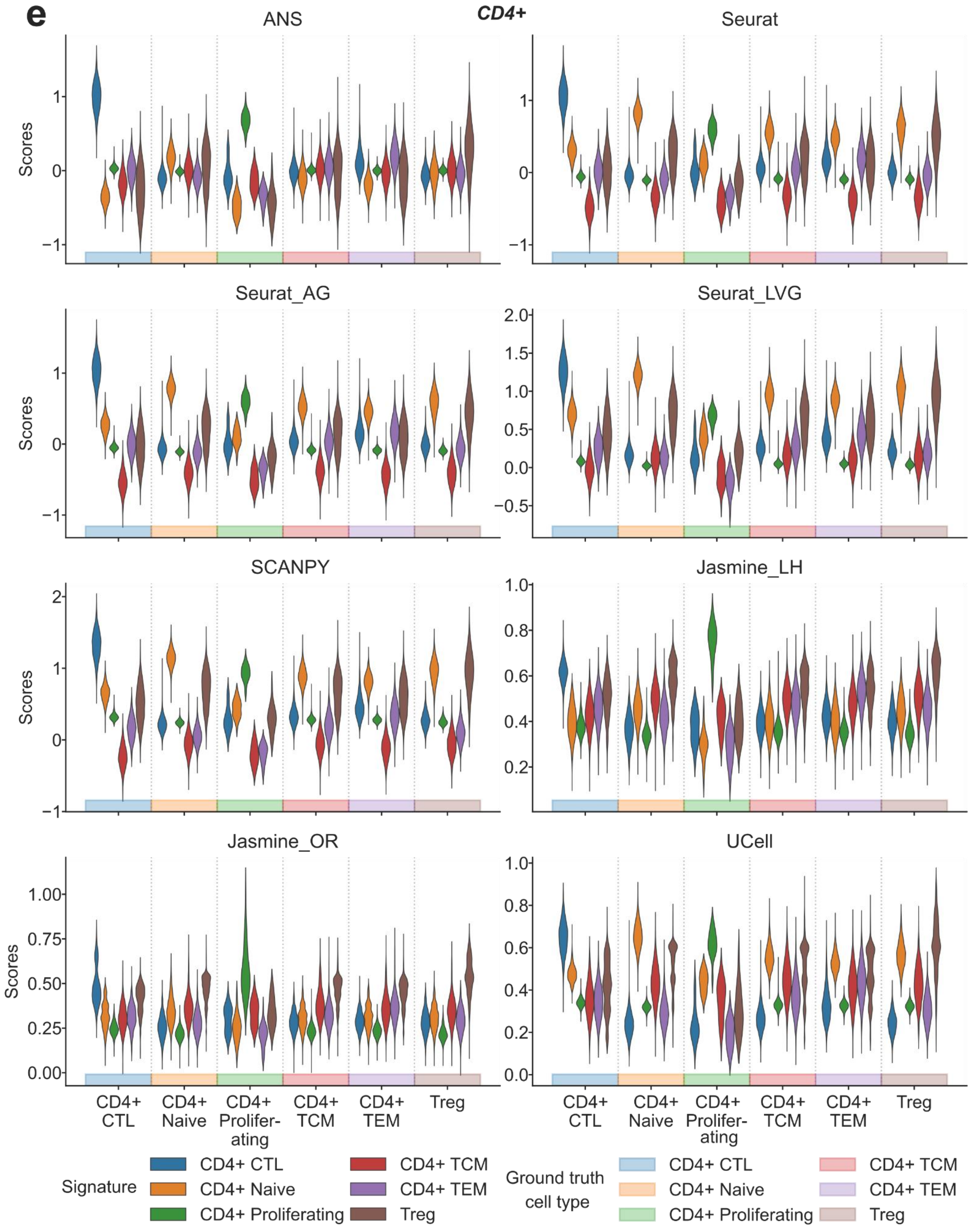
e
CD4+
ANS
Seurat
Seurat_AG
Seurat_LVG
SCANPY
Jasmine_LH
Jasmine_OR
UCell
Scores
CD4+ CTL
CD4+ Naive
CD4+ Prolifer-ating
CD4+ TCM
CD4+ TEM
Treg
Signature
CD4+ CTL
CD4+ Naive
CD4+ Proliferating
CD4+ TCM
CD4+ TEM
Treg
Ground truth cell type
CD4+ CTL
CD4+ Naive
CD4+ Proliferating
CD4+ TCM
CD4+ TEM
Treg

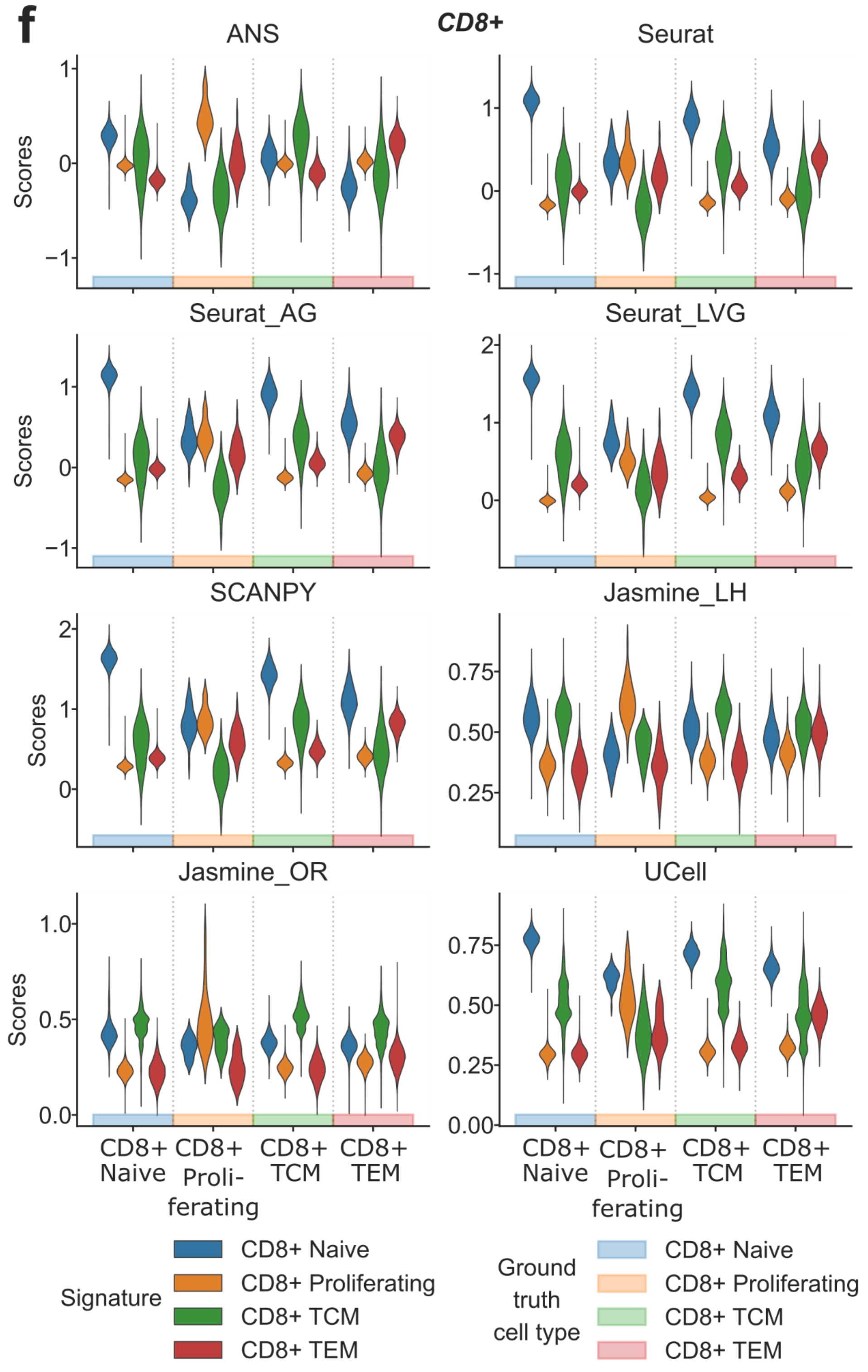

Supplemental Fig. S10: Violin plots showing the distribution of scores for each scored signature per dataset. For each cell state or type, we expect the highest scores for the signature associated with this state or type (matching dark and light shades). **a**, Breast cancer (BRCA). **b**, Lung adenocarcinoma (LUAD). **c**, High-grade serous ovarian cancer (HGSOC). **d**, Squamous cell carcinoma (SCC). **e**, CD4+ T cell subtypes. **f**, CD8+ T cell subtypes.

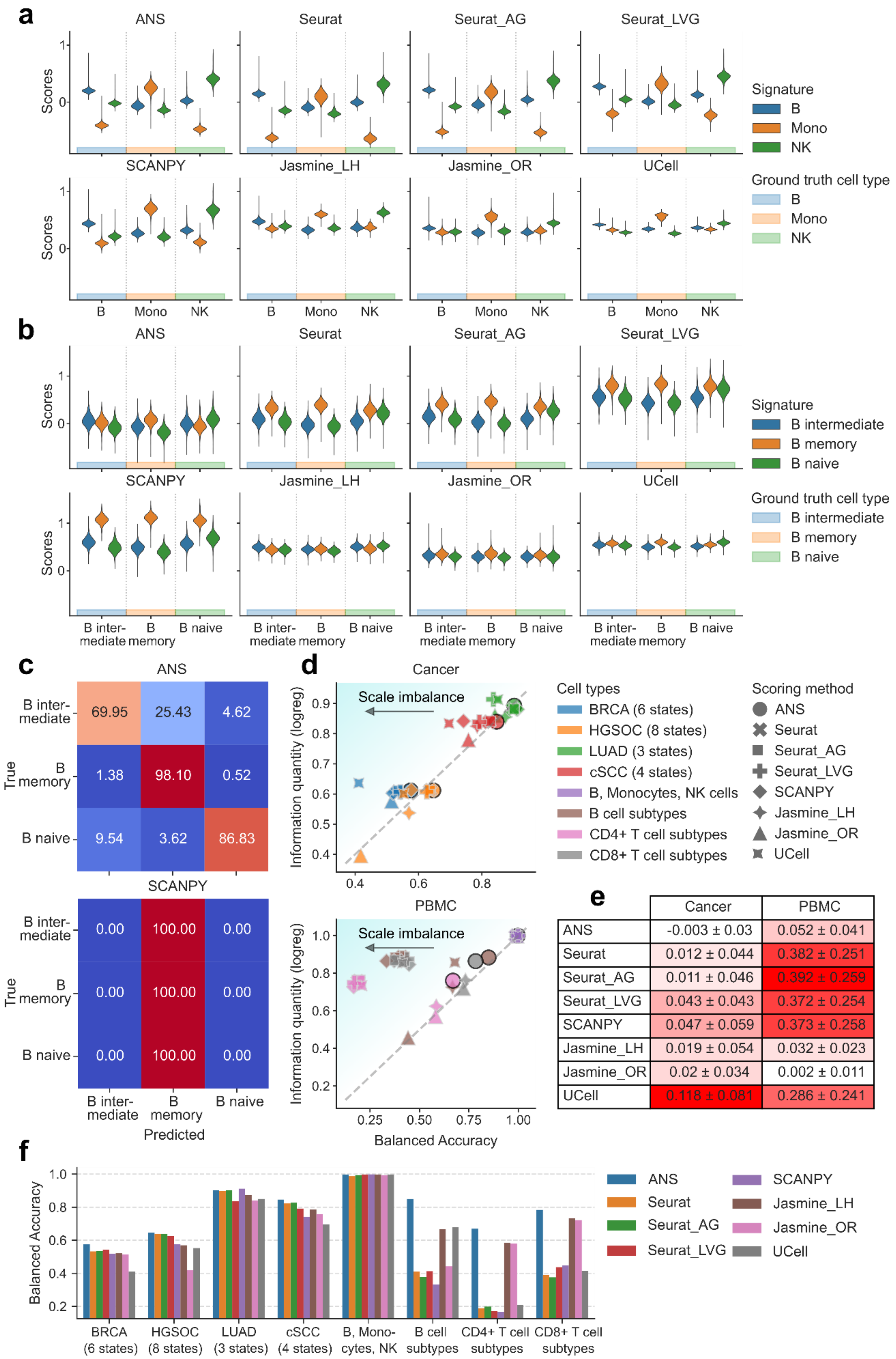

	Cancer	PBMC
ANS	-0.003 ± 0.03	0.052 ± 0.041
Seurat	0.012 ± 0.044	0.382 ± 0.251
Seurat_AG	0.011 ± 0.046	0.392 ± 0.259
Seurat_LVG	0.043 ± 0.043	0.372 ± 0.254
SCANPY	0.047 ± 0.059	0.373 ± 0.258
Jasmine_LH	0.019 ± 0.054	0.032 ± 0.023
Jasmine_OR	0.02 ± 0.034	0.002 ± 0.011
UCell	0.118 ± 0.081	0.286 ± 0.241

Supplemental Fig. S11: Comparative analysis of scoring methods for cell type and state annotation. Original cell type and state signatures were used potentially containing **overlapping gene sets**. **a,** Score distributions for cell-type-specific signatures (B cells, monocytes, and NK cells) separated by true cell type annotations, calculated for each scoring method. **b**, Score distributions for B cell subtype signatures separated by true cell subtypes, calculated by each scoring method. **c**, Row-normalized confusion matrix of B cell subtype annotation based on the highest scores. **d**, Relationship between hard labelling performance and score information quantity in cancer vs. PBMC datasets. Scatterplots show balanced accuracy (x-axis) against score information quantity (y-axis) for various scoring method-dataset combinations. Balanced accuracy quantifies hard labelling performance, while score information quantity indicates the scores' effectiveness in subtype classification. The diagonal line indicates perfect metric alignment, with vertical distances from this line representing scale imbalance. **e**, Quantitative analysis of scale imbalance across scoring methods and tissue types (cancer vs. PBMC). The mean and standard deviation of scale imbalance for each method are shown. Scale imbalance is the absolute difference between score information quantity and balanced accuracy in direct label assignment. The method with the lowest mean scale imbalance, indicating optimal consistency between information content and labelling accuracy, is highlighted in bold. **f**, Cell-state and -type annotation performance overview for all eight datasets and scoring methods.

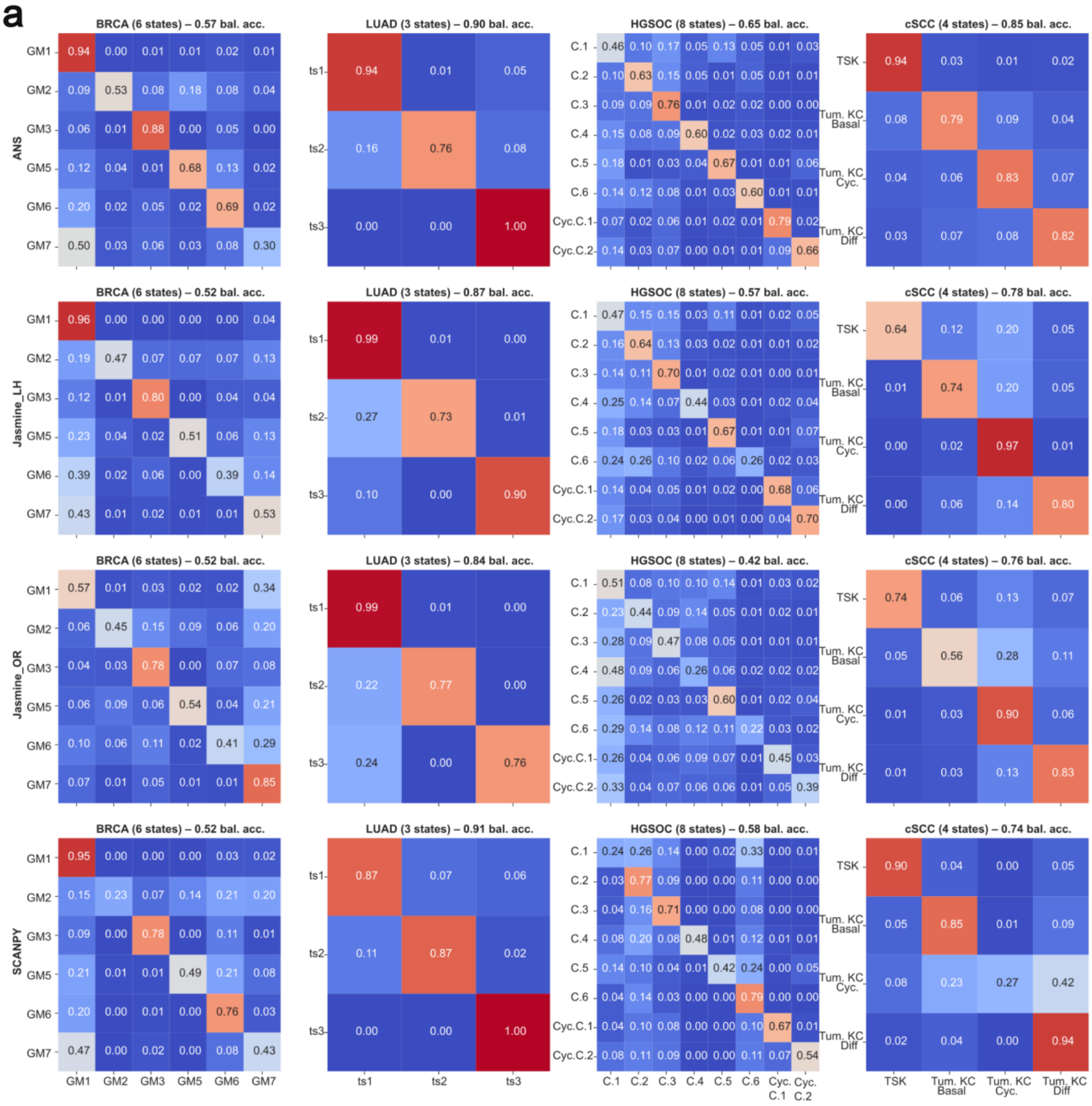
a
BRCA (6 states) – 0.57 bal. acc.
LUAD (3 states) – 0.90 bal. acc.
HGSOC (8 states) – 0.65 bal. acc.
cSCC (4 states) – 0.85 bal. acc.
BRCA (6 states) – 0.52 bal. acc.
LUAD (3 states) – 0.87 bal. acc.
HGSOC (8 states) – 0.57 bal. acc.
cSCC (4 states) – 0.78 bal. acc.
BRCA (6 states) – 0.52 bal. acc.
LUAD (3 states) – 0.84 bal. acc.
HGSOC (8 states) – 0.42 bal. acc.
cSCC (4 states) – 0.76 bal. acc.
BRCA (6 states) – 0.52 bal. acc.
LUAD (3 states) – 0.91 bal. acc.
HGSOC (8 states) – 0.58 bal. acc.
cSCC (4 states) – 0.74 bal. acc.
ANS
Jasmine_LH
Jasmine_OR
SCANPY
GM1
GM2
GM3
GM5
GM6
GM7
ts1
ts2
ts3
C.1
C.2
C.3
C.4
C.5
C.6
Cyc.C.1
Cyc.C.2
TSK
Tum. KC Basal
Tum. KC Cyc.
Tum. KC Diff

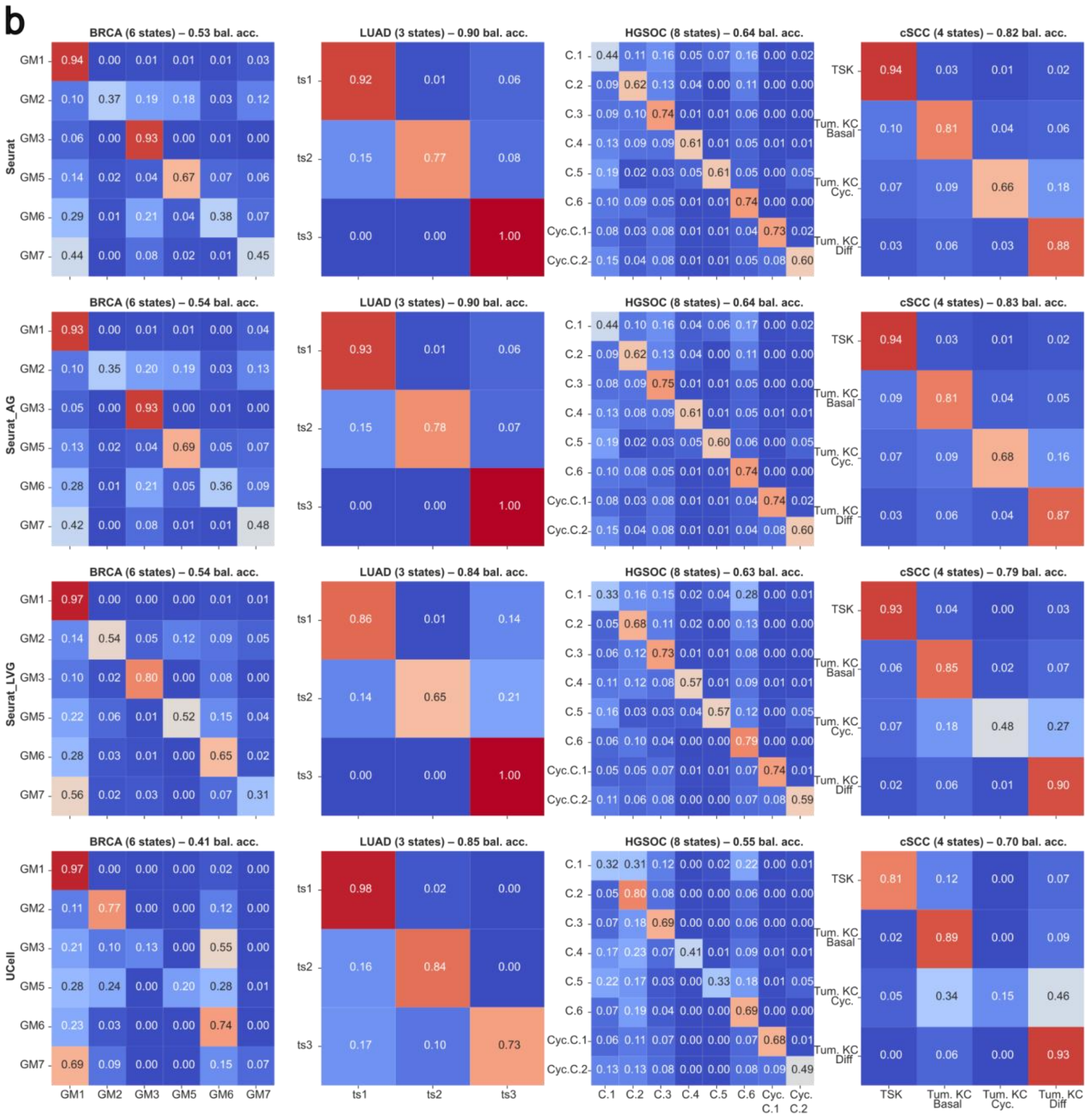
b
BRCA (6 states) – 0.53 bal. acc.
LUAD (3 states) – 0.90 bal. acc.
HGSOC (8 states) – 0.64 bal. acc.
cSCC (4 states) – 0.82 bal. acc.
Seurat
BRCA (6 states) – 0.54 bal. acc.
LUAD (3 states) – 0.90 bal. acc.
HGSOC (8 states) – 0.64 bal. acc.
cSCC (4 states) – 0.83 bal. acc.
Seurat_AG
BRCA (6 states) – 0.54 bal. acc.
LUAD (3 states) – 0.84 bal. acc.
HGSOC (8 states) – 0.63 bal. acc.
cSCC (4 states) – 0.79 bal. acc.
Seurat_LVG
BRCA (6 states) – 0.41 bal. acc.
LUAD (3 states) – 0.85 bal. acc.
HGSOC (8 states) – 0.55 bal. acc.
cSCC (4 states) – 0.70 bal. acc.
UCell
GM1 GM2 GM3 GM5 GM6 GM7
ts1 ts2 ts3
C.1 C.2 C.3 C.4 C.5 C.6 Cyc.C.1 Cyc.C.2
TSK Tum. KC Basal Tum. KC Cyc. Tum. KC Diff

c

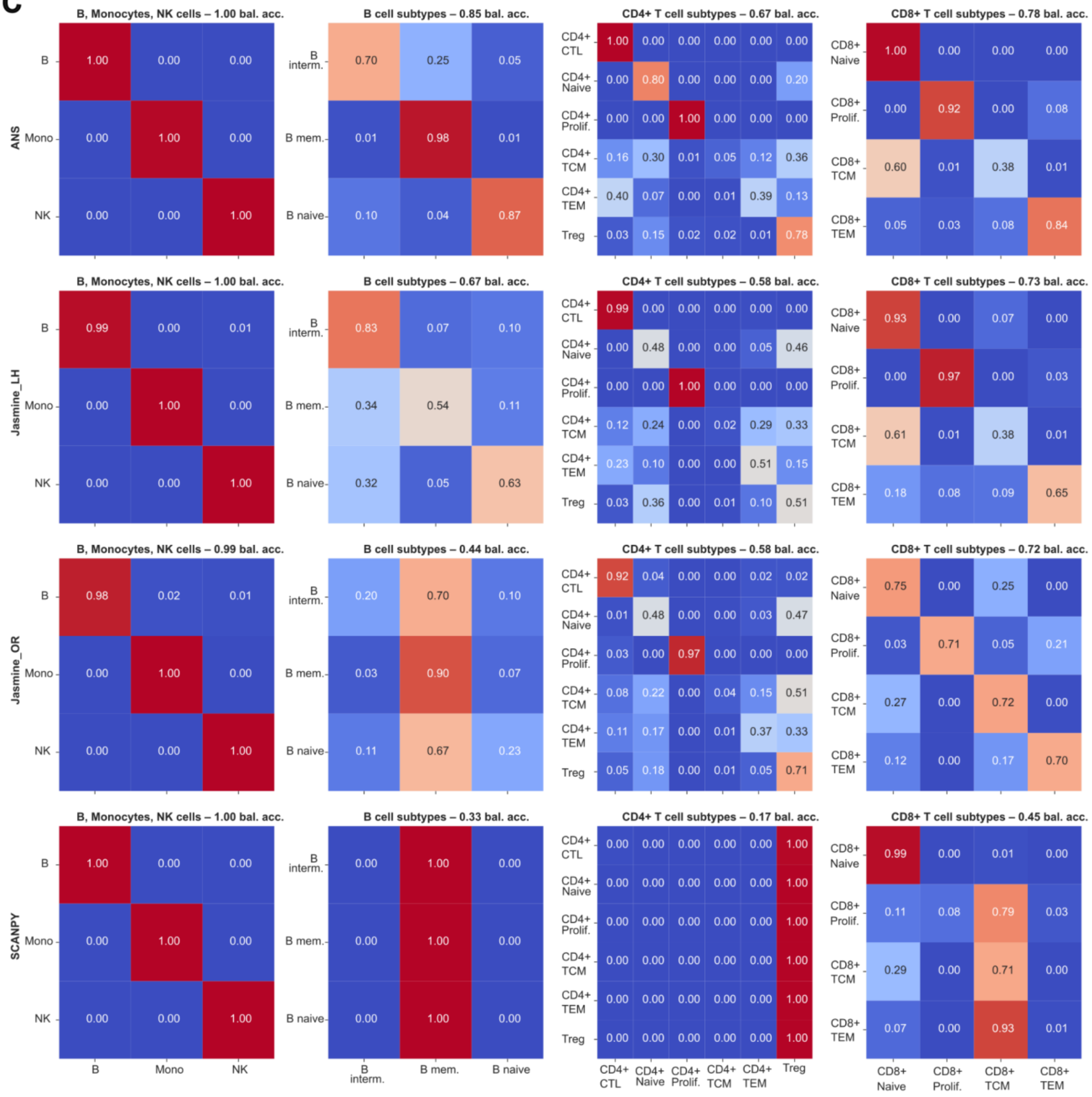
B, Monocytes, NK cells – 1.00 bal. acc.
B cell subtypes – 0.85 bal. acc.
CD4+ T cell subtypes – 0.67 bal. acc.
CD8+ T cell subtypes – 0.78 bal. acc.
ANS
B, Monocytes, NK cells – 1.00 bal. acc.
B cell subtypes – 0.67 bal. acc.
CD4+ T cell subtypes – 0.58 bal. acc.
CD8+ T cell subtypes – 0.73 bal. acc.
Jasmine_LH
B, Monocytes, NK cells – 0.99 bal. acc.
B cell subtypes – 0.44 bal. acc.
CD4+ T cell subtypes – 0.58 bal. acc.
CD8+ T cell subtypes – 0.72 bal. acc.
Jasmine_OR
B, Monocytes, NK cells – 1.00 bal. acc.
B cell subtypes – 0.33 bal. acc.
CD4+ T cell subtypes – 0.17 bal. acc.
CD8+ T cell subtypes – 0.45 bal. acc.
SCANPY
B
Mono
NK
B interm.
B mem.
B naive
CD4+ CTL
CD4+ Naive
CD4+ Prolif.
CD4+ TCM
CD4+ TEM
Treg
CD8+ Naive
CD8+ Prolif.
CD8+ TCM
CD8+ TEM

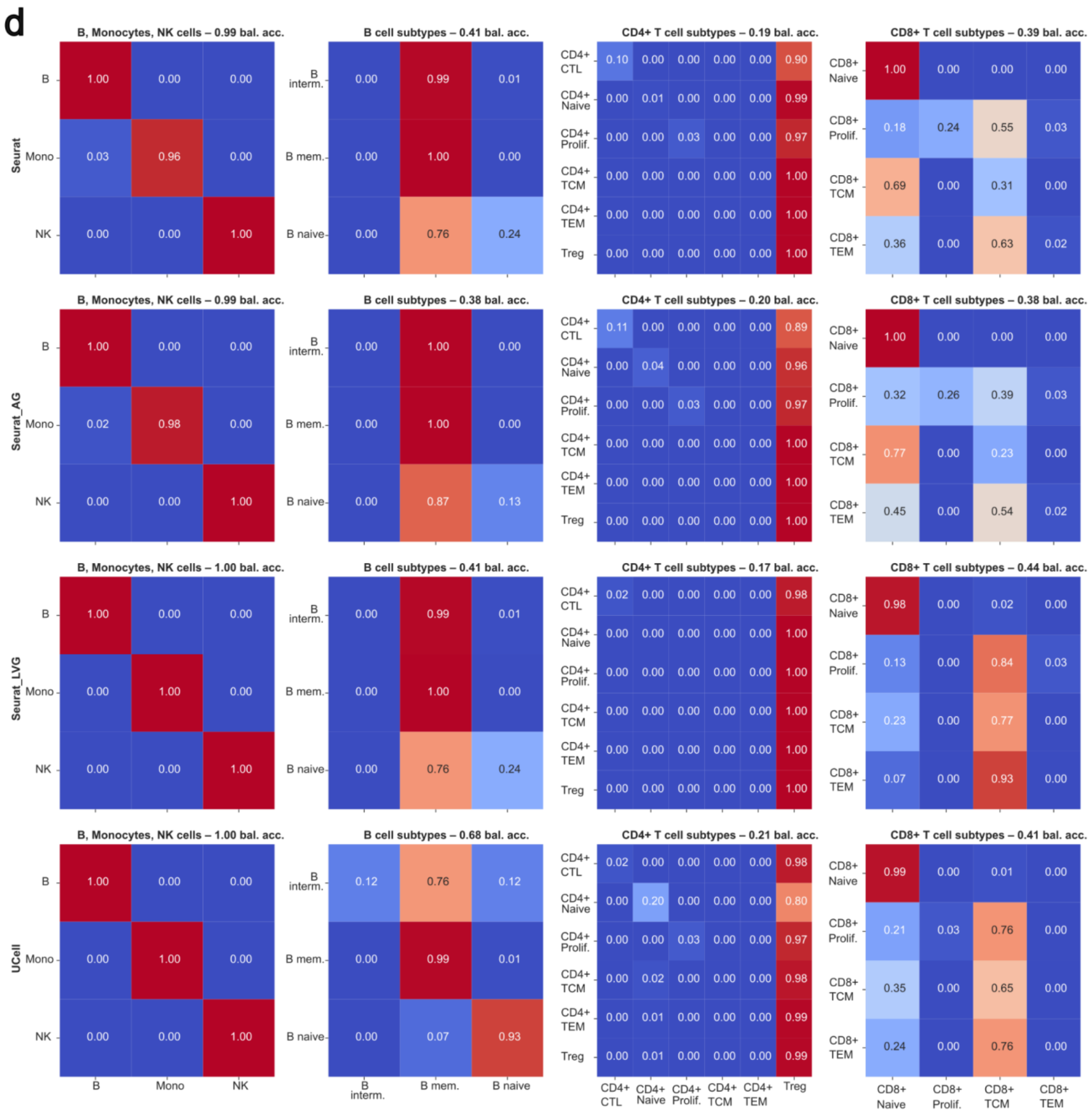

Supplemental Fig. S12: Confusion matrices for cell type/state prediction across multiple methods and datasets. **a**, Comparison of prediction performance between ANS, Jasmine_LH, Jasmine_OR, and SCANPY for cancer datasets (BRCA, LUAD, HGSOC, cSCC). **b**, Results for Seurat, Seurat_AG, Seurat_LVG, and UCell on the same cancer datasets. **c-d**, Performance evaluation across immune cell subsets using the same methods, showing B/Monocytes/NK cells, B cell subtypes, CD4+ T cell subtypes, and CD8+ T cell subtypes. Each matrix shows the relationship between true (y-axis) and predicted (x-axis) cell states, with values summing to one per row. The color scale ranges from blue (0) to red (1), with balanced accuracy (bal. acc.) shown for each matrix. Overlapping/ Original gene signatures were used to score cell states/types in each dataset.

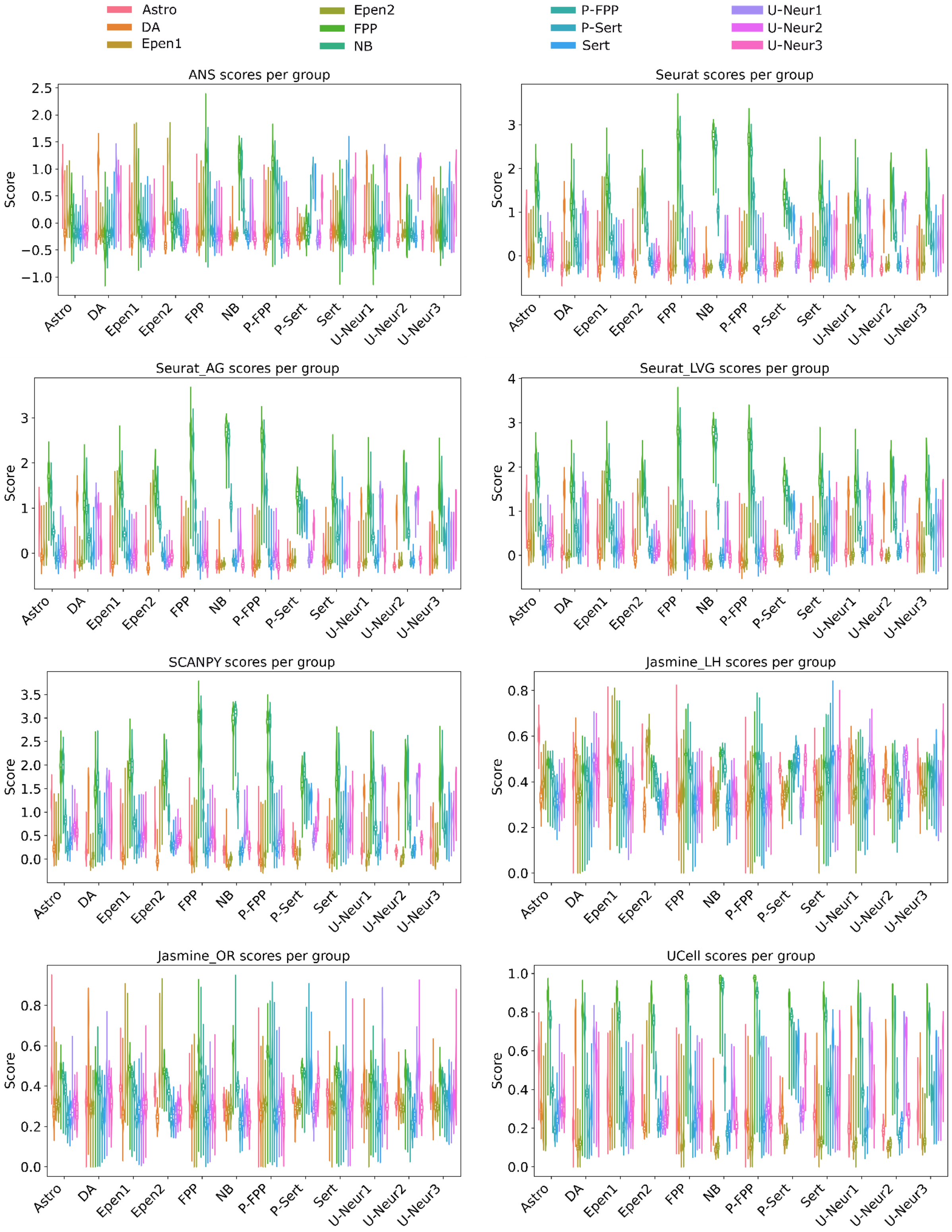

Supplemental Fig. S13: Violin plots showing the distribution of scores for each scored signature per cell type (group) across benchmarked scoring methods. For each cell group, we expect the highest scores for the signature associated with ground truth cell labels (x-axis).

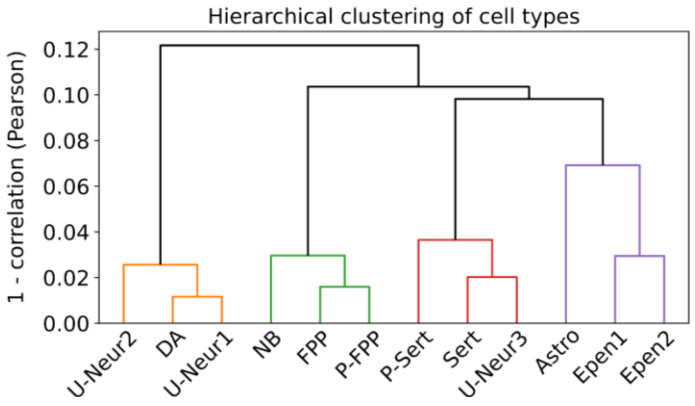

Supplemental Fig. S14: Hierarchical clustering based on Pearson correlation similarity of the 12 cell types present in the dataset. For each dataset, average gene expression was used for comparison. Colored branches correspond to clusters indicating more similar cell types. Floor plate progenitors (FPP); Proliferating floor plate progenitors (P_FPP); Neuroblasts (NB); Dopaminergic neurons (DA); Serotonergic-like neurons (Serts); Proliferating serotonergic-like neurons (P_Serts); Astrocyte-like (Astro); Ependymal-like 1 (Epen1); Ependymal-like 2 (Epen2); Unknown neuron groups 1-3 (U_Neur1, U_Neur2, U_Neur3).

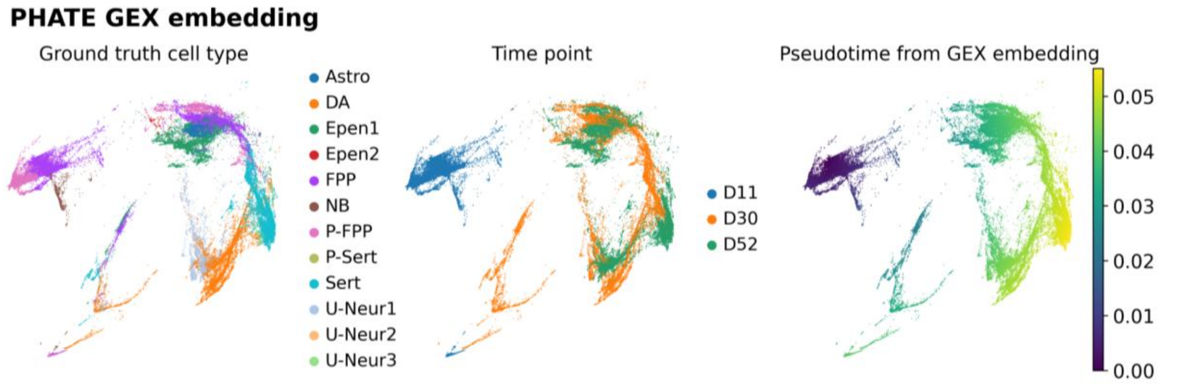

Supplemental Fig. S15: PHATE embeddings of the neuronal differentiation dataset based on the full gene expression matrix (GEX). The plots are colored by ground-truth cell type annotations, sequencing time points (days), and pseudotime estimated from the embedding.

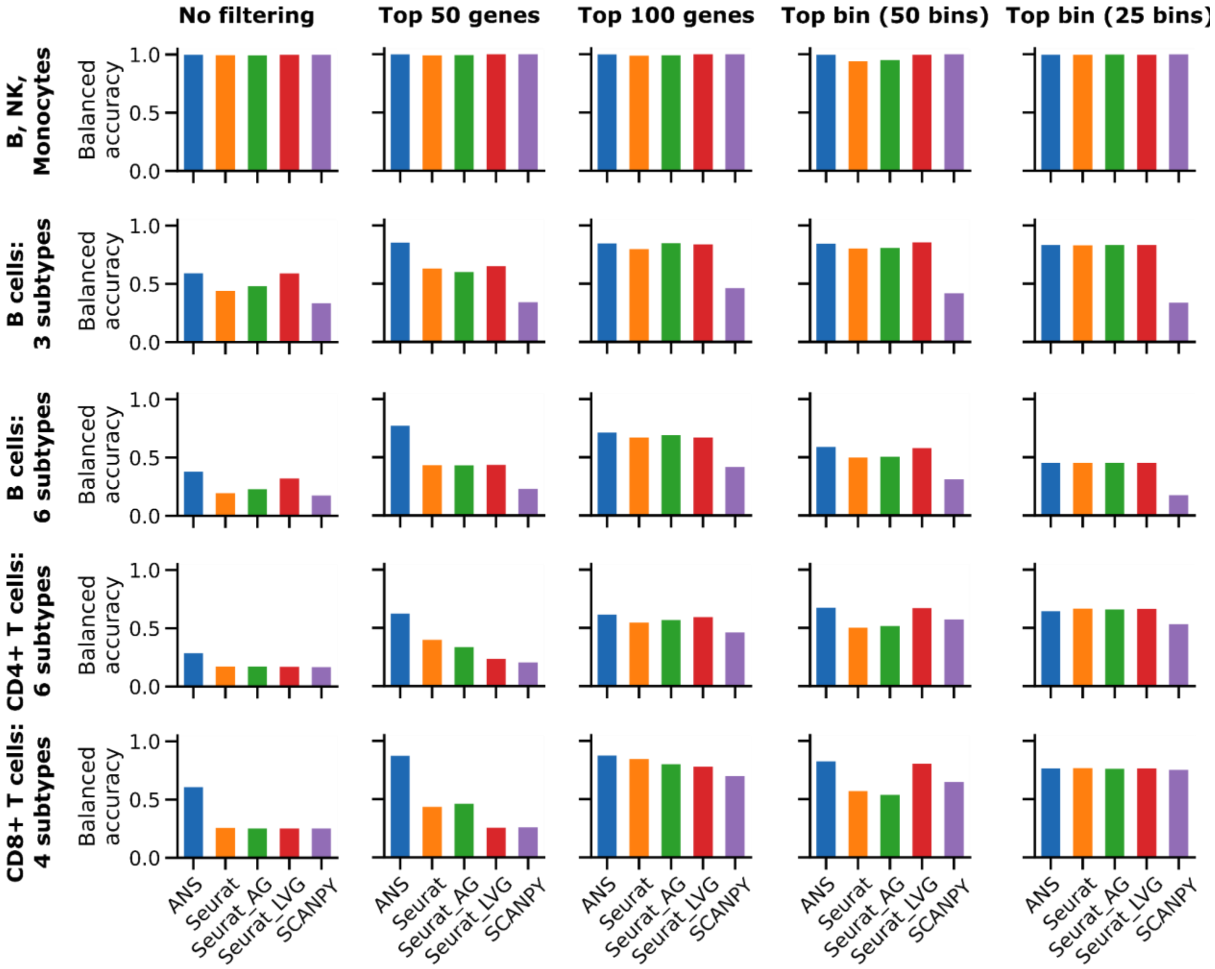

Supplemental Fig. S16: Performance of ANS and Tirosh-based scoring approaches for cell type annotation under varied signature gene-filtering strategies. Overall balanced accuracy per dataset and filtering strategy is reported.

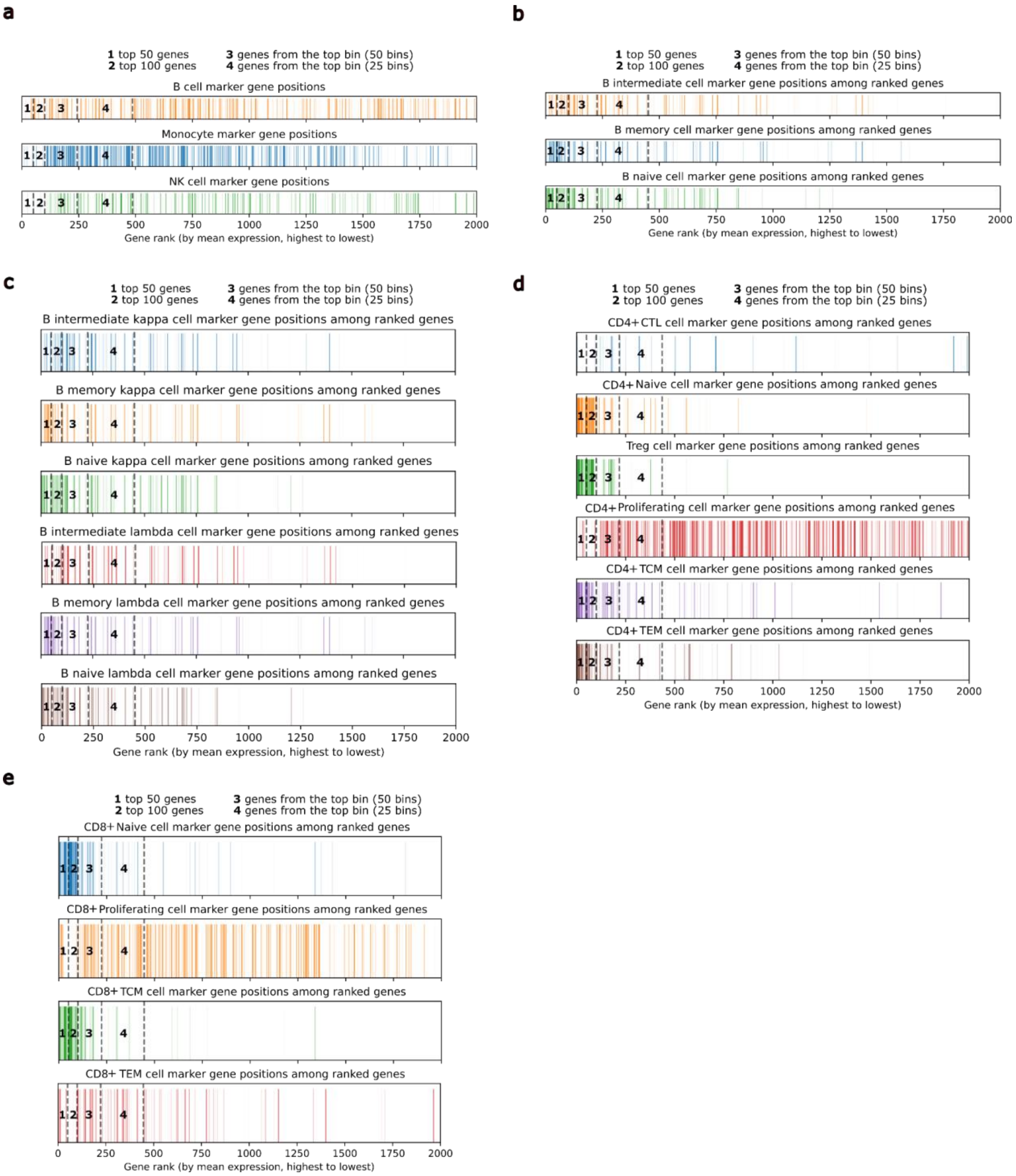

Supplemental Fig. S17: The position of signature genes within a ranked list of expressed genes across analysed datasets . Genes are ranked based on average expression across all cells within a dataset (from highest on the left to lowest on the right). **a.** PBMC dataset with NK cells, B cells and Monocyte. **b**. PBMC dataset with three subtypes of B cells. **c**. PBMC dataset with six subtypes of B cells. **d**. PBMC dataset with six subtypes of CD4+ T cells. **e**. PBMC dataset with four subtypes of CD8+ T cells.

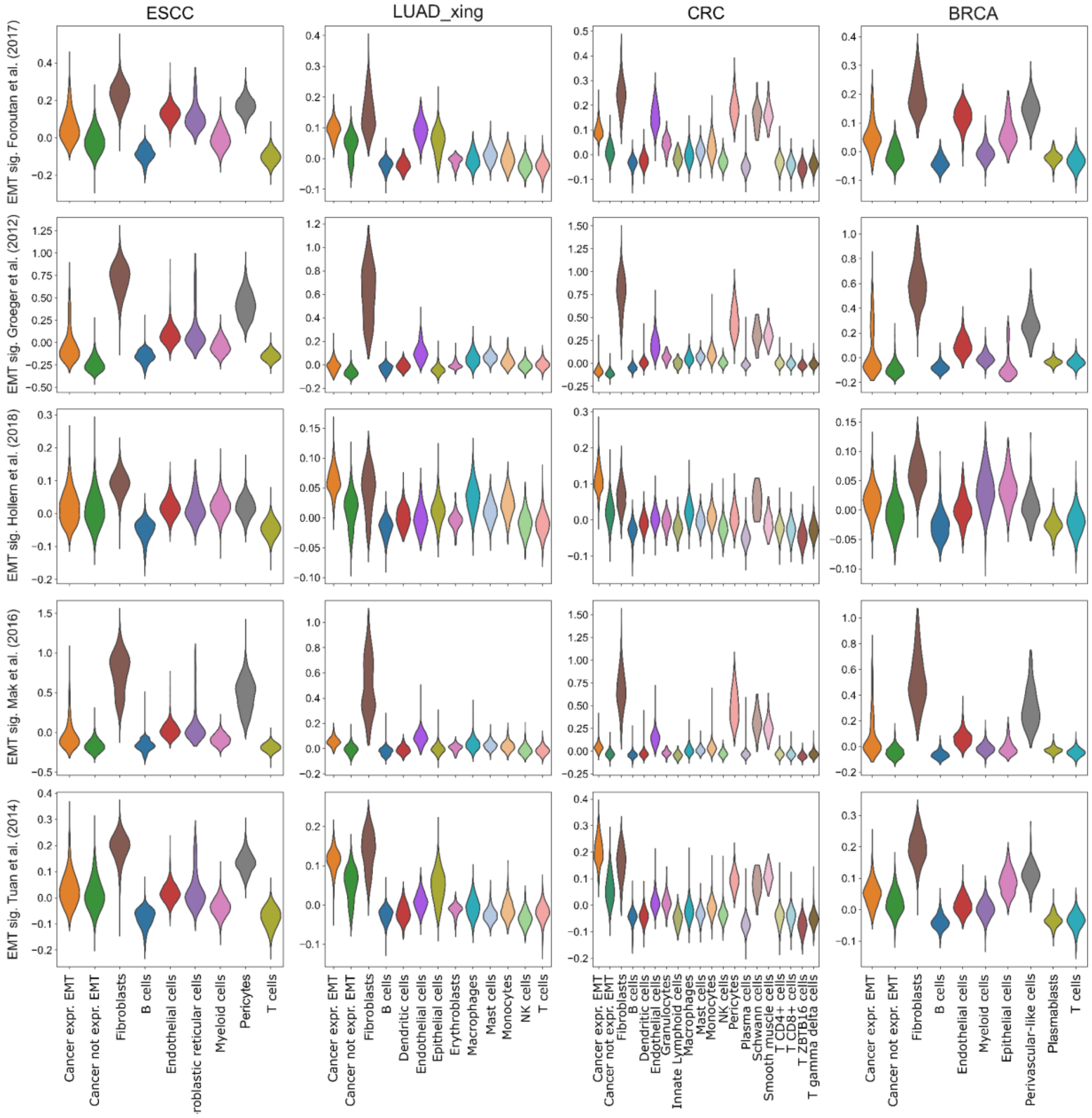

Supplemental Fig. S18: Score distributions by cell types for the remaining five pan-cancer EMT signatures on ESCC, LUAD_xing, CRC, and BRCA.

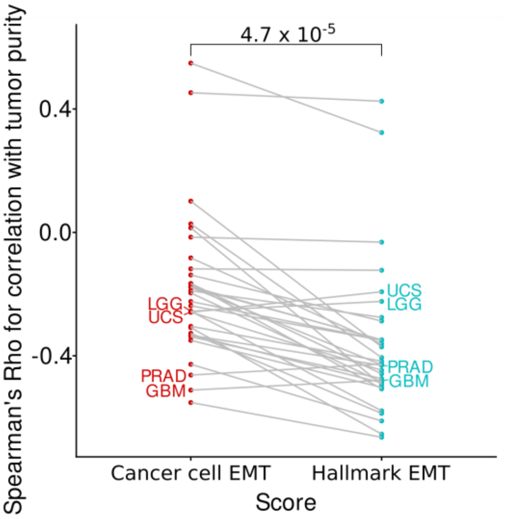

Supplemental Fig. S19: Separman's correlation coefficients between tumor purity estimates and the two EMT signatures across 33 TCGA datasets: ANS-derived Cancer cell EMT and the classic Hallmark EMT signature (Liberzon et al. 2015). Values for the same cancer types are connected with gray lines. The four cancer types for which correlation with purity is higher for Hallmark EMT scores are reported. Statistical significance of the comparison between Cancer cell EMT and Hallmark EMT correlation values was calculated using the Wilcoxon signed-rank test.

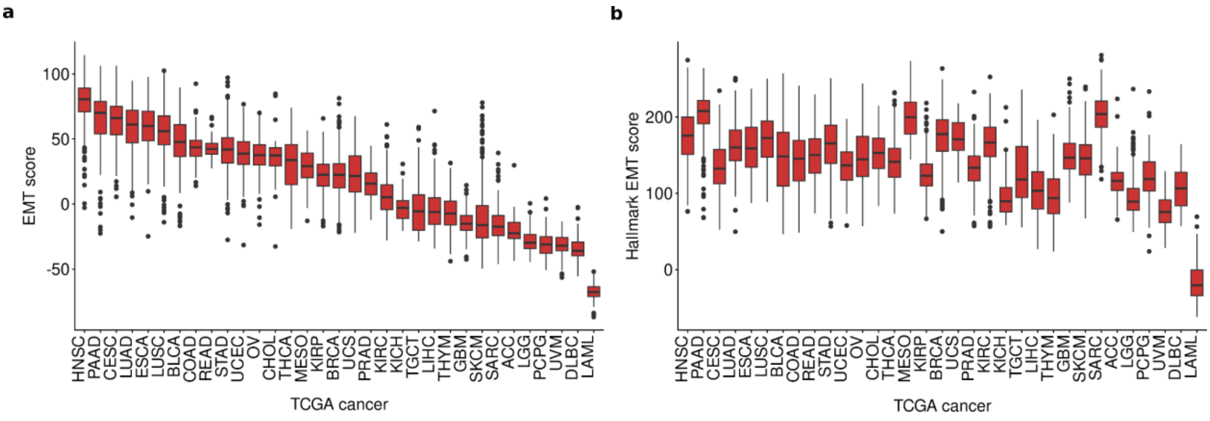

Supplemental Fig. S20: Distribution of the ANS-derived cancer cell-specific EMT signature (**a**) and hallmark EMT signature (**b**) across 33 TCGA cancer types.

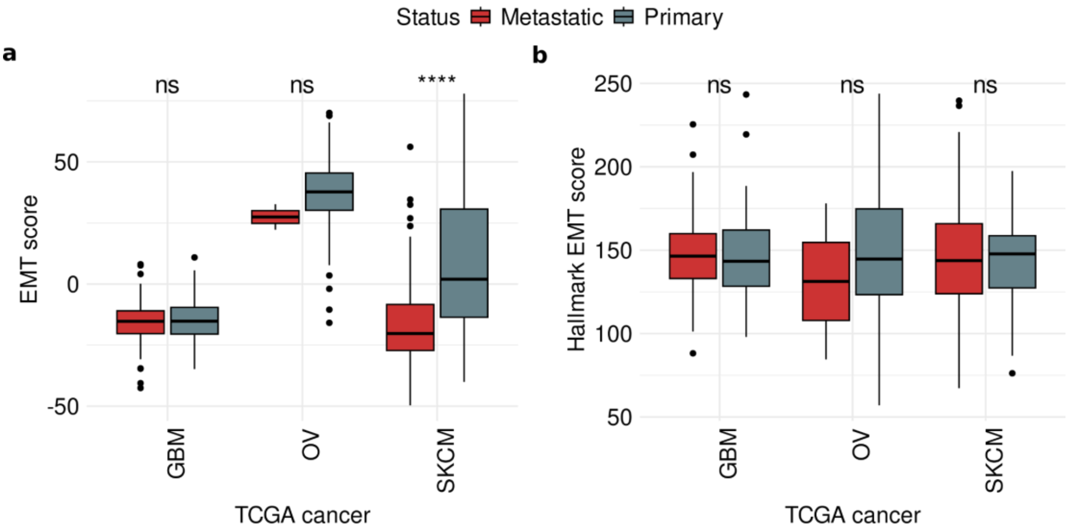

Supplemental Fig. S21: Distribution of the ANS-derived cancer cell-specific EMT signature across TCGA datasets with primary and metastatic samples: GBM (primary = 90, metastatic = 65), OV (primary = 294, metastatic = 2), and SKCM (primary = 102, metastatic = 340). Statistical significance of the comparison between metastatic and primary sites was calculated using Wilcoxon's test. ns: p-value > 0.05, ****: p-value < 0.0001.

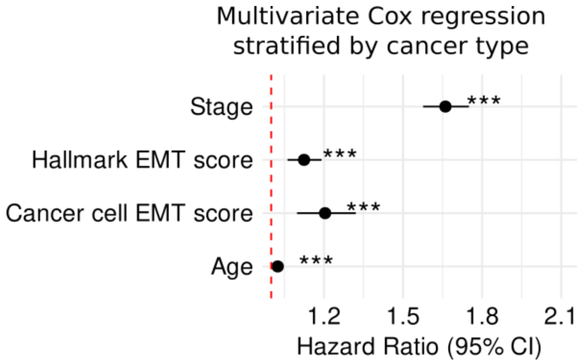

Supplemental Fig. S22: Multivariate Cox regression analysis of TCGA datasets, with tumor stage, hallmark EMT score, cancer cell-specific EMT score and patient age. The model was stratified by cancer type. The red line corresponds to the hazard ratio of 1. Log-rank test p-value for each variable is reported as stars. ***: p-value < 0.001.

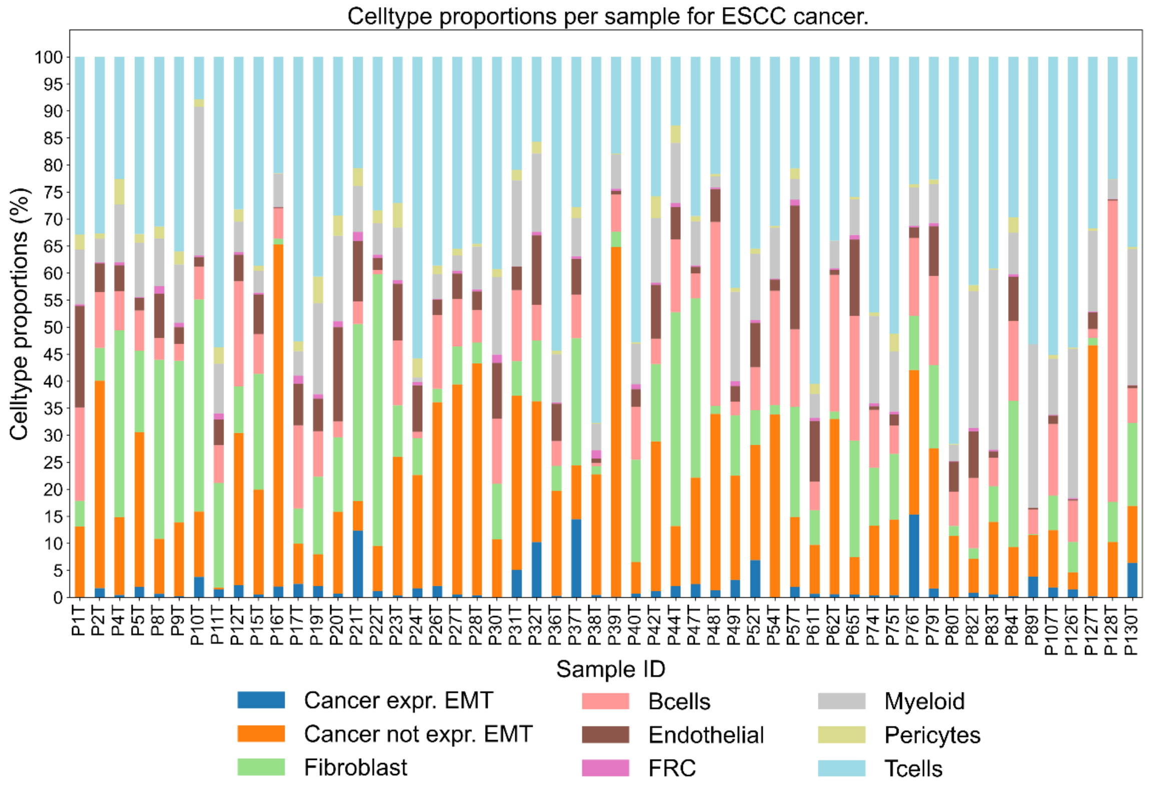
Celltype proportions per sample for ESCC cancer.
Celltype proportions (%)
Sample ID
Cancer expr. EMT
Cancer not expr. EMT
Fibroblast
Bcells
Endothelial
FRC
Myeloid
Pericytes
Tcells

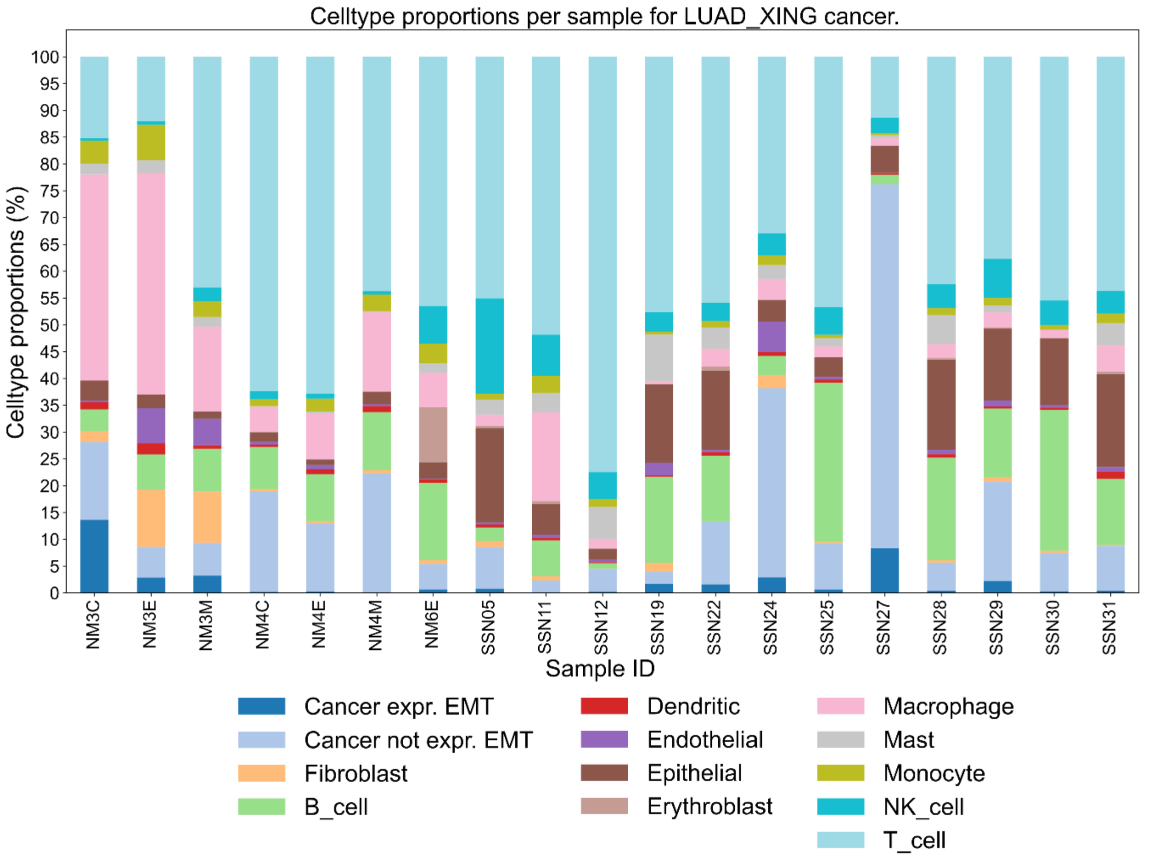
Celltype proportions per sample for LUAD_XING cancer.
Celltype proportions (%)
Sample ID
Cancer expr. EMT
Cancer not expr. EMT
Fibroblast
B_cell
Dendritic
Endothelial
Epithelial
Erythroblast
Macrophage
Mast
Monocyte
NK_cell
T_cell

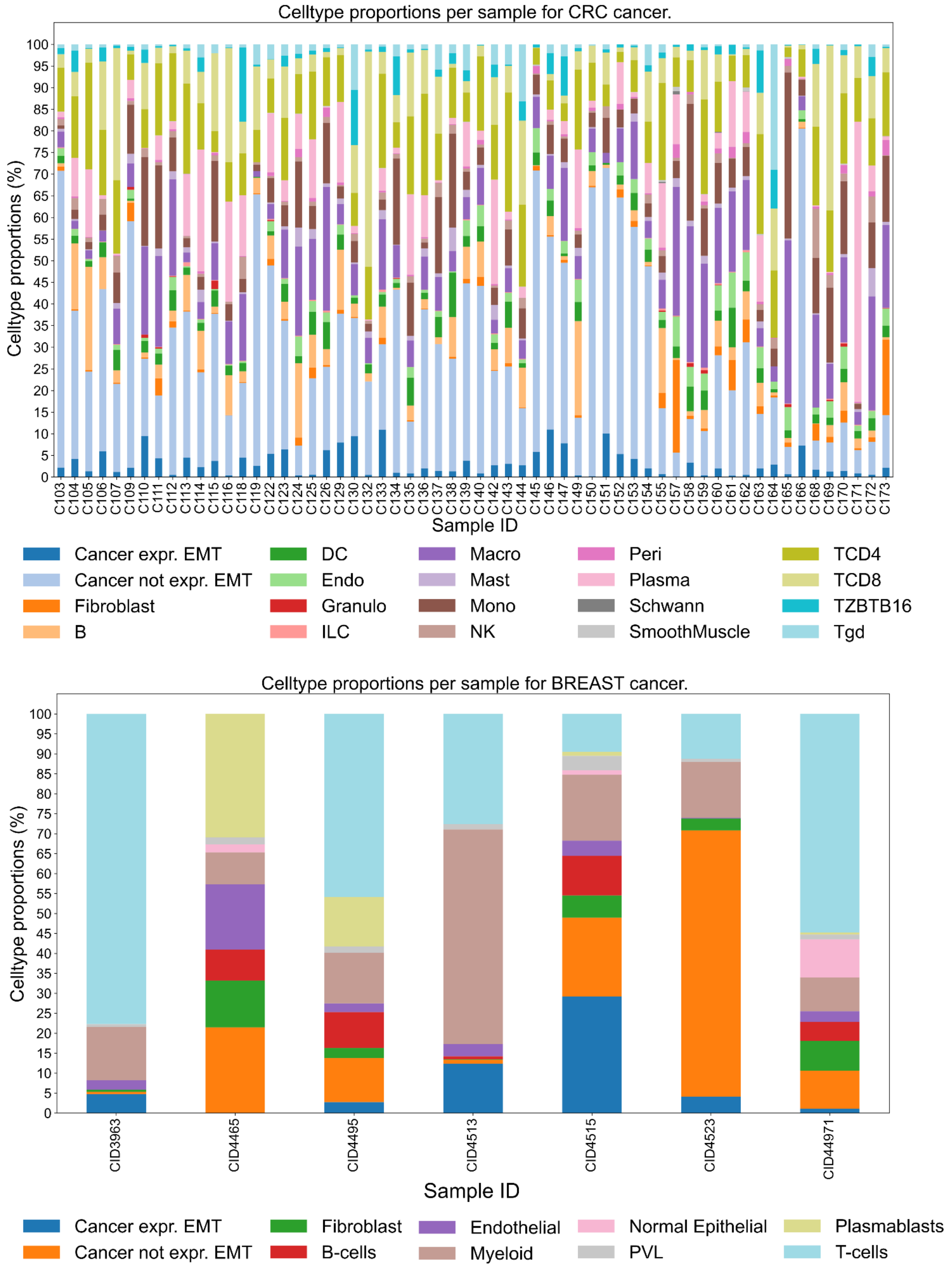

Supplemental Fig. S23: Dataset compositions for ESCC, LUAD_Xing, CRC, and breast carcinoma (BREAST), including cancer EMT cell annotations.

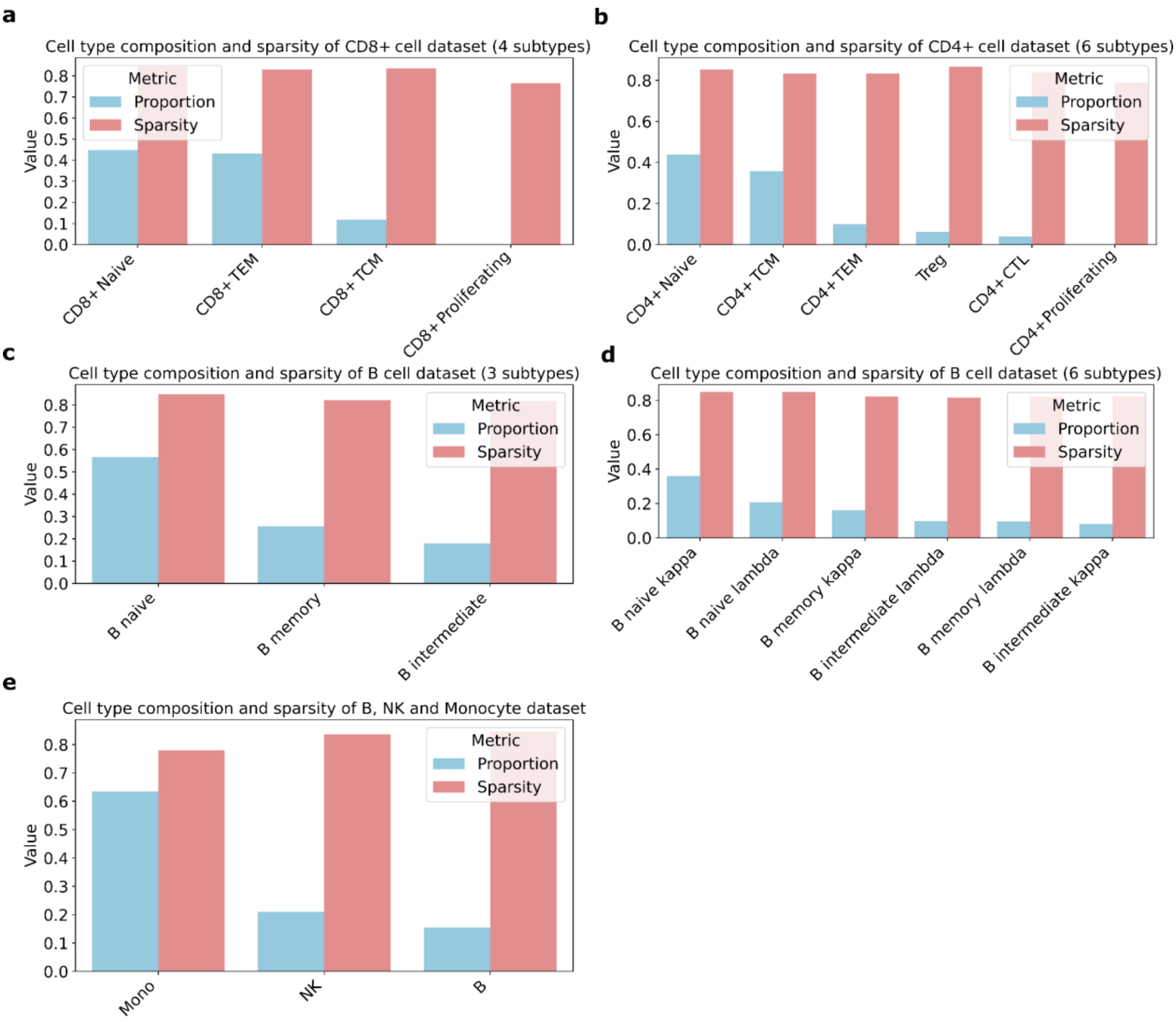

Supplemental Fig. S24: Distribution of cell type and state proportions and sparsity across analyzed PBMC datasets: **a.** Four subtypes of CD8+ T cells, **b.** Six subtypes of CD4+ T cells, **c.** Three subtypes of B cells, **d.** Six subtypes of B cells; and **e.** Dataset composed of B cells, NK cells and monocytes.

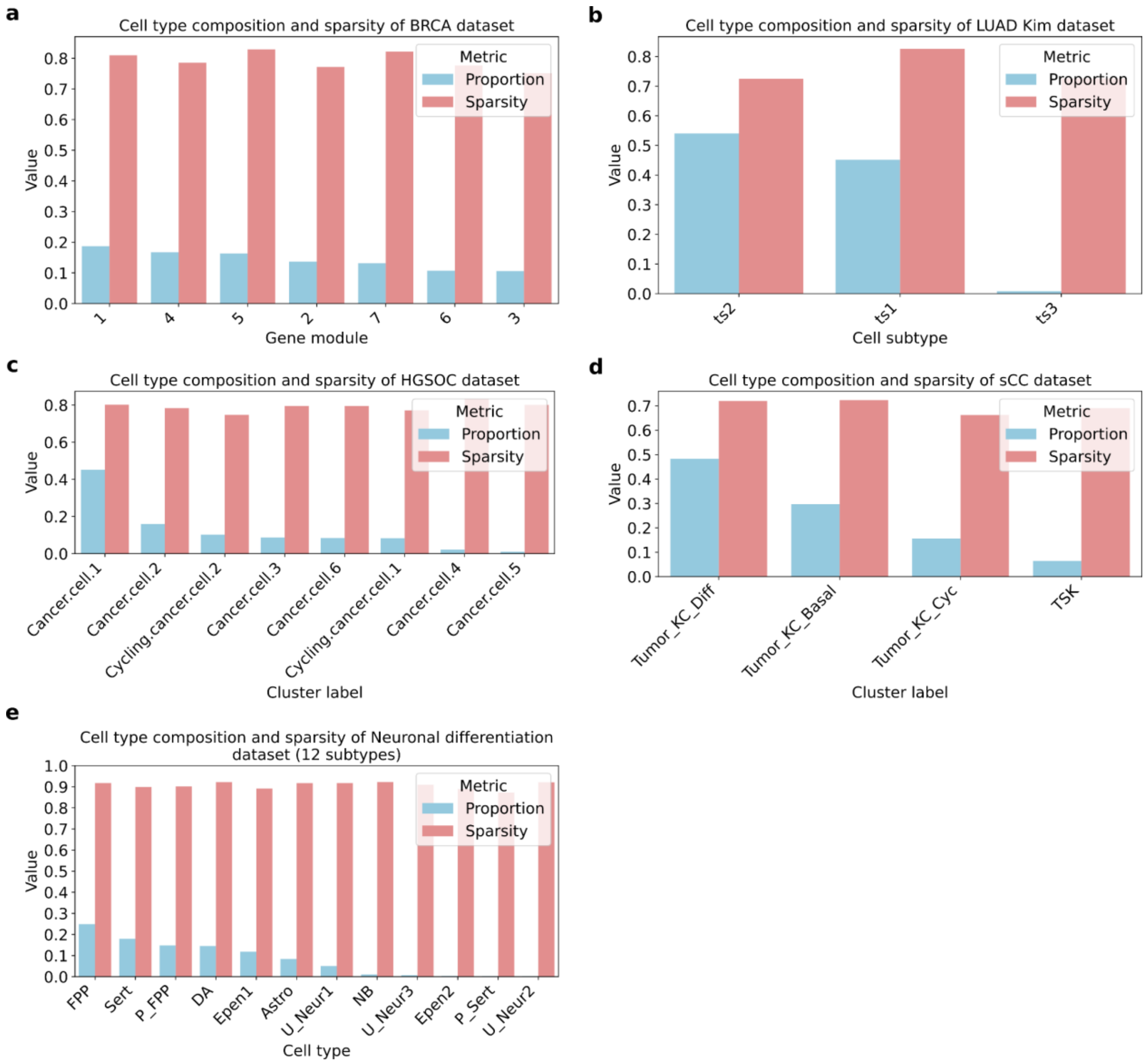

Supplemental Fig. S25: Distribution of cell type and state proportions and sparsity across analyzed cancer datasets: **a.** Seven malignant cell states in BRCA dataset, **b.** Three malignant cell states in LUAD Kim dataset, **c.** Eight malignant cell states in HGSOC dataset, **d.** Four malignant cell states in sCC dataset; and **e.** Neuronal differentiation datasets composed of twelve cell subtypes.

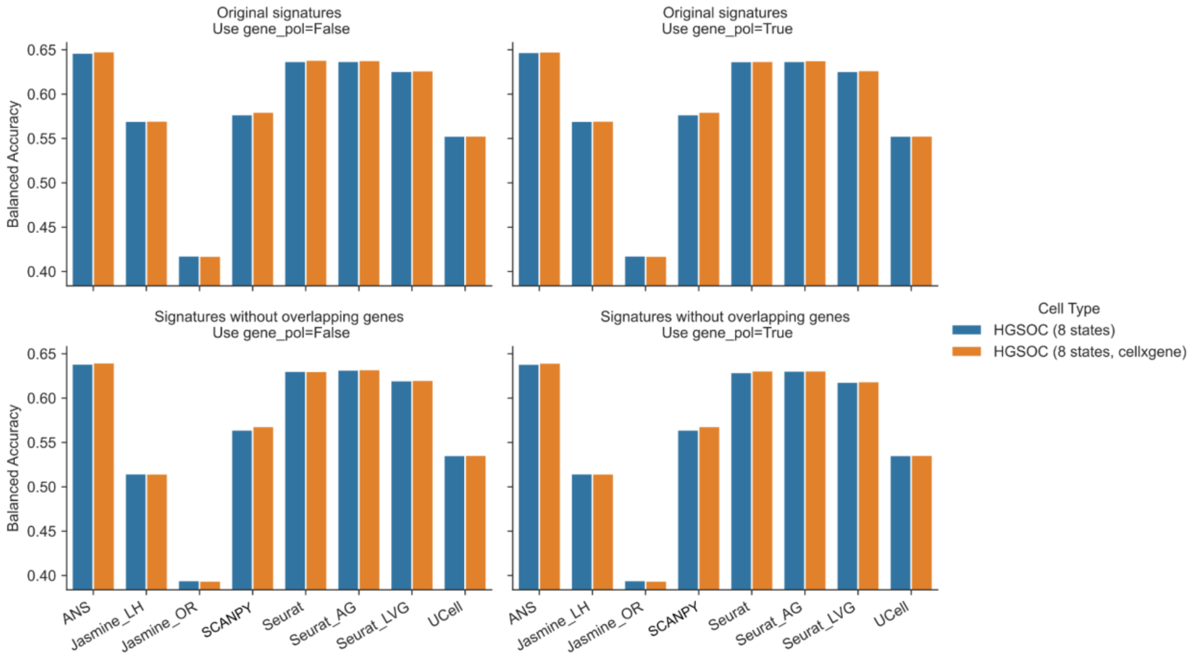

Supplemental Fig. S26: Comparison of cell-state annotation performance in ovarian cancer using different data preprocessing approaches. The balanced accuracy of the considered scoring methods is shown for two data processing pipelines: (1) GEO data (GSE180661) with 1% gene filtering and mean-shift log-normalization on cancer cells (HGSOC 8 states), and (2) Raw data filtered using curated cells from cellxgene, followed by 1% gene filtering and mean-shift log-normalization (HGSOC 8 states, cellxgene)[1]. Results are presented for both original signatures and signatures without overlapping genes, with two different control gene selection strategies: excluding signature genes from the overall control gene pool (gene_pol=True) or allowing them to be included (gene_pol=False). The highly similar performance between both approaches suggests that the GEO dataset primarily contains the curated cells, and additional filtering steps do not significantly impact the annotation results. ANS consistently shows the highest balanced accuracy (~0.65) across all conditions, while Jasmine_OR performs the poorest (~0.40).

[1]CellxGene preprocessed dataset: https://cellxgene.cziscience.com/collections/4796c91c-9d8f-4692-be43-347b1727f9d8

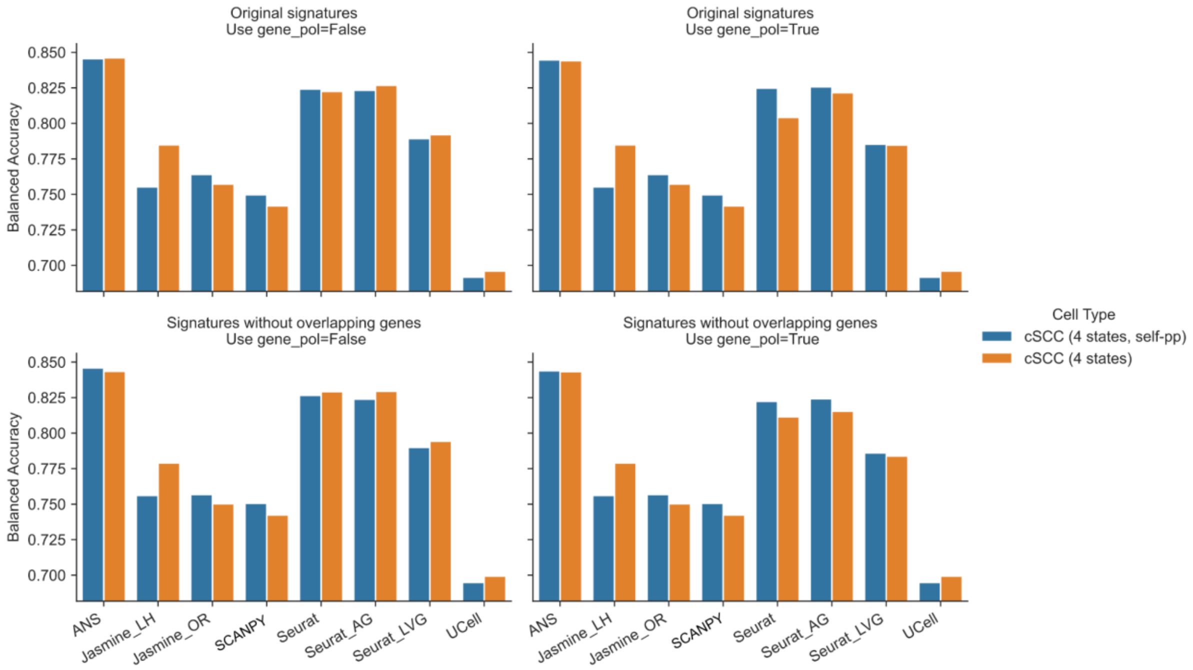

Supplemental Fig. S27: Comparison of cell-state annotation performance in cutaneous squamous cell carcinoma (cSCC) using different preprocessing approaches. The balanced accuracy of the considered scoring methods is shown for two data processing pipelines: (1) GEO data (GSE144240, GSE144236) with 1% gene filtering and mean-shift log-normalization on cancer cells (cSCC 4 states, self-pp), and (2) Data processed through the CanSig pipeline followed by 1% gene filtering and mean-shift normalization (cSCC 4 states). Results are presented for both original signatures and signatures without overlapping genes, with two different control gene selection strategies: excluding signature genes from the overall control gene pool (gene_pol=True) or allowing them to be included (gene_pol=False). The similar performance patterns between both approaches indicate that the preprocessing steps in the CanSig pipeline do not significantly alter the annotation results. ANS demonstrates the highest balanced accuracy (~0.85) across all conditions, while UCell consistently shows the lowest performance (~0.70).

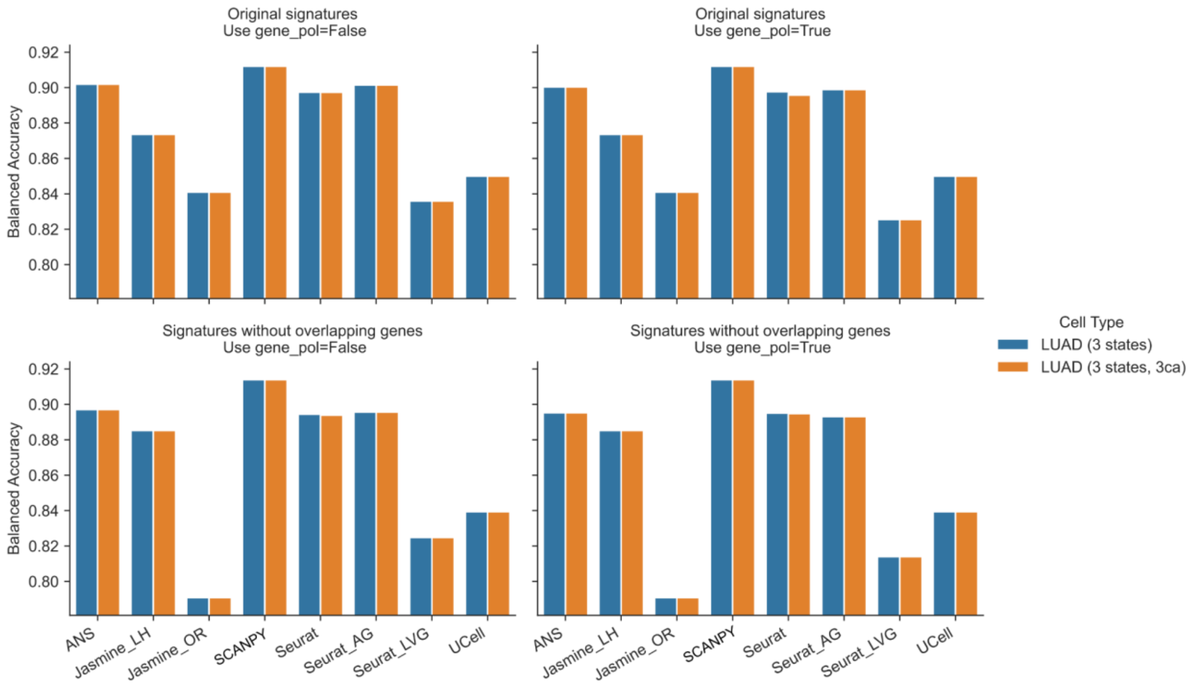

Supplemental Fig. S28: Impact of data preprocessing methods and gene selection for cell states annotation. We compared two preprocessing approaches: the CanSig pipeline and the 3CA pipeline[2], which implements different cell and gene filtering criteria. The ovarian cancer dataset was processed using 3CA only. For the lung adenocarcinoma (LUAD) dataset from Kim *et al.*, we performed parallel analyses using both CanSig and 3CA preprocessing methods. In both cases, we applied additional filtering to remove genes expressed in less than 1% of cells and performed log-normalization before calculating signature scores. For methods requiring a gene pool for control gene selection, we evaluated two strategies: (1) excluding all signature genes from the pool and (2) placing no restrictions on pool composition. The figure displays a matrix where rows show the balanced accuracy for signatures with overlapping versus non-overlapping genes, and columns indicate whether a gene pool was utilized.

References

Ahlmann-Eltze C, Huber W. 2023. Comparison of transformations for single-cell RNA-seq data. *Nat Methods* **20**: 665–672.

Barkley D, Moncada R, Pour M, Liberman DA, Dryg I, Werba G, Wang W, Baron M, Rao A, Xia B, et al. 2022. Cancer cell states recur across tumor types and form specific interactions with the tumor microenvironment. *Nat Genet* **54**: 1192–1201.

Barkmann F, Yates J, Czyż P, Kraft A, Glettig M, Beerenwinkel N, Boeva V. 2025. CanSig Benchmarks Methods for Reproducible Cancer Cell State Discovery from Single-Cell Transcriptomic Data. *Cancer Res*. http://dx.doi.org/10.1158/0008-5472.CAN-25-0940.

Foroutan M, Cursons J, Hediyeh-Zadeh S, Thompson EW, Davis MJ. 2017. A Transcriptional Program for Detecting TGFβ-Induced EMT in Cancer. *Mol Cancer Res* **15**: 619–631.

---

[2]3CA: Curated Cancer Cell Atlas (https://www.weizmann.ac.il/sites/3CA/) contains multiple curated cancer scRNA-seq datasets. 3CA uses its own pipeline to curate datasets.

Gröger CJ, Grubinger M, Waldhör T, Vierlinger K, Mikulits W. 2012. Meta-analysis of gene expression signatures defining the epithelial to mesenchymal transition during cancer progression. *PLoS One* **7**: e51136.

Hao Y, Hao S, Andersen-Nissen E, Mauck WM 3rd, Zheng S, Butler A, Lee MJ, Wilk AJ, Darby C, Zager M, et al. 2021. Integrated analysis of multimodal single-cell data. *Cell* **184**: 3573–3587.e29.

Heumos L, Schaar AC, Lance C, Litinetskaya A, Drost F, Zappia L, Lücken MD, Strobl DC, Henao J, Curion F, et al. 2023. Best practices for single-cell analysis across modalities. *Nat Rev Genet* **24**: 550–572.

Hollern DP, Swiatnicki MR, Andrechek ER. 2018. Histological subtypes of mouse mammary tumors reveal conserved relationships to human cancers. *PLoS Genet* **14**: e1007135.

Jerber J, Seaton DD, Cuomo ASE, Kumasaka N, Haldane J, Steer J, Patel M, Pearce D, Andersson M, Bonder MJ, et al. 2021. Population-scale single-cell RNA-seq profiling across dopaminergic neuron differentiation. *Nat Genet* **53**: 304–312.

Ji AL, Rubin AJ, Thrane K, Jiang S, Reynolds DL, Meyers RM, Guo MG, George BM, Mollbrink A, Bergenstråhle J, et al. 2020. Multimodal analysis of composition and spatial architecture in human squamous cell carcinoma. *Cell* **182**: 497–514.e22.

Kim N, Kim HK, Lee K, Hong Y, Cho JH, Choi JW, Lee J-I, Suh Y-L, Ku BM, Eum HH, et al. 2020. Single-cell RNA sequencing demonstrates the molecular and cellular reprogramming of metastatic lung adenocarcinoma. *Nat Commun* **11**: 2285.

Liberzon A, Birger C, Thorvaldsdóttir H, Ghandi M, Mesirov JP, Tamayo P. 2015. The Molecular Signatures Database (MSigDB) hallmark gene set collection. *Cell Syst* **1**: 417–425.

Mak MP, Tong P, Diao L, Cardnell RJ, Gibbons DL, William WN, Skoulidis F, Parra ER, Rodriguez-Canales J, Wistuba II, et al. 2016. A Patient-Derived, Pan-Cancer EMT Signature Identifies Global Molecular Alterations and Immune Target Enrichment Following Epithelial-to-Mesenchymal Transition. *Clin Cancer Res* **22**: 609–620.

Pelka K, Hofree M, Chen JH, Sarkizova S, Pirl JD, Jorgji V, Bejnood A, Dionne D, Ge WH, Xu KH, et al. 2021. Spatially organized multicellular immune hubs in human colorectal cancer. *Cell* **184**: 4734–4752.e20.

Soneson C, Robinson MD. 2018. Bias, robustness and scalability in single-cell differential expression analysis. *Nat Methods* **15**: 255–261.

Tan TZ, Miow QH, Miki Y, Noda T, Mori S, Huang RY-J, Thiery JP. 2014. Epithelial-mesenchymal transition spectrum quantification and its efficacy in deciphering survival and drug responses of cancer patients. *EMBO Mol Med* **6**: 1279–1293.

Tirosh I, Izar B, Prakadan SM, Wadsworth MH 2nd, Treacy D, Trombetta JJ, Rotem A, Rodman C, Lian C, Murphy G, et al. 2016. Dissecting the multicellular ecosystem of metastatic melanoma by single-cell RNA-seq. *Science* **352**: 189–196.

Vasaikar SV, Deshmukh AP, den Hollander P, Addanki S, Kuburich NA, Kudaravalli S, Joseph R, Chang JT, Soundararajan R, Mani SA. 2021. EMTome: a resource for pan-cancer analysis of epithelial-mesenchymal transition genes and signatures. *Br J Cancer* **124**: 259–269.

Vázquez-García I, Uhlitz F, Ceglia N, Lim JLP, Wu M, Mohibullah N, Niyazov J, Ruiz AEB, Boehm KM, Bojilova V, et al. 2022. Ovarian cancer mutational processes drive site-specific immune evasion. *Nature* **612**: 778–786.

Wu SZ, Al-Eryani G, Roden DL, Junankar S, Harvey K, Andersson A, Thennavan A, Wang C, Torpy JR, Bartonicek N, et al. 2021. A single-cell and spatially resolved atlas of human breast cancers. *Nat Genet* **53**: 1334–1347.

Zhang X, Peng L, Luo Y, Zhang S, Pu Y, Chen Y, Guo W, Yao J, Shao M, Fan W, et al. 2021. Dissecting esophageal squamous-cell carcinoma ecosystem by single-cell transcriptomic analysis. *Nat Commun* **12**: 5291.

Zhao M, Liu Y, Zheng C, Qu H. 2019. dbEMT 2.0: An updated database for epithelial-mesenchymal transition genes with experimentally verified information and precalculated regulation information for cancer metastasis. *Journal of Genetics and Genomics* **46**: 595–597. http://dx.doi.org/10.1016/j.jgg.2019.11.010.
